# PretoxTM: a text mining system for extracting treatment-related findings from preclinical toxicology reports

**DOI:** 10.1186/s13321-024-00925-x

**Published:** 2025-02-03

**Authors:** Javier Corvi, Nicolás Díaz-Roussel, José M. Fernández, Francesco Ronzano, Emilio Centeno, Pablo Accuosto, Celine Ibrahim, Shoji Asakura, Frank Bringezu, Mirjam Fröhlicher, Annika Kreuchwig, Yoko Nogami, Jeong Rih, Raul Rodriguez-Esteban, Nicolas Sajot, Joerg Wichard, Heng-Yi Michael Wu, Philip Drew, Thomas Steger-Hartmann, Alfonso Valencia, Laura I. Furlong, Salvador Capella-Gutierrez

**Affiliations:** 1 Life Sciences Department, Barcelona Supercomputing Center (BSC), Barcelona, Spain; 2 MedBioInformatics Solutions, Barcelona, Spain; 3 Hospital del Mar Medical Research Institute (IMIM), Barcelona, Spain; 4 PDS Consultants, Leicester, UK; 5 Bayer AG, In Vitro Safety, Berlin, Germany; 6 Eisai, Tsukuba, Ibaraki Japan; 7 Chemical and Preclinical Safety, Merck Healthcare KGaA, Darmstadt, Germany; 8 Translational Medicine, Preclinical Safety, Novartis Biomedical Research, Basel, Switzerland; 9 Ipsen Innovation, Les Ulis, France; 10 Roche Innovation Center Basel, Basel, Switzerland; 11 Servier, Gif-sur-Yvette, France; 12 Genentech Research and Early Development (gRED) Computational Sciences, Genentech, Inc., South San Francisco, CA USA; 13 Catalan Institution for Research and Advanced Studies (ICREA), Barcelona, Spain; 14 University of Barcelona, Barcelona, Spain

**Keywords:** Natural language processing, Text mining, Toxicology, Adverse effect, Preclinical, Animal model, Annotated corpora, Compound, Drug

## Abstract

Over the last few decades the pharmaceutical industry has generated a vast corpus of knowledge on the safety and efficacy of drugs. Much of this information is contained in toxicology reports, which summarise the results of animal studies designed to analyse the effects of the tested compound, including unintended pharmacological and toxic effects, known as treatment-related findings. Despite the potential of this knowledge, the fact that most of this relevant information is only available as unstructured text with variable degrees of digitisation has hampered its systematic access, use and exploitation. Text mining technologies have the ability to automatically extract, analyse and aggregate such information, providing valuable new insights into the drug discovery and development process. In the context of the eTRANSAFE project, we present PretoxTM (Preclinical Toxicology Text Mining), the first system specifically designed to detect, extract, organise and visualise treatment-related findings from toxicology reports. The PretoxTM tool comprises three main components: PretoxTM Corpus, PretoxTM Pipeline and PretoxTM Web App. The PretoxTM Corpus is a gold standard corpus of preclinical treatment-related findings annotated by toxicology experts. This corpus was used to develop, train and validate the PretoxTM Pipeline, which extracts treatment-related findings from preclinical study reports. The extracted information is then presented for expert visualisation and validation in the PretoxTM Web App.

**Scientific Contribution**

While text mining solutions have been widely used in the clinical domain to identify adverse drug reactions from various sources, no similar systems exist for identifying adverse events in animal models during preclinical testing. PretoxTM fills this gap by efficiently extracting treatment-related findings from preclinical toxicology reports. This provides a valuable resource for toxicology research, enhancing the efficiency of safety evaluations, saving time, and leading to more effective decision-making in the drug development process.

## Introduction

The complex process of drug development involves several phases, including target and drug discovery, preclinical development, clinical development, and regulatory approval. The entire process is very long and expensive [1]. Therefore, a major goal in the pharmaceutical industry is to use information technology, including artificial intelligence (AI), to reduce costs, accelerate timelines, and enhance the quality of drug discovery.

The preclinical development of potential drug candidates is challenging due to the vast amount of biological and chemical data that needs to be analysed. AI can greatly accelerate the discovery of promising drug candidates by efficiently processing large amounts of biological and chemical data, thereby reducing the time and expense involved in the initial phases of drug discovery [2].

Despite the need for the analysis of large data sets, a wealth of preclinical data is stored in the archives of individual companies. The limitations of data mining across these valuable closed data sources have increased the demand for data sharing and extensive data integration across various fields of preclinical research. This has led to precompetitive projects focusing on data sharing and the integration of various data sources into harmonised repositories.

eTRANSAFE [3, 4] was a research project funded by the Innovative Medicines Initiative (IMI), which aimed at the development of integrated databases and computational tools (eTRANSAFE ToxHub) that support the translational safety assessment of new drugs. This was achieved through the use of legacy data provided by the pharmaceutical companies affiliated to the European Federation of Pharmaceutical Industries and Associations (EFPIA) involved in the project. Different tools, methodologies and procedures were developed along the eTRANSAFE project life cycle in order to achieve its goals [5–8]. In this context, one of the eTRANSAFE milestones was to develop tools that are especially valuable for the preclinical phase of the drug development process.

Preclinical studies involve testing potential new drugs or treatments on animal subjects to evaluate their safety and efficacy before advancing to human clinical trials. These studies are a crucial step in the drug development process to ensure the safety and potential effectiveness of new treatments.

Toxicology studies conducted for Investigational New Drug (IND) approval aim to evaluate the correlation between the treatment dosage and its effects. In this context, a **treatment-related finding** refers to any observable effect, outcome, or manifestation that occurs as a direct or indirect result of a treatment or compound administration, which must be distinguished from spontaneous findings or background incidences. This may include intended therapeutic effects, unintended side effects, adverse reactions, or any other physiological or pathological changes attributable to the administration of a particular treatment. In addition, treatment-related findings also encompass effects observed in animal models used to predict human responses, including toxicity assessments, pharmacodynamic and pharmacokinetic studies, and pathological examinations. These preclinical findings help to identify potential risks, refine dosing regimens, and ensure that only the most promising and safe treatments proceed to clinical trials. For better readability, the expressions “treatment-related finding” and “treatment-related observation” are used interchangeably throughout the text.

**Fig. 1:**
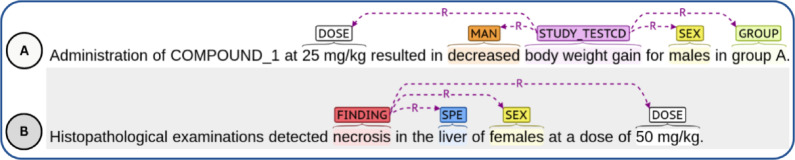
Examples of treatment-related observations and their relevant entities. **A** A treatment-related observation described by a *study test* (“body weight gain”) and the abnormal *manifestation* (“decreased”). The *dose* administered (“25 mg/kg”), the *sex* (“males”) and *group* (“group A”) of the subjects are also part of the observation. **B** A treatment-related observation described by a *finding* (“necrosis”) in a *specimen* (“liver”). The *dose* administered (“50 mg/kg”) and the *sex* (“females”) of the subjects are also part of the observation

The treatment-related observations detected on the test subjects after the administration of a compound are the kind of relevant information included in toxicology reports that is valuable for decision-making during the drug development process. A treatment-related observation recorded in these reports comprises several entities, a non-exhaustive list of which is presented in Table 1. In this regard, a treatment-related observation may be caused by a **study test** and an abnormal **manifestation**, or by an adverse **finding**. Additional relevant entities that contribute to the characterisation of a treatment-related observation include the **specimen** involved, the **sex** of the subjects, the **group** of subjects in which the abnormal observation was detected, and the **dose** of the compound being administered. Figure 1 shows examples of sentences containing treatment-related findings and their corresponding entities.

The administered compound is an essential part of the information associated with the description of a treatment-related finding but it is not included in Table 1. Toxicology studies are usually conducted on specific compounds, so this information is known in advance. Therefore, there is no need to identify the compound in a potential text mining solution. Additionally, for security reasons, pharmaceutical companies are reluctant to share information such as compound names, molecular structures, InChI keys, SMILES or any other information that could be used to identify compounds.

In the context of animal testing, study domains refer to specific categories of data that are systematically organised to capture various aspects of the study, such as clinical observations, body weights, food and water consumption, microscopic and macroscopic findings, laboratory test results, among others. Appendix A contains a compilation of study domains that were considered in this research. These domains were extracted from the Standard for Exchange of Nonclinical Data (SEND) controlled terminology [9, 10] developed by the Clinical Data Interchange Standards Consortium (CDISC) [11]. SEND is an implementation of the Study Data Tabulation Model (SDTM) for non-clinical toxicology studies and is intended to provide a structured representation of the information included in study reports.

Despite the importance of SEND, it is essential to highlight that its set of terms is limited and therefore might not encompass all potential concepts related to treatment-related findings that could appear in the toxicology reports. During the eTOX project [12], the predecessor of eTRANSAFE, efforts were made to improve and extend the controlled terminology with the OntoBrowser tool [13]. Some of the vocabularies generated have extended the CDISC SEND controlled terminology at the synonym level.

**Table 1:** The PretoxTM entity model. Named entities related to treatment-related findings

Entity	Description	Examples
Study test	Measured endpoint used to assess the effect of a compound in the animal model.	Body weight, food consumption, reticulocytes, albumin, weight
Manifestation	Manifestation of study test.	Increase, decrease, lower, changed, alteration
Finding	Adverse effect produced after the administration of the compound in the animal model.	Vomitus, hypertrophy, pale, ataxia, atrophy
Specimen	Specimen in which the effect was observed.	faeces, kidney, liver, heart, salivary gland, thymus, urine
Dose	Dose in which the effect was observed.	20 mg/kg
Sex	Sex of the animals in which the effect was observed.	Female, male, both sexes
Group	Group of animals in which the effect was observed.	Group 1, group A

Over the years, the pharmaceutical industry has generated a considerable number of toxicology reports during the preclinical phase of the drug development process. These reports are information-rich and therefore constitute one of the most important assets for pharmaceutical companies and institutions in the development of new drugs. However, most of these toxicology reports are free-text documents, containing unstructured data. These documents do not follow a common disposition or a predefined data model, and often contain images representing text (in the case of scanned documents), which significantly hampers the automatic analysis by computational tools and the extraction of the relevant information. Considering that these toxicology reports contain hundreds of pages, the process of locating and extracting evidence of treatment-related findings is complex, cumbersome and time consuming. The ability to automatically extract, analyse and aggregate such information would enable leveraging the wealth of data locked in these reports, to provide valuable new insights into the safety of drug candidates.

Text mining technologies provide a solution to analyse the large and growing amount of unstructured textual information, applying different Natural Language Processing (NLP) techniques to perform tasks such as text classification, concept recognition, summarisation, translation and question answering, among others. The continuous advances in the field of NLP, driven by the use of Transformers [14] and Large Language Models (LLMs) [15, 16], have significantly impacted the biomedical domain. These technologies have been instrumental in the pretraining of domain-specific LLMs [17–22], tailored specifically for biomedical applications. Furthermore, their seamless integration into the research and development of new drugs [23–25] has made text mining systems an essential tool to assist experts in their investigations.

In the clinical field, efforts have been made to develop text mining tools aimed at detecting adverse effects in different literary sources, such as scientific publications [26, 27], social media [28] and electronic hospital health record notes [29, 30]. However, no analogous systems have been reported for the identification of adverse events observed in animal models during preclinical testing, highlighting a significant gap in current methodologies. In the preclinical context, there have been efforts to utilise NLP to identify Adverse Outcome Pathways (AOPs) [31]. AOPs are frameworks that link molecular initiating events to toxicological outcomes through key events, supporting New Approach Methodologies (NAMs) aimed at reducing animal testing in toxicology.

The development of a specialised text mining tool tailored to the preclinical domain offers several advantages. Such a tool can facilitate the detection and analysis of adverse effects, improve the efficiency of safety evaluations, and allow researchers to save time by easily retrieving relevant information. Furthermore, a web application that focuses on easing their search and is capable of presenting results in an intuitive and user-friendly manner would significantly enhance the usability and accessibility of preclinical data, ultimately leading to more effective and timely decision-making in the drug development process.

In this manuscript, we present PretoxTM (Preclinical Toxicology Text Mining), the first system specifically designed to detect, extract, organise and visualise treatment-related findings from preclinical toxicology reports. The PretoxTM tool comprises three main components: PretoxTM Corpus, PretoxTM Pipeline and PretoxTM Web App. The PretoxTM Corpus is a gold standard corpus of preclinical treatment-related findings annotated by toxicology experts. This corpus was used to develop, train and validate the PretoxTM Pipeline, which extracts treatment-related findings using a fine-tuned Transformer model. The extracted information is then presented for expert visualisation and validation in the PretoxTM Web App.

## Implementation

### PretoxTM data model and gold standard corpus

To initiate the PretoxTM project, a series of preliminary meetings were organised to outline the objectives of the project, with particular emphasis on defining the information extraction task. These meetings included collaboration with the Study Report Domain (SR-Domain) template team. During the same period, the SR-Domain template was being developed with the aim to formally describe a treatment-related finding in a comprehensive manner. The template (Appendix D) consists of twenty-six fields representing different aspects of a treatment-related finding, based on CDISC SEND controlled terminology. The SR-Domain template and concept are copyrighted by PDS Consultants [32].

The result of these sessions was the PretoxTM Data Model described in Table 1. The data model is in a certain way a subset of fields from the SR-Domain template. It is important to note that a text mining tool that attempts to detect and extract treatment-related findings using all the fields described in the SR-Domain template is not feasible due to the large number of fields involved, the complexity, and even the fact that much of this information is in most cases not available in the text.

**Fig. 2:**
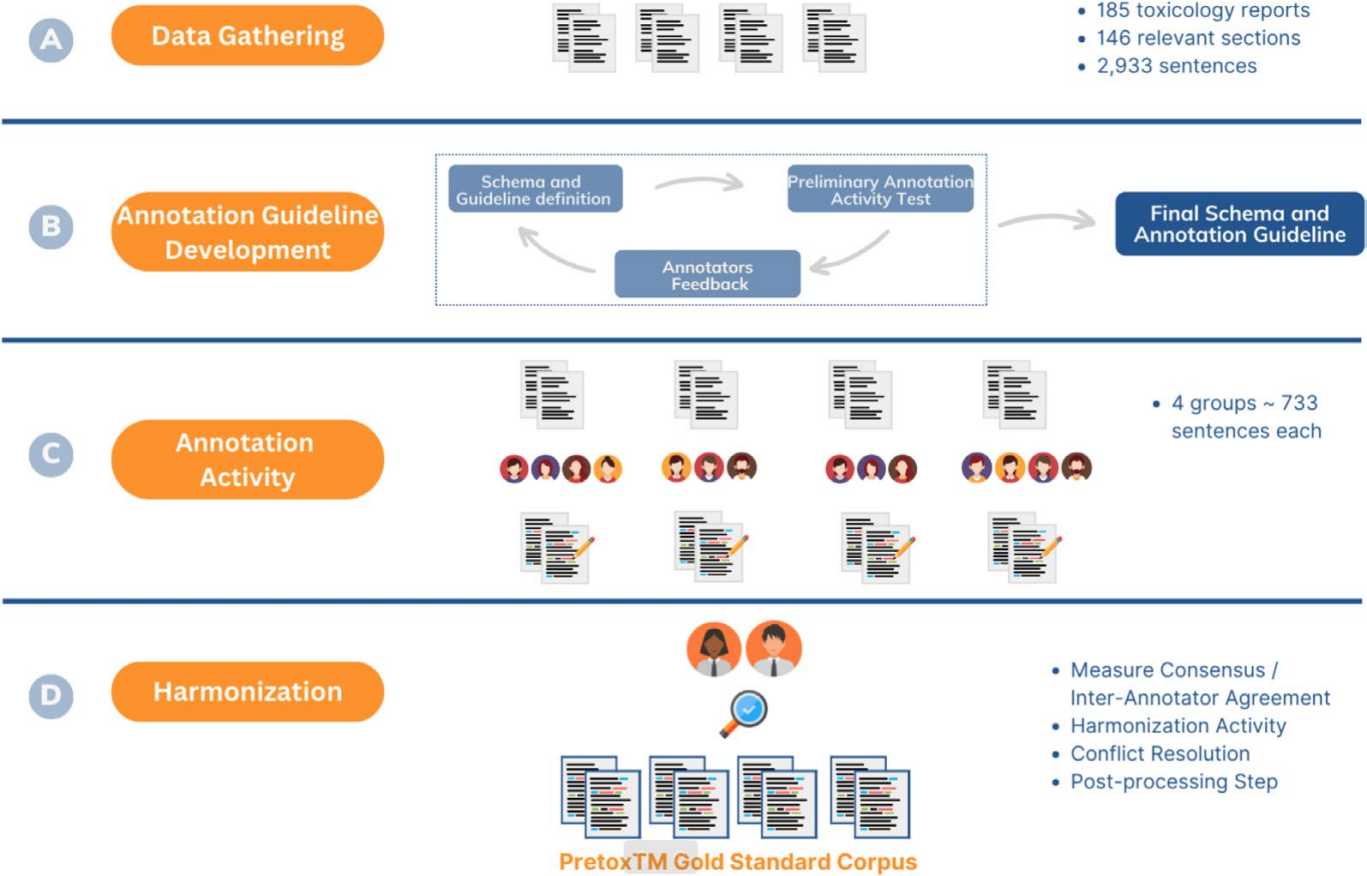
Corpus development process. **A** Data gathering of toxicology study reports in a secure centralised Nextcloud repository. **B** Annotation guideline development and annotation schema definition. **C** Annotation activity design. **D** Harmonisation and post-processing step to obtain the final annotated corpus

Example documents annotated by domain experts, also known as a gold standard corpus, are needed in order to develop, train, and validate text mining tools. Figure 2 illustrates the phases of the development of the PretoxTM Corpus, a gold standard corpus of preclinical treatment-related findings.

Toxicological studies were donated by pharmaceutical companies affiliated to EFPIA that participated in the eTRANSAFE project. A total of 185 reports were collected from seven contributors during the data gathering process (Fig. 2A). The largest contributor submitted 87 reports, followed by the second company with 48. The remaining five companies contributed almost equally, totaling 50 reports. Of all the reports received, 94 are in a readable format, while the remaining 91 are scanned documents. The shared toxicological studies were conducted between 1980 and 2017; and were distributed as follows: 3 of the studies were conducted between 1980 and 1989, 39 between 1990 and 1999, 124 from 2000 to 2009, and 19 from 2010 onwards. From the 185 toxicological reports, the text from the “Summary”, “Conclusion” and “Discussion” sections was extracted using a semi-automated approach. This process resulted in a total of 146 text sections containing 2,933 sentences.

An essential element in an annotation activity is the definition of the annotation schema and the creation of the guidelines to follow during this process (Fig. 2B). The annotation guidelines were created as a document providing a description of the annotation schema, a tutorial about the use of the annotation environment and a set of rules and tips to resolve the most recurrent issues and doubts raised by the annotators.

The initial annotation schema covered the entities of the PretoxTM Data Model described in Table 1, as well as additional entities such as effect level, date of finding, study domain, and statistical significance of the observed effect. The expert feedback received during the preliminary annotation activity made evident that the annotation schema did not meet our expectations. The schema was too exhaustive, which made it very difficult and slow to mark the multiple entities in the text and to establish all the relationships between them. Therefore, a second and final version of the annotation schema was developed to facilitate the annotation task and help to simplify the activity without compromising the expert knowledge needed to identify relevant findings. The second version focused on marking expressions rather than entities and defined two categories to be annotated: ADVERSE_OBSERVATION and CDoG (Compose Dose or Group) expressions.

The ADVERSE_OBSERVATION expression captures the manifestation of abnormal observations. It is a broad concept that may include different entities described in the PretoxTM Data Model: findings, study tests, manifestations and specimens. Examples of ADVERSE_OBSERVATION expressions are: “gained slightly less body weight”, “pale faeces were noted”, “slight increase in serum calcium”, and “minimal to slight mineralization of the kidneys”. The CDoG expressions describe the treatment in terms of the administered dose or the group of animals associated with the findings. Examples of CDoG expressions are “20 mg/kg/day”, “group A”, “high dose group”, and “12 mg/kg/day dose group”.

A tool was needed that would allow annotations to be created from any device equipped with a web browser. Furthermore, these annotations needed to be stored on a central server for efficient processing. After evaluating the required functionality and reviewing existing annotation tools [33], WebAnno [34] was selected as the annotation software.

The annotation process (Fig. 2C) lasted eight weeks, in which follow-up weekly meetings were held in order to track progress as well as answer questions related to the use of the annotation tool and the annotation guideline. For this activity, a team of 14 experts participating in the eTRANSAFE project was recruited.

The 2933 sentences were divided equally into four sets, with approximately 733 sentences per set. Each set was annotated by a different group, consisting of two groups of three annotators and two groups of four annotators. This design for the annotation activity aimed to ensure that each relevant section was annotated by at least three experts.

**Table 2:** PretoxTM Gold Standard Corpus statistics

Category	Count
Relevant sentences	1264
Non-relevant sentences	1669
Study Test	1150
Manifestation	1044
Finding	1880
Specimen	1395
Dose	1322
Sex	748
Group	341
Tokens	86,421
Sentences	2933

Calculating and analysing the results of the inter-annotation agreement (IAA) was essential to identify the differences that emerged between the experts during the annotation activity. The IAA was calculated using Krippendorff’s Alpha with the nominal/strict method [35, 36]. We first computed the IAA for each of the four groups and then averaged the results, obtaining 0.73 for ADVERSE_OBSERVATION expressions, 0.74 for CDoG expressions and 0.76 in general. Table 3 shows the complete results.

**Table 3:** Inter Annotator Agreement using Krippendorff’s Alpha

	ADVERSE_OBSERVATION	CDoG	General
Group 1	0.67	0.70	0.70
Group 2	0.76	0.75	0.78
Group 3	0.80	0.76	0.80
Group 4	0.70	0.75	0.74
AVG	0.73	0.74	0.76

Following the annotation activity, a harmonisation process was conducted with the aim of generating a consensus corpus among the different annotations made by the experts (Fig. 2D). The harmonisation process consisted of reviewing the annotations made by the experts and selecting the final annotation, always conforming to the criteria described in the annotation guidelines.

Conflicts that were not contemplated in the guideline or that implied a decision on toxicological aspects were resolved in conflict resolution meetings, where two toxicology experts had the final decision on the dispute. After resolving all conflicts, a post-processing step was applied to automatically standardise the ADVERSE_OBSERVATION and CDoG expressions by mapping entities defined in the PretoxTM Data Model. A final review by an expert was conducted to ensure that entities were accurately mapped to the expressions. In addition, a corpus of sentences was generated based on the premise that any sentence containing an abnormal observation is considered relevant, otherwise it is considered non-relevant. Table 2 provides a detailed overview of the PretoxTM Corpus, along with additional relevant information.

Additional and detailed information about the development of the PretoxTM Corpus is available in the supplementary materials, along with the Annotation and Harmonization Guidelines.

The PretoxTM Corpus is publicly available and free to access on Zenodo at https://zenodo.org/record/7858116; and on HuggingFace at https://huggingface.co/datasets/javicorvi/pretoxtm-dataset. The PretoxTM Corpus is licensed under a CC BY-SA 4.0 licence.

### PretoxTM pipeline

The PretoxTM pipeline was developed using different Natural Language Processing (NLP) techniques to detect and extract findings from toxicology study reports into a structured format. The PretoxTM Pipeline accepts input data consisting of toxicology study reports in PDF format or simple TXT files. The generated output is a collection of observations presented in CSV format, facilitating further exploration and analysis.

**Fig. 3:**
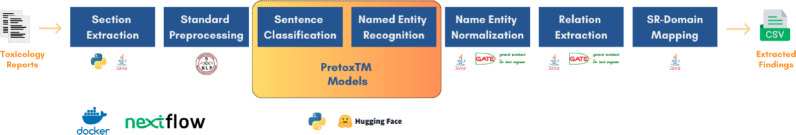
The PretoxTM Pipeline. Steps include the extraction of relevant sections; standard text preprocessing; classification of relevant sentences and named entity recognition using the PretoxTM Models; the normalisation of named entities to different vocabularies; relation extraction, which identifies entities that belong to the same abnormal observation; and finally SR-Domain format mapping. The output of the pipeline is a list of findings in CSV format

There are several steps involved in the execution of the pipeline, each of which is described in more detail below: Section Extraction, Standard Preprocessing, Sentence Classifier, Named Entity Recognition (NER), Named Entity Normalisation (NEN), Relation Extraction (RE) and SR-Domain Mapping. Figure 3 shows the architectural overview of the PretoxTM Pipeline.

#### Section extraction

The first step in the pipeline involves extracting relevant sections with recorded findings from PDF study reports. This step is optional, as the pipeline can be configured to process the entire report, though this is not recommended due to the length of toxicology documents. If the input is a plain TXT file, this extraction step is skipped.

The section extraction component for PDF study reports works in different scenarios. For readable PDFs, it identifies sections of interest by soft-matching a list of section titles, such as “Summary” and “Results” and extracts their contents into plain TXT files. This is done by using a Python module to locate relevant sections via PDF bookmarks or the table of contents, followed by post-processing to remove irrelevant information. If this fails, the PDF is converted to XML/TEI format, and the GROBID library [37] is used for extraction. Tesseract [38, 39], one of the most well-known open-source OCR engines, is used for text recognition in scanned PDFs. Modern OCR systems, including Tesseract, perform with very high accuracy on relatively clear images [40]. However, the challenge arises when scanned documents are in poor condition. In such cases, performance declines in proportion to image quality. Studies on low-quality images show that Tesseract achieves an accuracy of 70.2% [41]. Nevertheless, these tools are under continuous development, constantly striving for improvement. In cases of mixed PDFs with both scanned images and readable pages, only the readable pages are processed.

#### Standard preprocessing

The main goal of this component is to apply common NLP tasks such as Sentence Splitting, the process of dividing text into sentences; Tokenization, the process of turning text into tokens-words; Part of Speech Tagging, assigns Part-Of-Speech labels to tokens (verbs, nouns, punctuation, etc.); Lemmatization, maps a word to its dictionary lemma (decreased basophils$\rightarrow$decrease basophil); and other features extracted from the text (tokens length/shape, sentences length). The inputs of this component are plain TXT files with the text to be processed, and the outputs are JSON files containing the sentences and tokens annotations, including the explained features. To develop this component we used both the Stanford CoreNLP framework [42] and the General Architecture of Text Engineering (GATE) [43].

#### Sentence classifier

Since the aim of PretoxTM is to detect abnormal observations, the next step in the pipeline is to identify relevant sentences. The goal of the sentence classifier is to automatically assign the label “toxicologically relevant” or “toxicologically non-relevant” to a given input sentence based on its content, meaning or context. For this purpose, we developed the PretoxTM Sentence Classifier model using transformers [14]. Transformers use a mechanism known as the self-attention to process input data in parallel, allowing them to capture complex relationships in text data.

BERT (Bidirectional Encoder Representations from Transformers) [15] is a transformer-based model, specifically designed for tasks that require understanding of context from both left and right directions in a sentence. BERT has been extended for various specialised domains to improve performance on specific tasks, such as biomedical [17, 20, 22], clinical [19], legal [44], finance [45] and chemistry [46], among others.

We used the pre-trained BiomedBERT model [22] fully named as “microsoftBiomedNLP-BiomedBERT-base-uncased-abstract-fulltext”, which has been pre-trained on PubMed abstracts and full-text articles, and fine-tuned it on our PretoxTM Corpus of sentences (Table 2).

**Fig. 4:**
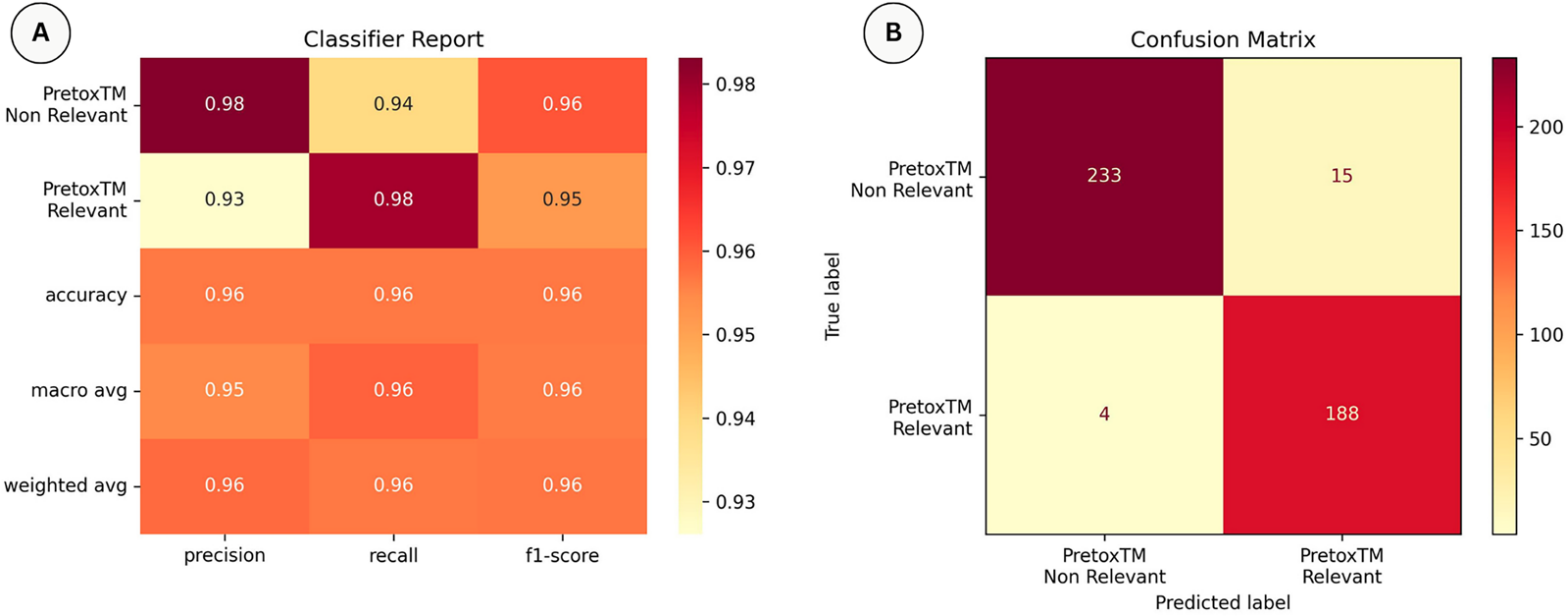
**A** Performance of the PretoxTM Sentence Classifier, resulting from fine-tuning the BiomedBERT pre-trained model. Precision, recall and F1-score for each of the two categories: PretoxTM Relevant and PretoxTM Non-relevant; followed by the average accuracy, macro-average and weighted average. **B** Confusion matrix of the two categories from the validation test set. Hyperparameter optimisation: number of training epochs = 3, learning rate = 1.1848183151867784e$-$05 and seed = 1 (for reproducibility)

We developed our PretoxTM Sentence Classifier model using 70% of the sentence dataset (2053 sentences) for the training set, 15% (440 sentences) for the validation set used during hyperparameter optimisation, and another 15% (440 sentences) for the test set. Figure 4A shows the PretoxTM Sentence Classifier performance evaluated on the test set, with a precision, recall and F1-score of 0.96. Additionally, Fig. 4B shows the confusion matrix generated from the test set. Among a total of 440 sentences, 19 were misclassified, resulting in an error rate of 4.32%.

The best model was selected after a benchmarking on different biomedical pre-trained models from the BERT family including BiomedBERT [22], BioBERT [17], Bio_ClinicalBERT [19] and BioDistilBERT-uncased [20]. In order to find the best possible performance for each pre-trained model, we applied hyperparameter optimisation by running 50 trials. In each trial, the hyperparameters were systematically varied within predefined ranges and the performance of the model on the validation set was evaluated. During hyperparameter optimisation, we implemented L2 regularisation [47] by setting the weight decay parameter to 0.01. This technique is used to prevent overfitting, which refers to the tendency of the model to over-optimise on the training data, by adding a penalty term to the loss function that discourages large weights. Early stopping was also used by monitoring the validation loss function. This method not only prevents overfitting, but also improves training efficiency. Appendix B shows the performance of the optimised configuration for the remaining pre-trained models that were tested.

This component was developed in Python leveraging the HuggingFace ecosystem for the development of transformer models and it is available at https://huggingface.co/javicorvi/pretoxtm-sentence-classifier.

#### Named entity recognition

Once the relevant sentences are identified, the next step is the Named Entity Recognition (NER) of concepts related to an abnormal observation (Table 1). For this purpose, we developed our PretoxTM NER model using the same strategy explained in the Sentence Classifier 2.2 section, dividing the dataset into training (70%), validation (15%) and test (15%).

The best model was obtained by fine-tuning the pre-trained BioBERT (Bidirectional Encoder Representations from Transformers for Biomedical Text Mining) model [17]. We fine-tuned the BioBERT model, pre-trained with PubMed for 1 M steps, also known as BioBERT v1.1 (+PubMed 1 M). For the development of the PretoxTM NER model, we performed a hyperparameter optimisation of 100 trials for each pre-trained model tested, exceeding the number used for sentence classification. This discrepancy in the number of trials is due to the different levels of complexity involved in finding the best performance in these tasks. NER models require a more exhaustive search for the optimal configuration.

Table 4 shows the NER metrics of the model using exact match. The overall results, including all entities, are satisfactory; a precision of 0.81, recall of 0.86 and F1-score of 0.84. However, in the context of this task, particular attention should be paid to the following entities: “Study Test”, “Manifestation”, “Finding” and “Specimen”, which are the essential in our data model.

Terms belonging to the CDISC SEND controlled terminology are usually used to describe “Study Tests” in toxicology reports, and therefore the results are more accurate (F1-score of 0.88) than in the “Finding” field (F1-score of 0.77), which is used to capture abnormal effects from study domains where no study tests are defined, such as the clinical signs, microscopic and macroscopic domains, where observations often appear in an informal writing style. Besides F1-score, in our design and development of the NER model, recall of entities is given more importance than precision. We prefer a high recall of abnormal observations at the expense of precision, as it is the expert who ultimately validates whether or not an observation has been correctly recognised.

Appendix C displays the results obtained from the best hyperparameter configuration for the remaining pre-trained models that were tested.

This component was developed in Python leveraging the HuggingFace ecosystem for the development of transformer models and it is available at https://huggingface.co/javicorvi/pretoxtm-ner.

**Table 4:** PretoxTM NER model performance resulting from fine-tuning the BioBERT pre-trained model. Hyperparameter optimisation: number of training epochs = 7, learning rate = 5.760003080365119e$-$05 and seed = 4 (for reproducibility)

Entity	Precision	Recall	F1-score
Study test	0.87	0.90	0.88
Manifestation	0.89	0.93	0.91
Finding	0.74	0.80	0.77
Specimen	0.76	0.83	0.79
Dose	0.89	0.88	0.88
Sex	0.87	0.94	0.90
Group	0.86	0.91	0.89
Overall	0.81	0.86	0.84

#### Named entity normalisation

Named Entity Normalisation (NEN), or Named Entity Linking (NEL), is the process of mapping the identified named entities to a knowledge base or reference data set. This is done to provide additional context and information about the entities mentioned in the text by linking them to the appropriate entry in a well-established lexical resource (vocabulary). Moreover, the process of NEN facilitates interoperability and understanding between different systems and applications. We have developed the PretoxTM_NEN component using different lexical resources, see Table 5. Among these, we can highlight the use of the CDISC SEND controlled terminology.

The key entities of an abnormal observation (“Study Test”, “Finding” and “Specimen”) should ideally be linked using the CDSIC SEND controlled terminology, although its limited terms may not cover all possible toxicology study concepts. To address this, other resources such as the eTOX terminologies [12, 13] and the Unified Medical Language System (UMLS)[48] were included. In addition, internal dictionaries (PretoxTM Terminology) were used to map simpler concepts such as “Manifestation” and “Sex”.

To complete the NEN process, lexical rules using JAPE (Java Annotation Patterns Engine) [49] were used for post-processing functions, such as removing false positives, merging annotations and determining the priority between the different categories and sources. JAPE operates on annotations based on regular expressions and is part of the GATE framework.

**Table 5:** Lexical resources used during the NEN

Entity	Lexical resource/mapping strategy
Study Test	CDISC SEND, eTOX LBTEST Synonyms Extension
Manifestation	PretoxTM terminology
Finding	CDISC SEND, eTOX In-life-observation and Histopathology ontology and UMLS
Specimen	CDISC SEND, eTOX Anatomy ontology and UMLS
Dose	Lexical Rules Mapping
Sex	PretoxTM terminology
Group	Lexical Rules Mapping

#### Relation extraction

The relation extraction process entails the identification of the entities that belong to the same abnormal observation. This process is supported by a set of lexical rules using JAPE with different priorities that link together the “Finding” or the “Study Test” with the rest of the relevant entities: “Manifestation”, “Specimen”, “Sex”, “Group” and “Dose” when possible, given that sometimes this information is not present in the text. During the process, entities within the same sentence are considered to generate a relationship. However, there are exceptions for three specific entities-“Sex”, “Dose”, and “Group”-where, if they are not detailed in the sentence, the search for the relationship extends to the previous sentence. To achieve this, the process applies specific rules to identify correlations between the entities of interest. These rules make use of patterns, Part-Of-Speech analysis, and other lexical features to identify the relationships.

In addition, an important aspect of relation extraction is detecting whether a finding is treatment-related. To achieve this, the process applies specific rules to identify correlations between an abnormal observation and treatment-related trigger expressions such as “treatment-related”,“ compound-related effect”, and “attributable to treatment”, among others.

#### SR-domain mapping

The SR-domain mapping component is the final step in the PretoxTM pipeline. Its objective is to map detected abnormal observations from the PretoxTM Data Model format into the SR-Domain template. This post-processing step assigns and populates the corresponding values of the SR-Domain template whenever possible. Finally, it generates the results of the PretoxTM pipeline in two formats: a CSV file containing the observations and another CSV file with the SR-Domain template.

### PretoxTM Web App

The PretoxTM Web App was developed to provide toxicologists with a graphical user interface (GUI) that enables the necessary functionality to run the PretoxTM Pipeline and validate the results automatically extracted from toxicology reports using a simple and interactive interface design. The PretoxTM Web App combines the PretoxTM UI (frontend), business logic and database access (backend) through various REST APIs with the main objective of providing user-friendly interactive services through a web browser to perform the entire process of detecting treatment-related findings from study reports. Broadly speaking, the Web App enables the upload of study reports, the execution of the PretoxTM Pipeline, and the visualisation and curation of the extracted findings.

**Fig. 5:**
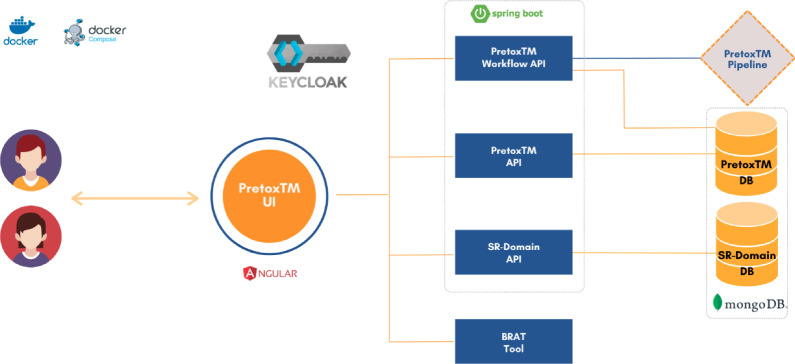
PretoxTM Web App solution architecture. The PretoxTM UI interacts with different REST APIs. The PretoxTM Workflow API is responsible for connecting and executing the PretoxTM Pipeline module. The PretoxTM API delivers the common requests for manipulating the reports and visualising the information of the findings. The SR-Domain API is responsible for delivering the SEND CDISC controlled terminology. The BRAT tool is a third party service that provides the visualisation of the textual evidence of the extracted findings. Keycloak is used as the Identity and Access Management (IAM) solution. It provides features including single sign-on (SSO), user authentication, authorisation, and user account management. The PretoxTM Web App solution uses Docker Compose to define, manage and orchestrate the deployment of multiple Docker containers

A web application should meet certain requirements, focusing on being modular, scalable, robust and secure, among other software engineering principles. Figure 5 shows the architecture of the PretoxTM Web App designed to comply with these requisites.

The PretoxTM UI was developed using AngularJS following the Single Page Application (SPA) pattern. The Web App interacts with the user by dynamically updating the current page rather than loading entire new pages from the server, providing a smoother and more responsive user experience by reducing the need for full page reloads. This component interacts with different REST APIs that are responsible for different tasks. The PretoxTM Web App backend platform comprises three RESTful APIs designed to streamline the manipulation and curation of reports, workflow execution, and controlled terminology delivery.

The PretoxTM API serves as the foundational component for manipulating and curating data within the PretoxTM Web App. This RESTful API provides endpoints to upload, list, visualise, and remove reports. This API facilitates most operations in the PretoxTM Web App, enabling users to manage and curate their data effectively.

The PretoxTM Workflow API manages the connection and execution of the PretoxTM Pipeline module. It allows users to initiate workflows and, upon completion, automatically stores the results in the PretoxTM DB. Additionally, it provides an overview of historical workflow executions and supports the cancellation and deletion of workflows, ensuring comprehensive workflow management.

The SR-Domain API is responsible for delivering the SEND CDISC controlled terminology. This module is periodically synchronised with the CDISC SEND repository, ensuring up-to-date terminology stored in the SR-Domain DB. It enables users to access and utilise standardised terminology for their data needs.

All three APIs were developed in Java using the Spring ecosystem, including Spring Boot, to simplify the development and deployment of REST APIs. Swagger was utilised for API documentation and testing, ensuring a clear and interactive API specification.

BRAT [50] is a third-party service essential for visualising textual evidence extracted from observations. It serves as a robust tool for rendering and interpreting textual data in a graphical format, enhancing the comprehension and analysis of extracted information. To integrate BRAT seamlessly into our system, an AngularJS component was specifically developed. This component embeds the BRAT visualisation tool directly into our application interface, allowing users to interact with and explore textual evidence conveniently within their workflow.

Keycloak serves as the Identity and Access Management (IAM) solution for the PretoxTM Web App. It facilitates single sign-on (SSO), user authentication, authorisation, and user account management. Keycloak secures the PretoxTM REST APIs, acting as an Identity Provider (IDP) with OAuth 2.0 for authentication and authorisation.

### PretoxTM deployment and availability

Scientific software applications face challenges in sharing, distributing, and running systems easily, as well as replicating research results. To address these issues, the PretoxTM Pipeline was developed using Docker for software containerisation and Nextflow as the workflow manager. Docker compartmentalises each subcomponent with its dependencies and programs in isolated containers, while Nextflow automates the orchestration and execution of the pipeline. This architecture allows the tool or its components to be easily installed and run in diverse environments. The PretoxTM Pipeline is available at https://gitlab.com/pretoxtm/pretoxtm-pipeline.

Similarly, the PretoxTM Web App solution uses Docker containers for individual component isolation and Docker Compose for defining, managing, and orchestrating multiple containers. Docker Compose configures environment variables, ports, volumes, and networking options for each container, simplifying the development and deployment of multi-container applications. The PretoxTM Web App is available at https://gitlab.com/pretoxtm/pretoxtm.

## Results

The PretoxTM Web App allows the user to upload toxicology documents, run the PretoxTM Pipeline, visualise and validate the extracted findings, and export the results, among other tasks. This section describes the main features of the PretoxTM Web App and uses an example toxicology report to demonstrate the benefits of using PretoxTM to extract treatment-related observations.

**Fig. 6:**
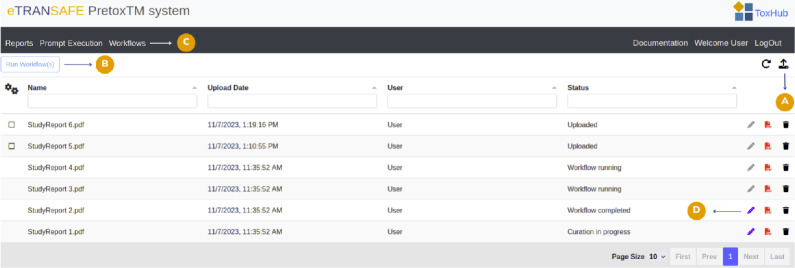
The PretoxTM Web App home page. **A** Upload documents button. **B** Start an execution of the PretoxTM pipeline. **C** Menu options “Reports”, “Prompt Execution” and “Workflows”. **D** Open a document for validation and curation

The PretoxTM home page displays a list of toxicology documents that are currently being processed (Fig. 6). The columns of the table show relevant features of each document: the file name (Name), when it was uploaded (Upload Date), who uploaded it (User) and its current status within PretoxTM (Status). In addition, the last column contains a series of action buttons: a blue pencil icon that allows validation and curation as the document passes through the PretoxTM Pipeline, an icon to open the original document and a black trash icon to remove it from the system.

The user can upload one or more documents in PDF or TXT format using the upload icon at the top right of the page. Once the documents have been uploaded, a dialogue box will appear allowing the user to select which documents to process immediately using the PretoxTM Pipeline. Alternatively, the user can run the PretoxTM Pipeline by selecting one or more documents in “Uploaded” status and using the “Run Workflow” button located at the top left of the panel.

If preferred, the user can access the “Prompt Execution” menu to enter text in a designated field and then execute the workflow. This feature provides a convenient way to rapidly test the capabilities of the tool. In this scenario, the system automatically generates a document in TXT format, which is listed as a regular PretoxTM document.

Once the user has started a workflow, the status of the selected documents will change to “Workflow running”. In our example “StudyReport 3.pdf” and “StudyReport 4.pdf” are being processed by the PretoxTM Pipeline. In addition, the PretoxTM Pipeline executions can be reviewed in the “Workflows” panel.

When a workflow is initiated while another workflow is already in progress (indicated by the status “Workflow running”), the new workflow and the associated reports are assigned the status “Workflow waiting”. Consequently, workflows are queued until the preceding one has completed.

After the completion of the PretoxTM Pipeline execution, the status of the documents is set to “Workflow completed”. In the example, the “StudyReport 2.pdf” has already been processed by the pipeline and is ready for analysis. The user can then open a report by clicking on the blue pencil icon to visualise and review the findings. When a report is opened, the status is automatically set to “Curation in progress”, as can be seen in the case of “StudyReport 1.pdf”.

**Fig. 7:**
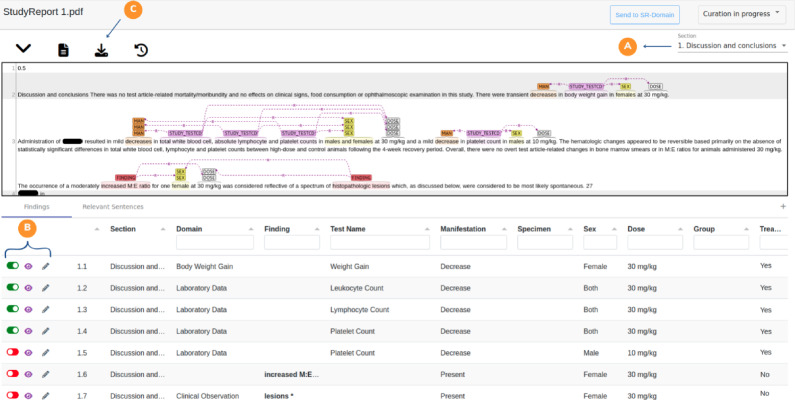
An open PretoxTM document displaying a table with extracted observations and corresponding textual evidence. **A** Selection of the section of interest. **B** Operations to perform on the observations: Accept, Reject, Visualise Evidence, and Edit. **C** Export observations in CSV format

The “StudyReport 1.pdf” document is used to analyse the results obtained after executing the PretoxTM Pipeline (Fig. 7). The first information shown when opening a PretoxTM document is the table describing the abnormal observations extracted by the PretoxTM Pipeline. Each row describes an abnormal observation; starting with the “Section” where it was detected and the “Domain” of the abnormal observation. Depending on the Domain, the abnormal observation can be given by a “Test Name” and an abnormal “Manifestation”, or by an abnormal “Finding”, in domains where there is no associated “Test Name”. The rest of the columns contain relevant information such as the “Specimen” of the abnormal observation, the “Sex” of the subject, the “Group” of subjects in which the observation was detected and the “Dose” administration of the compound. Finally, the “Treatment Related” column indicates if the abnormal observation is treatment-related or not.

In order to visualise the textual evidence of the extracted findings, the user must select a specific section of interest. In this example, “Discussion and conclusions” is selected and its textual evidence is displayed. The textual evidence panel depicts in a qualitative manner the abnormal observations, highlighting the relevant entities with different colours and tooltips, and creating the links between them to describe associations.

It is important to note that the abnormal observations listed in the table are already mapped to the SR-Domain format. For example, the first observation 1.1 outlined in the table is related to the sentence number 2 of the textual evidence panel; “decrease”→Decrease in “body weight gain”→Weight Gain in “females”→Female at the “30 mg/kg”→30 mg/kg dose. Based on the textual evidence, we can conclude that this information is complete and has been well identified and reported. This is the methodology to be followed to validate the extracted observations: review the information reported in the table and decide, based on the textual evidence, whether an observation was well detected or not. For that aim, the user has a mechanism to validate the findings; in the first column, each observation has a slide-check to indicate whether it is accepted (green) or not (red). By default, all the observations are marked as not valid to prevent storing incorrect information.

Continuing with the example, sentence 3 found in the textual evidence panel contains several abnormal laboratory domain data observations that are described in a slightly more complex style of writing. These observations are detailed in the table under the identifiers 1.2, 1.3, 1.4 and 1.5. The first three observations are separated by coordinators and refer to the study tests “total white blood cell”→Leukocyte Count, “absolute lymphocyte”→Lymphocyte Count and “platelet counts”→Platelet Count. These study tests are linked to the same abnormal manifestation “decrease”→Decrease, the same sex “males and females”→Both, and the same dose “30 mg/kg”→30 mg/kg. Observation 1.5 is also a description of a decreased platelet count study test but only for males at the 10 mg/kg dose. Sentence 3 illustrates how complex toxicity phrases can be present in the preclinical literature and how the PretoxTM system can detect these complexities to extract abnormal observations correctly.

Non-SEND controlled terms may appear in the “Study Test”, “Finding” and “Specimen” entities. This is highlighted in the table in bold and with an asterisk. The findings in 1.6 and 1.7, “increased M:E ratio”→increased M:E ratio and “histopathologic lesions”→lesions have been normalised to the eTOX terminology.

To resolve situations involving non-SEND terms, or to complete or modify a particular field of an observation, the user can edit the information of a non-validated observation. The pencil icon in the first column of the table enables editing of the corresponding row. The user can change the values of the fields by clicking on them. The information can then be saved or discarded by clicking on the appropriate button in the first column. This allows the user to enter missing information, modify an erroneously detected value, or even edit non-SEND terms and assign them a value from the SEND terminology. The latter case is possible because a select box with the different values will be displayed for the user to provide the desired term. It is important to note that modifications made by editing an abnormal observation in the table will not affect or be reflected in the textual evidence.

Once curation is complete, the user can export the validated findings by clicking the download button in the report menu. This operation will download two files: a CSV file containing the abnormal observations and another CSV file with the SR-Domain template. An example of these files can be found in Appendix E.

Finally, the document can be moved to “Curation finished” status to indicate that work on the document has been completed.

The PretoxTM Web App has been specifically designed for toxicologists, providing them with a user-friendly graphical interface. This interface facilitates the execution of the PretoxTM Pipeline and the validation of results extracted from toxicology reports, all through a simple and interactive design. However, for users who prefer to run the PretoxTM Pipeline without relying on the web interface, they have the option to execute it directly as a standalone application.

For detailed information about the PretoxTM system and all its features, including use cases and practical examples, please refer to the PretoxTM User Manual. The user manual, along with an introductory video, is available in the supplementary materials.

## Discussion

The value of PretoxTM lies in its ability to facilitate the recognition and extraction of treatment-related or noteworthy observations identified in animal studies conducted during compound testing and recorded in toxicology reports. This is particularly important given the challenges associated with the large number of historical toxicology studies available in pharmaceutical companies and their poor digitisation. Many of these reports are extensive, lack a defined format, contain scanned images, and do not adhere to controlled terminology standards such as CDISC SEND. In this context, PretoxTM becomes a powerful tool that simplifies the extraction task, saving resources and time.

PretoxTM is the first text mining system using advanced NLP techniques designed to identify treatment-related findings in preclinical toxicology reports. In addition to the text mining solution, the PretoxTM Web App allows reports to be processed in a simple way, without the need for any experience in the execution of workflows. Processing is completely transparent to the user. Most importantly, the PretoxTM Web App provides experts with the necessary support to review the detected findings through a summary, with the possibility to indicate whether they have been correctly detected or not.

There are notable differences between observations recorded in human studies and those observed during the preclinical process in animals. These discrepancies often arise due to the inherent biological differences between humans and animals, which can lead to variations in how adverse effects manifest. Additionally, the terminology and writing style used in preclinical research differs significantly from those used in clinical research. In particular, systems developed for clinical adverse event mining [26–30] cannot be applied to preclinical research because the endpoints measured and the types of documents used are different. Moreover, no analogous systems have been reported for the identification of adverse effects observed in animal models during preclinical testing. Consequently, the lack of analogous systems and the significant differences between the preclinical and clinical domains were key factors in the decision to develop a tool specifically tailored to detect adverse effects in the preclinical literature.

The PretoxTM project has not only focused on the development of a toxicology text mining tool, but has also invested time and effort in the development of a preclinical corpus that can be used as a benchmark for other studies. NLP technologies, techniques and models are evolving rapidly, whereas an expertly curated dataset will endure over time to serve as a gold standard for the development of new tools. The PretoxTM Corpus is open access, with the ultimate goal of facilitating the development of new solutions.

A key limitation to address is the relatively small size of the PretoxTM Corpus, which may affect the performance of the models by limiting diversity and reducing generalisation capability. Text mining models typically require a substantial amount of training data to perform optimally, and a smaller dataset may hinder their ability to capture broader patterns or variations in the data.

However, the process of collecting such data is often fraught with challenges, particularly in industries such as pharmaceuticals where information sharing between competing companies can be challenging. Despite these obstacles, a satisfactory number of toxicology data were collected, forming the basis of the PretoxTM Corpus. Augmenting this corpus with additional observations is both costly and time consuming. Future work could explore techniques such as data augmentation to increase the size of the PretoxTM Corpus. Data augmentation techniques depend on the specific NLP tasks; different approaches are used for text classification [51, 52] and for NER [53, 54]. In particular, the incorporation of new training data would enhance the performance of the NER model, especially for certain entities such as “Finding” (F1-score 0.77) and “Specimen” (F1-score 0.79), which have potential for improvement.

The reports used to build the corpus were donated by seven pharmaceutical companies in varying proportions. These reports can differ significantly in structure and grammatical format depending on the contributor. Furthermore, grammatical and lexical differences can be observed among different study directors within the same company or may depend on the date of report preparation. It is important to note that the corpus only reflects the variability and grammatical structure based on these donations; preclinical literature from different institutions may vary significantly in style, terminology, and presentation. As a result, PretoxTM models trained on this specific corpus may encounter challenges when interpreting or analysing texts that do not adhere to the same patterns or conventions as the donated reports, potentially affecting their overall performance and applicability to broader datasets.

It is important to emphasise that while PretoxTM automatically analyses and extracts abnormal observations from preclinical reports, it is designed to assist toxicologists in identifying and describing findings. For safety reasons, the information provided by the tool must be manually validated by toxicologists. PretoxTM streamlines this validation process by presenting the detected findings alongside the relevant textual context, enabling toxicologists to more efficiently review and confirm the accuracy of the observations within the appropriate context.

The full version of the PretoxTM system is designed specifically for toxicology reports. However, its component-oriented design allows for the independent use of various components without the need to install the entire PretoxTM software suite. Among these components are the PretoxTM Sentence Classifier, which identifies relevant toxicological sentences, and the PretoxTM NER Model, which detects concepts related to treatment-related findings. Both models are freely available for integration into text mining solutions, enhancing their accessibility and utility beyond the PretoxTM system.

Future work in PretoxTM will involve experimenting with another type of LLMs, specifically from the Generative Pretrained Transformer (GPT) family. Currently, PretoxTM uses fine-tuned versions of BiomedBERT and BioBERT, which are pre-trained models from the BERT family, specialised in biomedical literature. While LLMs from the BERT family still outperform GPT models in tasks such as classification and NER, advancements in GPT-based models are rapidly progressing [55, 56]. This provides a strong motivation to explore their potential in future PretoxTM releases.

When a consortium comes to an end, ensuring sustainability is crucial to maximise the impact of the project’s outcomes. In the case of eTRANSAFE, the set of databases and computational tools, including PretoxTM, were acquired by a private IT company that develops biomedical solutions. This acquisition ensures that the work done during the eTRANSAFE project will be sustained over time, allowing for significant improvements and even the development of new tools.

In future iterations, PretoxTM will expand its capabilities to detect the relevant data necessary for constructing virtual control groups (VCGs) in preclinical studies [57, 58]. This development will be conducted within the context of the VICT3R project (VIrtual Control groups To reduc3 animal use in toxicology Research). The overarching objective of VICT3R is to significantly reduce the number of animals used in experimental studies conducted during nonclinical drug and chemical safety evaluations by substituting animals in concurrent control groups (CCGs) with Virtual Control Groups (VCGs). PretoxTM is intended to play a crucial role in the detection and curation of information for the generation of VCGs.

## Conclusions

An important source of information in the drug development process are treatment-related findings detected on test subjects after administration of the compound during the preclinical phase. These findings are recorded in toxicology study reports, most of which do not follow a common disposition and are free text documents containing unstructured data. The process of locating and extracting evidence about treatment-related findings in these reports is complex, cumbersome and time-consuming. Over the years, the pharmaceutical industry has generated a significant amount of toxicology reports and this data continues to grow.

We have introduced PretoxTM (Preclinical Toxicology Text Mining), a novel system specifically designed to detect, extract, organise and visualise treatment-related findings from the toxicology literature. The PretoxTM tool consists of three main components: PretoxTM Corpus, PretoxTM Pipeline and PretoxTM Web App. The PretoxTM Corpus is a gold standard corpus of preclinical treatment-related findings annotated by toxicology experts. This corpus has been used to develop, train and validate the PretoxTM Pipeline, which extracts treatment-related findings using a fine-tuned Transformer model. The extracted information is then presented in the PretoxTM Web App for expert visualisation and validation.

The challenges posed by unstructured toxicology reports in drug development have been addressed with the introduction of PretoxTM. By applying advanced NLP techniques, PretoxTM efficiently extracts treatment-relevant findings from these reports, providing a valuable resource for toxicology research. PretoxTM was developed as part of eTRANSAFE, a major European research project involving several pharmaceutical companies. PretoxTM and all its components are open source and freely available, encouraging collaboration and innovation in the field of preclinical toxicology NLP.

## Supplementary Information

Additional file 1.

Additional file 2.

Additional file 3.

Additional file 4.

Additional file 5.

## Figures and Tables

**Fig. 8:**
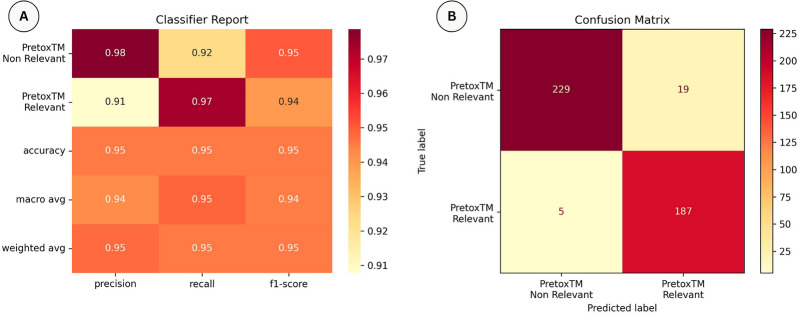
**A** Sentence classification performance resulting from fine-tuning the BioBERT pre-trained model. Precision, recall and F1-score for each of the two categories: PretoxTM Relevant and Non-PretoxTM Relevant; followed by the average values accuracy, macro average and weighted average. **B** Confusion matrix of the two categories from the test set. Hyperparameter optimisation: number of training epochs = 8, learning rate = 6.01260334773679e$-$06 and seed = 34 (for reproducibility)

**Fig. 9:**
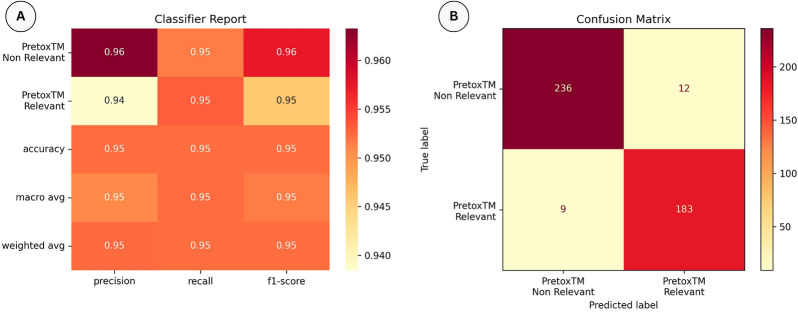
**A** Sentence classification performance resulting from fine-tuning the Bio_ClinicalBERT pre-trained model. Precision, recall and F1-score for each of the two categories: PretoxTM Relevant and Non-PretoxTM Relevant; followed by the average values accuracy, macro average and weighted average. **B** Confusion matrix of the two categories from the validation test set. Hyperparameter optimisation: number of training epochs = 1, learning rate = 4.9527941191800395e$-$05 and seed = 23 (for reproducibility)

**Fig. 10:**
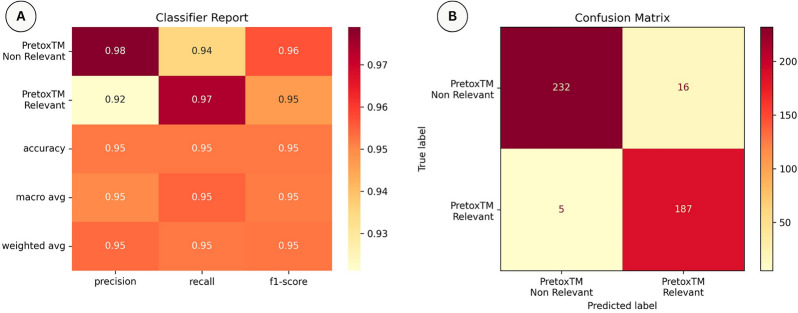
**A** Sentence classification performance resulting from fine-tuning the BioDistilBERT-uncased pre-trained model. Precision, recall and F1-score for each of the two categories: PretoxTM Relevant and Non-PretoxTM Relevant; followed by the average values accuracy, macro average and weighted average. **B** Confusion matrix of the two categories from the validation test set. Hyperparameter optimisation: number of training epochs = 7, learning rate = 2.0843088859954707e$-$05 and seed = 33 (for reproducibility)

**Fig. 11:**
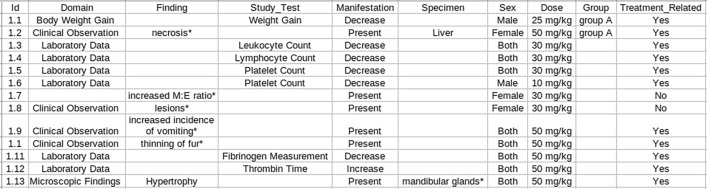
An example of a CSV file containing the exported abnormal observations is provided. This format is designed for easy comprehension by the user

**Fig. 12:**
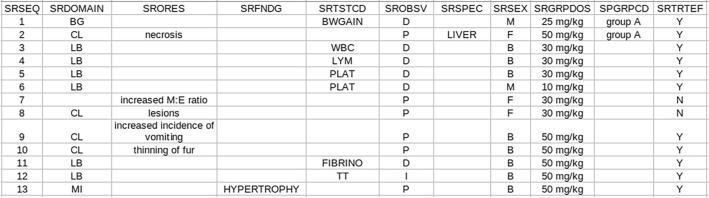
An example of a CSV file containing the exported abnormal observations in the SR-Domain template is provided. This format incorporates controlled terminology from SEND CDISC. This example displays a subset of the SR-Domain fields

## Data Availability

All the materials associated with the development of the PretoxTM project are openly available in several repositories listed on Table 6. All components of the PretoxTM project are freely accessible. The PretoxTM Corpus of treatment-related finding is completely free and public, and is intended to be used for future text mining tasks. The PretoxTM Corpus is licensed under Creative Commons Attribution-ShareAlike 4.0 International (CC BY-SA 4.0). Creative Commons licence allows users to share and adapt creative works, requiring attribution to the original creator and distribution under the same licence terms. As for the PretoxTM software, it is open source and can be accessed through the gitlab website. The PretoxTM Web Application and the PretoxTM Pipeline are licensed under the GNU General Public License Version 3 (GPLv3), as certain internal components of the pipeline utilise third-party libraries with this copyleft license. The NLP models for sentence classification and NER are licensed under the Apache License 2.0, allowing users to freely use, modify, and distribute the software for any purpose.

## Appendix A Study domains processed by PretoxTM

See Table 7.

**Table 7:** List of study domains covered by the PretoxTM system

Domain	Description
Body weight gain	Body weight gain is the actual difference between two body weight measurements for any given interval for a subject. This is most commonly shown as the difference between two consecutive body weight measurements.
Body weight	This domain captures body weights collected for subjects during the study and at the end of the study (terminal body weights).
Clinical observation	This domain captures clinical sign information including ophthalmology, physical examination, and dermal examination collected in life while executing the study.
Cardiovascular system findings	A findings domain that contains physiological and morphological findings related to the cardiovascular system, including the heart, blood vessels and lymphatic vessels.
Death Details; Death Diagnosis and Details	A findings domain that contains the diagnosis of the cause of death for a subject.
ECG test results	A findings domain that contains ECG data, including position of the subject, method of evaluation, all cycle measurements and all findings from the ECG including an overall interpretation if collected or derived.
Fetal measurements	The fetal measurements domain captures individual fetal body and tissue weights, as well as growth measurements.
Food and Water	This domain captures food/water consumption of animals in the study. The data in this domain is derived data.
Laboratory Test Results	A findings domain that contains laboratory test data such as hematology, clinical chemistry and urinalysis. This domain does not include microbiology or pharmacokinetic data, which are stored in separate domains.
Macroscopic Findings	The gross pathology findings recorded at necropsy.
Microscopic Findings	A findings domain that contains histopathology findings and microscopic evaluations.
Organ Measurements	Findings from organ measurement evaluations.
Respiratory System Findings	A findings domain that contains physiological and morphological findings related to the respiratory system, including the organs that are involved in breathing such as the nose, throat, larynx, trachea, bronchi and lungs.
Vital Signs	A findings domain that contains measurements including but not limited to blood pressure, temperature, respiration, body surface area, body mass index, height and weight.

## Appendix B Metrics obtained using different models for the PretoxTM sentence classification component

In here detailed information regarding the metrics obtained using different models for the sentence classification component (Figs. 8, 9, 10).

## Appendix C Metrics obtained using different models for the PretoxTM NER component

See Tables 8, 9, 10.

**Table 8:** NER performance resulting from fine-tuning the BiomedBERT pre-trained model

Entity	Precision	Recall	F1-score
Study Test	0.82	0.88	0.85
Manifestation	0.87	0.93	0.90
Finding	0.70	0.76	0.73
Specimen	0.75	0.78	0.76
Dose	0.86	0.88	0.88
Sex	0.88	0.96	0.92
Group	0.93	0.91	0.92
Overall	0.80	0.85	0.83

Hyperparameter optimisation: number of training epochs = 9, learning rate = 4.182448279085272e$-$05 and seed = 27 (for reproducibility)

**Table 9:** NER performance resulting from fine-tuning the Bio_ClinicalBERT pre-trained model

Entity	Precision	Recall	F1-score
Study Test	0.85	0.86	0.86
Manifestation	0.90	0.91	0.90
Finding	0.70	0.77	0.73
Specimen	0.77	0.83	0.80
Dose	0.90	0.92	0.91
Sex	0.87	0.95	0.91
Group	0.90	0.90	0.90
Overall	0.80	0.84	0.82

Hyperparameter optimisation: number of training epochs = 14, learning rate = 6.553248854264149e$-$05 and seed = 29 (for reproducibility)

**Table 10:** NER performance resulting from fine-tuning the BioDistilBERT-uncased pre-trained model

Entity	Precision	Recall	F1-score
Study Test	0.85	0.89	0.87
Manifestation	0.88	0.88	0.88
Finding	0.72	0.76	0.74
Specimen	0.75	0.81	0.78
Dose	0.88	0.90	0.90
Sex	0.91	0.91	0.91
Group	0.84	0.90	0.87
Overall	0.80	0.84	0.82

Hyperparameter optimisation: number of training epochs = 4, learning rate = 0.00013861257795383294 and seed = 17 (for reproducibility)

## Appendix D study report domain template

See Table 11.

**Table 11:** Study report domain template

Variable name	Variable label	Type	Controlled terms, codelist or format	Role	Notes	Core
STUDYID	Study Identifier	Char		Identifier	Unique identifier for a study within a SEND submission2.	Req
DOMAIN	Domain Abbreviation	Char	SR	Identifier	Two-character abbreviation for the domain	Req
SRSEQ	Sequence number	Num		Record Qualifier	Sequence number (starting at 1) to group findings that relate to the same condition for the same sex and treatment group.	Perm
SRRISK	Risk Level Associated with this Group/Sex	Char		Record Qualifier	A toxicity risk indicator. Can be one of: NOEL, LOEL, NOAEL, LOAEL, HNSTD, STD, MTD. This is a ‘must enter’ field for the NULL domain. Otherwise it is blank.	Perm
SPGRPCD	Sponsor-Defined Group Code	Char		Record Qualifier	Sponsor-defined identifier for the treatment group in which the finding was observed (e.g. “1”, “A”, etc.).	Req
GRPLBL	Group Name	Char		Record Qualifier	Sponsor-defined name of the treatment group (e.g. “Low Dose”, “Mid Dose”, etc.).	Req
SRGRPDOS	Group Dose Level	Char		Record Qualifier	Dose level of the treatment group (e.g. “10 mg/kg”, “10 ml/kg”, etc.)	Perm
SRSEX	Sex	Char		(SEX) + “B”	Sex of the animals in which the finding was observed. Standard extended to include ‘both’ sexes (“B”)	Req
SRSTDY	Study Day of Start of Finding	Num		Timing	Day on which the finding was first observed (can be blank)	Perm
SRSTPHSE	Study Phase of first Observation	Char	PRE-DOSING, DOSING, RECOVERY	Result Qualifier	Phase in which the observation was first made	Req
SROBSTDY	Start Phase Day of Observation	Num		Timing	Phase day of the first (or only) recorded occurrence of the observation (0 ->)	Req
SRENDY	Study Day of End of Finding	Num		Timing	Day on which the finding was last observed (can be blank)	Perm
SRENPHSE	Study Phase of last Observation	Char	PRE-DOSING, DOSING, RECOVERY	Result Qualifier	Phase in which the last [time-course] observation was made (can be blank)	Perm
SROBENDY	End Phase Day of Observation	Num		Timing	Phase day of the last recorded occurrence [time course] of an observation (can be blank)	Perm
SRDOMAIN	Domain of Finding	Char	(SDOMAIN) +<null>	Topic	Domain in which the finding was observed. Supported domains are: BW, BG, CL, CV, DD, EG, FW, LB, MA, MI, OM, PP, RE, TF, and VS. The NULL (blank) domain is supported for group- wide observations (see SRRISK). Out of scope domains are: PM, PC, all DART domains and all Trial Design domains.	Req
SRSPEC	Specimen of Finding	Char	(SPEC), (BODSYS)	Record Qualifier	Specimen in which the finding was observed. This is not required for all domains. For CL (Clinical Signs) domain, (BODSYS) is used instead of (SPEC).	Perm

Variable name	Variable label	Type	Controlled terms, codelist or format	Role	Notes	Core
SRTSTCD	Test Short Name	Char	(BGTESTCD), (BWTESTCD), (DDTESTCD), (EGTESTCD), (FWTESTCD), (LBTESTCD), (OMTESTCD), (PKPARMCD), (SCVTSTCD), (SRETSTCD) (TFTESTCD), (SVSTSTCD)	Record Qualifier	Short name of the measurement, test, or examination relating to the finding. The controlled term list is determined by the Domain in which the finding was observed. This field can only hold a controlled term. For some domains (e.g. CL, MA, MI, etc.) there is no associated test so this field will remain blank.	Perm
SRFNDG	Finding	Char	(NEOPLASM), (NONNEO), (CLCAT)	Record Qualifier	Finding in those cases where there is no associated test (such as for domains CL, MA, MI, etc.). This field can only hold a controlled term or is otherwise blank. For CL this field contains the finding ‘category’ (CLCAT).	Req
SRORES	Observation (original result)	Char		Result Qualifier	Contains the Finding as described in the associated study report.Req if SRFNDG is blank for MA and MI domains (because of SEND CT limitations). Otherwise Perm for all domains	Req/Perm
SROBSV	Manifestation of Finding	Char		Result Qualifier	“I” (Increase), “D” (Decrease), “P” (Present) or “A” (Absent)	Req
SROBSQ	Observation Qualifier	Char		Result Qualifier	“R” (Reversible), “T” (Transient) or blank.	Perm
SRSEV	Severity of Finding	Char	(SEV)	Variable Qualifier	Severity of the observation	Perm
SRPCNT	Scale of this Finding	Num		Result Qualifier	An estimate (%age) of the number of subjects in the treatment group that were affected by this severity (SRSEV) of finding. Can be blank (= 100%). Not valid when SRSEV is blank.	Perm
SRSIGF	Statistical Significance	Char		Variable Qualifier	Statistical significance of the observation: * = p< 0.1, ** = p< 0.01, *** = p< 0.001. Can be blank.	Perm
SRTRTEF	Treatment-Related?	Char	(NY)	Variable Qualifier	“Y” = finding is treatment-related; “N” = finding is not treatment- related; “U” = uncertain. Can be blank.	Req
SRCOMNT	Comment	Char		Variable Qualifier	Free text comment (can be blank).	Perm

## Appendix E Exported abnormal observations example

See Figs. 11, 12.

## References

[1] Wouters OJ, McKee M, Luyten J (2020) Estimated research and development investment needed to bring a new medicine to market, 2009–2018. JAMA 323(9):844–853. 10.1001/jama.2020.1166 32125404 10.1001/jama.2020.1166 PMC7054832

[2] Qureshi R, Irfan M, Gondal TM, Khan S, Wu J, Hadi MU, Heymach J, Le X, Yan H, Alam T (2023) AI in drug discovery and its clinical relevance. Heliyon. 10.1016/j.heliyon.2023.e17575 37396052 10.1016/j.heliyon.2023.e17575 PMC10302550

[3] Pognan F, Steger-Hartmann T, Díaz C, Blomberg N, Bringezu F, Briggs K, Callegaro G, Capella-Gutierrez S, Centeno E, Corvi J, Drew P, Drewe WC, Fernández JM, Furlong LI, Guney E, Kors JA, Mayer MA, Pastor M, Piñero J, Ramírez-anguita JM, Ronzano F, Rowell P, Saüch-pitarch J, Valencia A, Water B, Lei J, Mulligen E, Sanz F (2021) The eTRANSAFE project on translational safety assessment through integrative knowledge management: achievements and perspectives. Pharmaceuticals. 10.3390/ph14030237 33800393 10.3390/ph14030237 PMC7999019

[4] Sanz F, Pognan F, Steger-Hartmann T, Diaz C, Asakura S, Amberg A, Bécourt-Lhote N, Blomberg N, Bosc N, Briggs K, Bringezu F, Brulle-Wohlhueter C, Brunak S, Bueters R, Callegaro G, Capella-Gutierrez S, Centeno E, Corvi J, Cronin M, Wilkinson D (2023) eTRANSAFE: data science to empower translational safety assessment. Nat Rev Drug Discovery. 10.1038/d41573-023-00099-5 37316648 10.1038/d41573-023-00099-5

[5] Briggs K, Bosc N, Camara T, Diaz C, Drew P, Drewe WC, Kors JA, Mulligen EV, Pastor Maeso M, Pognan F (2021) Guidelines for FAIR sharing of preclinical safety and off-target pharmacology data. ALTEX. 10.14573/altex.2011181 33637997 10.14573/altex.2011181

[6] Pastor M, Gómez-Tamayo JC, Sanz F (2021) Flame: an open source framework for model development, hosting, and usage in production environments. J Cheminf 13(1):31. 10.1186/s13321-021-00509-z 10.1186/s13321-021-00509-z PMC8054391 33875019

[7] Piñero J, Fraga PSR, Valls-Margarit J, Ronzano F, Accuosto P, Jane RL, Sanz F, Furlong LI (2023) Genomic and proteomic biomarker landscape in clinical trials. Comput Struct Biotechnol J 21:2110–2118. 10.1016/j.csbj.2023.03.014 36968019 10.1016/j.csbj.2023.03.014 PMC10036891

[8] Callegaro G, Kunnen S, Trairatphisan P, Grosdidier S, Niemeijer M, Den Hollander W, Guney E, Piñero J, Furlong LI, Webster Y, Saez-Rodriguez J, Sutherland J, Mollon J, Stevens J, Water B (2021) The human hepatocyte TXG-MAPr: gene co-expression network modules to support mechanism-based risk assessment. Arch Toxicol 95:1–31. 10.1007/s00204-021-03141-w 34626214 10.1007/s00204-021-03141-w PMC8536636

[9] CDISC: SEND Controlled Terminology. Accessed: 2024-07-04. https://evs.nci.nih.gov/ftp1/CDISC/SEND/

[10] Choudhary S, Walker A, Funk K, Keenan C, Khan I, Maratea K (2018) The standard for the exchange of nonclinical data (SEND): challenges and promises. Toxicol Pathol 46(8):1006–1012. 10.1177/0192623318805743 30295163 10.1177/0192623318805743

[11] Souza T, Kush R, Evans JP (2007) Global clinical data interchange standards are here! Drug Discovery Today 12(3):174–181. 10.1016/j.drudis.2006.12.012 17275739 10.1016/j.drudis.2006.12.012

[12] Sanz F, Pognan F, Steger-Hartmann T, Díaz C, Cases M, Pastor M, Marc P, Wichard J, Briggs K, Watson DK, Kleinöder T, Yang C, Amberg A, Beaumont M, Brookes AJ, Brunak S, Cronin MTD, Ecker GF, Escher S, Greene N, Guzmán A, Hersey A, Jacques P, Lammens L, Mestres J, Muster W, Northeved H, Pinches M, Saiz J, Sajot N, Valencia A, Lei J, Vermeulen NPE, Vock E, Wolber G, Zamora I (2017) eTOX: Legacy data sharing to improve drug safety assessment: the eTOX project. Nat Rev Drug Discovery 16(12):811–812. 10.1038/nrd.2017.177 29026211 10.1038/nrd.2017.177

[13] Ravagli C, Pognan F, Marc P (2016) OntoBrowser: a collaborative tool for curation of ontologies by subject matter experts. Bioinformatics 33(1):148–149. 10.1093/bioinformatics/btw579 27605099 10.1093/bioinformatics/btw579 PMC5408772

[14] Vaswani A, Shazeer N, Parmar N, Uszkoreit J, Jones L, Gomez AN, Kaiser L, Polosukhin I (2017) Attention Is All You Need. In: Proceedings of the 31st International Conference on Neural Information Processing Systems. NIPS’17, pp. 6000–6010. Curran Associates Inc., Red Hook, NY, USA. 10.48550/arXiv.1706.03762

[15] Devlin J, Chang M-W, Lee K, Toutanova K (2019) BERT: Pre-training of Deep Bidirectional Transformers for Language Understanding. In: Proceedings of the 2019 Conference of the North American Chapter of the Association for Computational Linguistics: Human Language Technologies, Volume 1 (Long and Short Papers), pp. 4171–4186. Association for Computational Linguistics, Minneapolis, Minnesota. 10.18653/v1/N19-1423

[16] OpenAI et al (2024) GPT-4 Technical Report. 10.48550/arXiv.2303.08774

[17] Lee J, Yoon W, Kim S, Kim D, Kim S, So CH, Kang J (2020) BioBERT: a pre-trained biomedical language representation model for biomedical text mining. Bioinformatics 36(4):1234–1240. 10.1093/bioinformatics/btz682 31501885 10.1093/bioinformatics/btz682 PMC7703786

[18] Michalopoulos G, Wang Y, Kaka H, Chen H, Wong A (2021) UmlsBERT: Clinical Domain Knowledge Augmentation of Contextual Embeddings Using the Unified Medical Language System Metathesaurus. In: Proceedings of the 2021 Conference of the North American Chapter of the Association for Computational Linguistics: Human Language Technologies, pp. 1744–1753. Association for Computational Linguistics, Online. 10.18653/v1/2021.naacl-main.139

[19] Alsentzer E, Murphy J, Boag W, Weng W-H, Jindi D, Naumann T, McDermott M (2019) Publicly Available Clinical BERT Embeddings. In: Proceedings of the 2nd Clinical Natural Language Processing Workshop, pp. 72–78. Association for Computational Linguistics, Minneapolis, Minnesota, USA. 10.18653/v1/W19-1909

[20] Rohanian O, Nouriborji M, Kouchaki S, Clifton DA (2023) On the effectiveness of compact biomedical transformers. Bioinformatics 39(3):103. 10.1093/bioinformatics/btad103 10.1093/bioinformatics/btad103 PMC10027428 36825820

[21] Luo R, Sun L, Xia Y, Qin T, Zhang S, Poon H, Liu T-Y (2022) BioGPT: generative pre-trained transformer for biomedical text generation and mining. Brief Bioinform. 10.1093/bib/bbac409 36156661 10.1093/bib/bbac409

[22] Gu Y, Tinn R, Cheng H, Lucas M, Usuyama N, Liu X, Naumann T, Gao J, Poon H (2020) Domain-specific language model pretraining for biomedical natural language processing. 10.1145/3458754

[23] Zhang Y, Liu C, Liu M, Liu T, Lin H, Huang C-B, Ning L (2024) Attention is all you need: utilizing attention in AI-enabled drug discovery. Brief Bioinform 25(1):467. 10.1093/bib/bbad467 10.1093/bib/bbad467 PMC10772984 38189543

[24] Liu Z, Roberts RA, Lal-Nag M, Chen X, Huang R, Tong W (2021) AI-based language models powering drug discovery and development. Drug Discovery Today 26(11):2593–2607. 10.1016/j.drudis.2021.06.009 34216835 10.1016/j.drudis.2021.06.009 PMC8604259

[25] Piñero J, Ramírez-Anguita JM, Saüch-Pitarch J, Ronzano F, Centeno E, Sanz F, Furlong LI (2019) The DisGeNET knowledge platform for disease genomics: 2019 update. Nucleic Acids Res 48(D1):845–855. 10.1093/nar/gkz1021 10.1093/nar/gkz1021 PMC7145631 31680165

[26] Thompson P, Daikou S, Ueno K, Batista-Navarro R, Tsujii J, Ananiadou S (2018) Annotation and detection of drug effects in text for pharmacovigilance. J Cheminf 10(1):37. 10.1186/s13321-018-0290-y 10.1186/s13321-018-0290-y PMC6089860 30105604

[27] Murphy RM, Klopotowska JE, Keizer NF, Jager KJ, Leopold JH, Dongelmans DA, Abu-Hanna A, Schut MC (2023) Adverse drug event detection using natural language processing: a scoping review of supervised learning methods. PLoS ONE 18(1):0279842. 10.1371/journal.pone.0279842 10.1371/journal.pone.0279842 PMC9810201 36595517

[28] Bollegala D, Maskell S, Sloane R, Hajne J, Pirmohamed M (2018) Causality patterns for detecting adverse drug reactions from social media: text mining approach. JMIR Public Health Surveill 4(2):8214. 10.2196/publichealth.8214 10.2196/publichealth.8214 PMC5966656 29743155

[29] Wasylewicz ATM, Burgt B, Weterings A, Jessurun N, Korsten E, Egberts T, Bouwman A, Kerskes M, Grouls R, Linden C (2021) Identifying adverse drug reactions from free- text electronic hospital health record notes. Br J Clin Pharmacol. 10.1111/bcp.15068 34468999 10.1111/bcp.15068 PMC9292762

[30] Narayanan S, Mannam K, Achan P, Ramesh MV, Rangan PV, Rajan SP (2022) A contextual multi-task neural approach to medication and adverse events identification from clinical text. J Biomed Inf 125:103960. 10.1016/j.jbi.2021.103960 10.1016/j.jbi.2021.103960 34875387

[31] Corradi MPF, de Haan AM, Staumont B, Piersma AH, Geris L, Pieters RHH, Krul CAM, Teunis MAT (2022) Natural language processing in toxicology: delineating adverse outcome pathways and guiding the application of new approach methodologies. Biomater Biosyst 7:100061. 10.1016/j.bbiosy.2022.100061 36824484 10.1016/j.bbiosy.2022.100061 PMC9934466

[32] PDS Consultants: SR-Domain template and concept. Copyright notice (2024)

[33] Neves M, Ševa J (2021) An extensive review of tools for manual annotation of documents. Brief Bioinform. 10.1093/bib/bbz130 31838514 10.1093/bib/bbz130 PMC7820865

[34] Yimam SM, Gurevych I, Castilho R, Biemann C (2013) WebAnno: a flexible, web-based and visually supported system for distributed annotations. In: Proceedings of the 51st Annual Meeting of the Association for Computational Linguistics: System Demonstrations, pp. 1–6. Association for Computational Linguistics, Sofia, Bulgaria. https://www.aclweb.org/anthology/P13-4001

[35] Artstein R, Poesio M (2008) Survey article: inter-coder agreement for computational linguistics. Comput Linguist 34(4):555–596. 10.1162/coli.07-034-R2

[36] Castro S (2017) Fast Krippendorff: fast computation of Krippendorff’s alpha agreement measure. GitHub

[37] GROBID. GitHub. Accessed: 2024-07-18 (2008–2024). https://github.com/kermitt2/grobid

[38] Smith R (2007) An overview of the tesseract OCR engine. In: Ninth International Conference on Document Analysis and Recognition (ICDAR 2007), vol. 2, pp. 629–633. 10.1109/ICDAR.2007.4376991

[39] Patel C, Patel A, Patel D (2012) Optical character recognition by open source OCR tool tesseract: a case study. Int J Comput Appl 55(10):50–56. 10.5120/8794-2784

[40] Sporici D, Cusnir E, Boiangiu C-A (2020) Improving the accuracy of tesseract 4.0 ocr engine using convolution-based preprocessing. Symmetry. 10.3390/sym12050715

[41] Brisinello M, Grbić R, Pul M, Anđelić T (2017) Improving optical character recognition performance for low quality images. In: 2017 International Symposium ELMAR, pp. 167–171. 10.23919/ELMAR.2017.8124460

[42] Manning C, Surdeanu M, Bauer J, Finkel J, Bethard S, McClosky D (2014) The Stanford CoreNLP Natural Language Processing Toolkit. In: Proceedings of 52nd Annual Meeting of the Association for Computational Linguistics: System Demonstrations, pp. 55–60. Association for Computational Linguistics, Baltimore, Maryland. 10.3115/v1/P14-5010

[43] Cunningham H, Tablan V, Roberts A, Bontcheva K (2013) Getting more out of biomedical documents with GATE’s full lifecycle open source text analytics. PLoS Comput Biol. 10.1371/journal.pcbi.1002854 23408875 10.1371/journal.pcbi.1002854 PMC3567135

[44] Chalkidis I, Fergadiotis M, Malakasiotis P, Aletras N, Androutsopoulos I (2020) LEGAL-BERT: the Muppets straight out of Law School. In: Cohn T, He Y, Liu Y (eds.) Findings of the Association for Computational Linguistics: EMNLP 2020, pp. 2898–2904. Association for Computational Linguistics, Online. 10.18653/v1/2020.findings-emnlp.261

[45] Araci D (2019) FinBERT: financial sentiment analysis with pre-trained language models. CoRR abs/1908.10063[SPACE]10.48550/arXiv.1908.10063

[46] Chithrananda S, Grand G, Ramsundar B (2020) ChemBERTa: Large-Scale Self-Supervised Pretraining for Molecular Property Prediction. 10.48550/arXiv.2010.09885

[47] Loshchilov I, Hutter F (2019) Decoupled weight decay regularization. 10.48550/arXiv.1711.05101

[48] Bodenreider O (2004) The Unified Medical Language System (UMLS): integrating biomedical terminolog. Nucleic Acids Res 32(suppl–1):267–270. 10.1093/nar/gkh061 10.1093/nar/gkh061 PMC308795 14681409

[49] Cunningham H, Maynard D, Tablan V (2000) JAPE: a Java Annotation Patterns Engine. https://api.semanticscholar.org/CorpusID:59651445

[50] Stenetorp P, Pyysalo S, Topić G, Ohta T, Ananiadou S, Tsujii J (2012) brat: a Web-based Tool for NLP-Assisted Text Annotation. In: Proceedings of the Demonstrations at the 13th Conference of the European Chapter of the Association for Computational Linguistics, pp. 102–107. Association for Computational Linguistics, Avignon, France. https://aclanthology.org/E12-2021

[51] Kumar V, Choudhary A, Cho E (2020) Data Augmentation using Pre-trained Transformer Models. In: Campbell WM, Waibel A, Hakkani-Tur D, Hazen TJ, Kilgour K, Cho E, Kumar V, Glaude H (eds.) Proceedings of the 2nd Workshop on Life-long Learning for Spoken Language Systems, pp. 18–26. Association for Computational Linguistics, Suzhou, China. https://aclanthology.org/2020.lifelongnlp-1.3

[52] Edwards A, Ushio A, Camacho-collados J, Ribaupierre H, Preece A (2022) Guiding Generative Language Models for Data Augmentation in Few-Shot Text Classification. In: Dragut E, Li Y, Popa L, Vucetic S, Srivastava S (eds.) Proceedings of the Fourth Workshop on Data Science with Human-in-the-Loop (Language Advances), pp. 51–63. Association for Computational Linguistics, Abu Dhabi, United Arab Emirates (Hybrid). https://aclanthology.org/2022.dash-1.8/

[53] Cai J, Huang S, Jiang Y, Tan Z, Xie P, Tu K (2023) Improving Low-resource Named Entity Recognition with Graph Propagated Data Augmentation. In: Rogers A, Boyd-Graber J, Okazaki N (eds.) Proceedings of the 61st Annual Meeting of the Association for Computational Linguistics (Volume 2: Short Papers), pp. 110–118. Association for Computational Linguistics, Toronto, Canada. 10.18653/v1/2023.acl-short.11

[54] Zhou R, Li X, He R, Bing L, Cambria E, Si L, Miao C (2022) MELM: data augmentation with masked entity language modeling for low-resource NER. In: Muresan S, Nakov P, Villavicencio A (eds.) Proceedings of the 60th Annual Meeting of the Association for Computational Linguistics (Volume 1: Long Papers), pp. 2251–2262. Association for Computational Linguistics, Dublin, Ireland. 10.18653/v1/2022.acl-long.160

[55] Tian S, Jin Q, Yeganova L, Lai P-T, Zhu Q, Chen X, Yang Y, Chen Q, Kim W, Comeau DC, Islamaj R, Kapoor A, Gao X, Lu Z (2024) Opportunities and challenges for ChatGPT and large language models in biomedicine and health. Brief Bioinform 25(1):493. 10.1093/bib/bbad493 10.1093/bib/bbad493 PMC10762511 38168838

[56] Chen Q, Hu Y, Peng X, Xie Q, Jin Q, Gilson A, Singer MB, Ai X, Lai P-T, Wang Z, Keloth VK, Raja K, Huang J, He H, Lin F, Du J, Zhang R, Zheng WJ, Adelman RA, Lu Z, Xu H (2024) A systematic evaluation of large language models for biomedical natural language processing: benchmarks, baselines, and recommendations. https://arxiv.org/abs/2305.16326 10.1038/s41467-025-56989-2 PMC11972378 40188094

[57] Steger-Hartmann T, Kreuchwig A, Vaas L, Wichard J, Bringezu F, Amberg A, Muster W, Pognan F, Barber C (2020) Introducing the concept of virtual control groups into preclinical toxicology testing. ALTEX 37(3):343–349. 10.14573/altex.2001311 32242633 10.14573/altex.2001311

[58] Golden E, Allen D, Amberg A, Anger LT, Baker E, Baran SW, Bringezu F, Clark M, Duchateau-Nguyen G, Escher SE et al (2024) Toward implementing virtual control groups in nonclinical safety studies: workshop report and roadmap to implementation. ALTEX 41(2):282–301. 10.14573/altex.2310041 38043132 10.14573/altex.2310041

[59] Vlahou A, Hallinan D, Apweiler R, Argiles A, Beige J, Benigni A, Bischoff R, Black PC, Boehm F, Céraline J, Chrousos GP, Delles C, Evenepoel P, Fridolin I, Glorieux G, Gool AJ, Heidegger I, Ioannidis JPA, Jankowski J, Jankowski V, Jeronimo C, Kamat AM, Masereeuw R, Mayer G, Mischak H, Ortiz A, Remuzzi G, Rossing P, Schanstra JP, Schmitz-Dräger BJ, Spasovski G, Staessen JA, Stamatialis D, Stenvinkel P, Wanner C, Williams SB, Zannad F, Zoccali C, Vanholder R (2021) Data sharing under the general data protection regulation. Hypertension 77(4):1029–1035. 10.1161/hypertensionaha.120.16340 33583200 10.1161/HYPERTENSIONAHA.120.16340 PMC7968961

[60] Lappalainen I, Almeida-King J, Kumanduri V, Senf A, Spalding JD, Ur-Rehman S, Saunders G, Kandasamy J, Caccamo M, Leinonen R, Vaughan B, Laurent T, Rowland F, Marin-Garcia P, Barker J, Jokinen P, Torres AC, Argila JR, Llobet OM, Medina I, Puy MS, Alberich M, Torre S, Navarro A, Paschall J, Flicek P (2015) The European Genome-phenome Archive of human data consented for biomedical research. Nat Genet 47(7):692–695. 10.1038/ng.3312 26111507 10.1038/ng.3312 PMC5426533

