# Supplementary material for: PretoxTM: a text mining system for extracting treatment-related findings from preclinical toxicology reports
Source: J Cheminform. 2025 Feb 3;17:15. doi: 10.1186/s13321-024-00925-x (PMC11792311; doi:10.1186/s13321-024-00925-x)
Supplement: Supplementary file 1 — Additional file 1. [file 13321_2024_925_MOESM1_ESM.pdf]

777365 – eTRANSAFE

Enhancing TRANslational SAFEty Assessment through Integrative Knowledge Management

Text-Mining Annotation Guideline: Treatment-related findings

**Authors**	Javier Corvi (BSC), Emilio Centeno (IMIM), Francesco Ronzano (IMIM)
**Contributors**	Laura Furlong (IMIM), Salvador Capella-Gutierrez (BSC), José María Fernández (BSC)

DOCUMENT HISTORY **2**

1. INTRODUCTION **3**

2. THE ANNOTATION SCHEMA **3**
2.1. FINDING 4
2.2. COMPOSE DOSE or GROUP (CDoG) 6
2.3. Relations 7
2.3.1 CDoG → FINDING (CDoG_finding) 7
2.3.2 Discontinued Expressions (disc_expression) 8

3. THE ANNOTATION ENVIRONMENT AND PRELOADED DOCUMENTS **9**
3.1. WEBANNO: THE ANNOTATION TOOL 10
3.2. THE CORPUS 12

4. ANNOTATION EXAMPLES **13**
4.1. Treatment-related finding annotation with WebAnno 13

5. FREQUENTLY ASKED QUESTIONS **18**
5.1. 1st follow up meeting 18

DOCUMENT HISTORY

Version	Date	Description
1	04/10/2019	First version of the annotation schema, used during the 1st eTRANSAFE Text-Mining annotation workshop.
2	03/01/2020	Modification of the annotation schema. After the workshop's feedback, the annotation schema was reviewed and changed with a new approach that simplifies and speeds up the annotation process.
2.1	04/05/2020	Added section 5 frequently asked questions from the first annotation activity week.

1. INTRODUCTION

This document provides detailed guidelines for the annotation of treatment-related findings (TRFs) in toxicology reports shared by EFPIA partners in the context of the eTRANSAFE project. These guidelines can be used to generate a corpus of relevant sections of preclinical toxicology studies where textual references to TRFs are manually annotated. This corpus will be exploited to train and validate automated approaches to identify textual references to TRFs in toxicology reports.

The 1st eTRANSAFE Text-Mining annotation workshop took place on October 15th 2019; during the workshop the first version of the annotation guideline was introduced. After the corpus annotation activity, a discussion slot was organized in order to obtain feedback from the EFPIA partners. Several issues were detected regarding the complexity of the annotation process. This 2nd version of the annotation guidelines try to solve these issues with a different approach that will be addressed in this document.

Additional materials (documentation, videos, etc.) can be found at:
https://etransafe.bsc.es/nextcloud/index.php/s/jW2qdfnSPjNAXXy

2. THE ANNOTATION SCHEMA

The annotation process consists in spotting, inside the textual contents of a toxicology report, the presence of treatment related findings (TRFs). A TRF is an observation of a treatment-associated adverse effect that can be characterized by several features like the dose administered, the sex of the animals, etc. However, the manual annotation of many types of concepts and relations can overcomplicate the annotation process, hindering the task of establishing a consensus annotation. In order to simplify the manual annotation process, the annotation schema has been reduced to a simple schema, with just two types of entities: the treatments (the doses administered) and the findings.

The annotation schema (Figure 1) consists of two types of concept to be detected in the text:

ADVERSE_OBSERVATION: a broad concept/expression that captures the whole abnormal observation.

Compound Dose or Group (CDoG): Dose of the compound, or group of animals with an associated dose.

Important Note: The term "**ADVERSE_OBSERVATION**" expression has replaced the old term "FINDING" expression. However, to maintain consistency throughout the document, the old term "FINDING" expression is used.

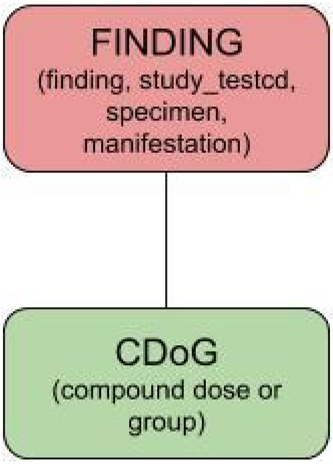

Figure 1. Annotation Schema. The FINDING and the Compound Dose or Group; two types of expression to be labeled in the text.

2.1. FINDING

A FINDING (from now on in uppercase to refer to the annotation schema entity) entity is defined by the minimum expression that captures the abnormal or relevant observation. This broad concept now covers several SR-Domain facets, like SRFNDNG (finding), SROBSV (manifestation of finding), SRTSTCD (test short name) and SRSPEC (specimen).

Some examples of FINDINGs (in **red** color):

"... Females receiving 2500 or 5000 mg/kg/day gained slightly less body weight than the control group (p<0.05 and p<0.01 respectively for Week 0 to 13 gain) during the course of the study..."

"... Pale faeces were noted under the cages of males and females receiving 2500 or 5000 mg/kg/day (Groups 4 and 5) from Week 6..."

"... The slight increase in serum calcium at the highest dose of 500 µg/kg COMPOUND_XXX in male animals on days 10…."

To annotate a FINDING expression these rules have to be taken into account:

- The FINDING expression should include the action.

  "Sporadic soft stool and absence of feces were also observed in some animals at 25 or 50 mg/kg."

- It is allowed to mark a coordination or enumeration of FINDINGs as a single one, as far as they are associated to the same dose/s. This is a convenience for the sake of simplicity, so in case of doubt, FINDINGs should be annotated individually.

  "Sporadic soft stool and absence of feces were also observed in some animals at 25 or 50 mg/kg."

- When several parts of FINDING expressions are coordinated they also can be marked as a single FINDING, as far as they are associated to the same dose/s,

  "The high dose of 375 µmol Gd/kg bw. led to marked clinical findings and further affected kidneys, liver, reproductive, hematopoietic and immunologic organs as primary targets of toxicity."

- Sometimes FINDINGs are described from a more general observation to more specific FINDINGs. In this case, when the FINDINGs are located in a contiguous region of text, it is allowed to mark the whole piece of text as a single FINDING. Again, in case of doubt, FINDINGs should be annotated individually.

  "... The corticomedullary region and the pelvis also show reactive changes to the mineralization in form of tubular simple dilatation, tubular regeneration, inflammation and tubular vacuolation ..."

  "The decrease in food consumption and body weight of the animals from the mid dose onwards is regarded as evidence of general toxicity of COMPOUND_XXX."

- If there is information regarding the manifestation of the finding in parenthesis, it has to be included in the FINDING.

  "Light yellow discoloration of the serum and xiphoid cartilage, treatment-related nephropathy (slight) in one rat, and centrilobular hepatocellular hypertrophy (minimal)"

- All the findings has to be included, even the incidental ones:

  "Regarding clinical chemistry parameters, the increased GLDH observed only in one 30 mg/kg female was considered incidental."

- When the animal of study dies or were killed because of the treatment, has to be labeled:

  "Four males and 10 females given 400 mg/kg/day died or were euthanized within 7 to 22 days after initiation of treatment."

2.2. COMPOSE DOSE or GROUP (CDoG)

The treatments are represented by CDoG entities. A CDoG describes a **dose** of the compound administered to a group of animals.

"Administration of COMPOUND_XXX 1 for 4 weeks in Wistar rats induced the premature kill of 7/15 females given 40 mg/kg …".

The dose may be implicitly mentioned by referencing a dosing group or dosing level. In that case, the group or dosing level expression must be annotated as CDoG. In summary, the minimum information to identify the dose for which a FINDING is observed must be annotated as CDoG. In the following examples the CDoGs are marked in **yellow**.

"... At dose level 3, absolute and relative liver weights were increased in male rats."

"In group III, the heart rates were increased in both sexes in a week."

The following rules and examples describe how to annotate CDoGs:

- For explicit doses, the units must be included within the annotation. Note that the number and the sex of the animals affected are not included in the finding or in the group.

  "Administration of COMPOUND_XXX 1 for 4 weeks in Wistar rats induced the premature kill of 7/15 females given 40 mg/kg …".

- Qualitative expressions used to reinforce the dosing information must be included within the annotation.

  "The high dose of 375 µmol Gd/kg bw. led to marked clinical findings and further affected kidneys, liver, reproductive, hematopoietic and immunologic organs as primary targets of toxicity."

  "... Pale faeces were noted under the cages of males and females receiving 2500 or 5000 mg/kg/day (Groups 4 and 5) from Week 6...".

  ".... increase in alkaline phosphatase on day 28, which lasted until day 49 in animals of the high dose (group 6) of 50 mg/kg….."

- The sex is not part of the CDoGs.

  "... feed intake was slightly reduced in group III-males in comparison to control…"

- When a dose description contains information that implies that the effect is from that doses on, the terms has to be included:

  "From the mid dose (75 µmol Gd/kg bw.) on, laboratory examinations indicated anemia with increased reticulocytes ...."

2.3. Relations

In an annotation schema, relations are used to describe the types of association between two entities. In our case, we have a primary relation to connect a treatment (CDoG) with a FINDING (CDoG → FINDING). Be aware of the directionality, only from CDoG to FINDING is allowed, but **not** the way around. We also define another type of relationship to connect not-strictly-contiguous parts of the same entity. It is therefore a convenient relation used to connect the parts of a discontinued expression rather than connecting different entities.

2.3.1 CDoG → FINDING (CDoG_finding)

The CDoG_finding relation associates a CDoG to a FINDING.

CDoG_finding

"The high dose of 375 µmol Gd/kg bw. led to marked clinical findings and further affected kidneys, liver, reproductive, hematopoietic and immunologic organs as primary targets of toxicity."

In this example, the same CDoG is associated to two different FINDINGs:

"At the clinical chemistry assessment, in females group,

CDoG_finding CDoG_finding

total bilirubin concentration and aspartate aminotransferase activity were found to be reduced by 44 and 15% respectively, whilst total protein and cholesterol concentrations were increased by 6 and 31% respectively."

The following rules and examples describe how to annotate CDoG → FINDING (CDoG_finding):

- The entities could appear in different sentences:

disc_expression (explained in the next section)

"From 35 mg/kg COMPOUND_XXX onwards:

CDoG_finding

-single or repeated vomitus over several days with dose-dependently increasing frequency and incidence in two animals of group 2 and all animals of groups 3 and 4.

CDoG_finding

-slight decrease in systolic blood pressure in individual animals of all groups."

- The same finding related to different dosis, and also be clear that the sex is not part of the CDoG:

CDoG_finding CDoG_finding

"…. decreases in reticulocytes, observed in 30 mg/kg males and in 10 mg/kg females, and the …"

2.3.2 Discontinued Expressions (disc_expression)

A limitation in the annotation of an entity is that it must be contiguous in the text. However, FINDINGs and CDoGs expressions may not be fully contiguous in the text: i.e. there is another expression, with another type of meaning, in the middle of the FINDING or CDoG expression. In order to overcome this limitation, the disc_exression relation has been created: it allows to connect discontinued expressions of a same entity (FINDING or CDoG).

In the following example:

"The corticomedullary region and the pelvis also show, after 15 days of the beginning of the treatment, tubular simple

disc_expression

dilatation, tubular regeneration, inflammation and tubular vacuolation."

The expression *", after 15 days of the beginning of the treatment,"* is not part of the FINDING. I.e: it is not needed to specify the FINDING, it gives another type of information.

To connect a discontinued expression to another entity, you just have to connect any part (not all of them) of the discontinued expression to the entity of interest. In the following example, the FINDING is a discontinued expression that must be connected to a CDoG expression. It is enough just to connect one of the parts of the discontinued expression of the FINDING to the CDoG. I.e.: we do not have to connect each part of the FINDING to the CDoG.

A discontinued CDoG example, that relates "*low dose of 0.2 mg/kg*" with "*and higher*":

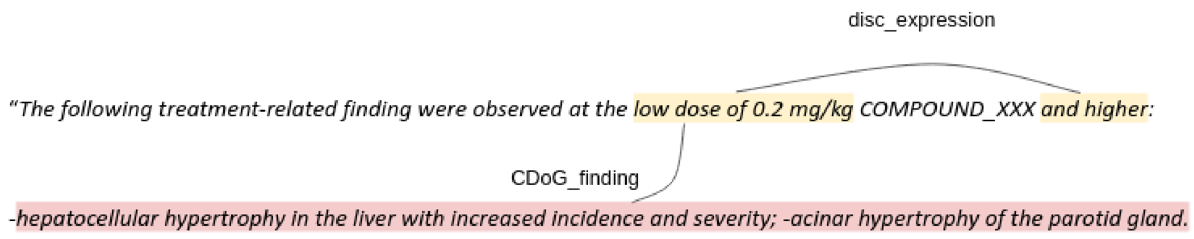

3. THE ANNOTATION ENVIRONMENT AND PRELOADED DOCUMENTS

In order to ease the annotation task, each annotator will be given access to a Web based annotation framework (i.e. WebAnno, see Section 3.1) where, by relying on a Web browser, she/he will be able to annotate a collection of text excerpts extracted from toxicology reports, referred to as the *eTRANSAFE preclinical toxicology corpus*.

Section 3.2 describes the procedure followed to extract the text excerpts from the toxicology reports that will be used to develop the corpus.

3.1. WEBANNO: THE ANNOTATION TOOL

WebAnno is the annotation tool that will be used during the Text Mining Annotation activity. WebAnno is a web-based annotation tool for a wide range of linguistic annotations including various layers of morphological, syntactical, and semantic annotations. A WebAnno server is installed at the BSC facilities and can be accessed through: https://etransafe.bsc.es/webanno/.

This section provides a general overview of WebAnno, useful to get acquainted with the main interaction patterns and configuration options of this tool. In section 4, examples of TRFs annotations are explained. Remember there is also available at https://etransafe.bsc.es/nextcloud/index.php/s/jW2qdfnSPjNAXXy the "Video Tutorial 1. Introduction to basic navigation in WebAnno".

Login and Home Page

After logging-in with your username and password, the homepage, Figure 1, with the "Annotation" link will appear.

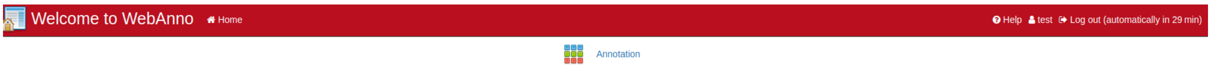

Figure 1. User homepage overview.

Open a document

Press the “Annotation” menu option, select the Project and then select the document.

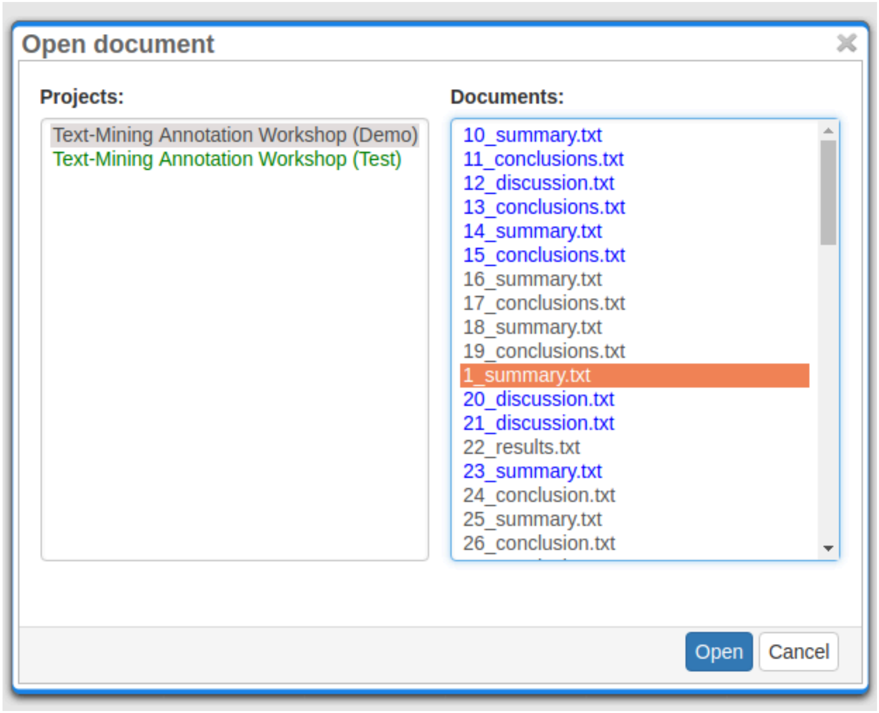

Figure 2. Open a specific document.

Document Overview

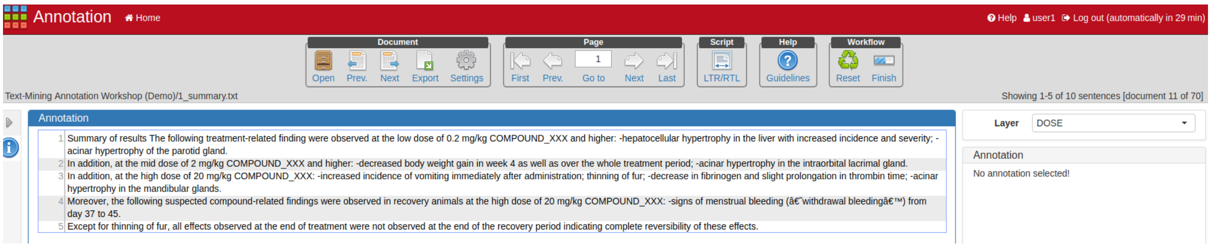

Figure 3. Document overview.

In Figure 3, several options in the central tab of the document can be appreciated. The most important are the Page navigation and Workflow options. It’s important to remark that by

default only five sentences are shown; in this example, the document has a total of 10 sentences, this can be observed at the right of the screen in Figure 3 (“Showing 1-5 of 10 sentences …. ”). Take this into account in order to annotate all the document and do not forget sentences included in other pages.

The Workflow options refer to the state of the annotation process, once the annotation of the document is complete, you have to finish the annotation process using the “Finish” button (do not press this button if you have not finished yet). The reset button will erase all your annotations, thus use it with caution.

The layout of the page and the several facets concerning the visualization of contents can be changed with the “Settings” option. For example, you can change the number of visible sentences or the font size of the content as shown in Figure 4.

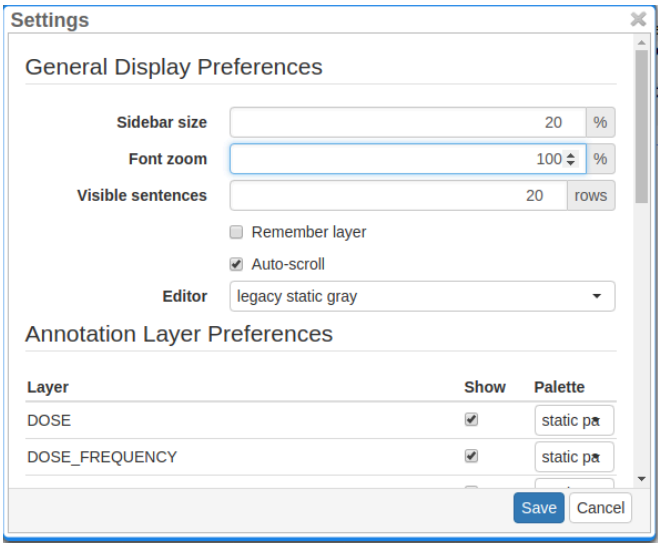

Figure 4. Setting options overview.

These are the basic navigation options of WebAnno. Section 4 describes annotation examples using this tool.

3.2. THE CORPUS

In order to ease the annotation process, relevant sections of toxicology reports, e.g. “results”/”conclusion” have been identified in the initial set of documents considered for annotation and included in the *eTRANSAFE preclinical toxicology corpus.* In particular, such relevant sections were extracted from toxicological reports shared by Company A (87), Company B (29), Company C (16), Company D (11), Company E (8) and Company F (5). The corpus in its current state, includes 129 relevant sections with a total of 2511 sentences. The relevant sections included during the annotation activity could increase with time.

Toxicology reports are very exhaustive and comprehensive, reporting every aspect of each experiment. According to the results of a survey to the EFPIA partners, most TRFs are located in the “summary”, “conclusions” and, sometimes, “results” sections. Moreover, toxicology reports are structured heterogeneously, even when we consider distinct reports provided by the same EFPIA partner, i.e. there may be different types of sections and sections may be named differently from a document to another. In order to reduce the complexity and avoid potential false positives, we have manually selected (automatically in the future) relevant sections giving rise to reduced documents.

4. ANNOTATION EXAMPLES

4.1. Treatment-related finding annotation with WebAnno

Open the document

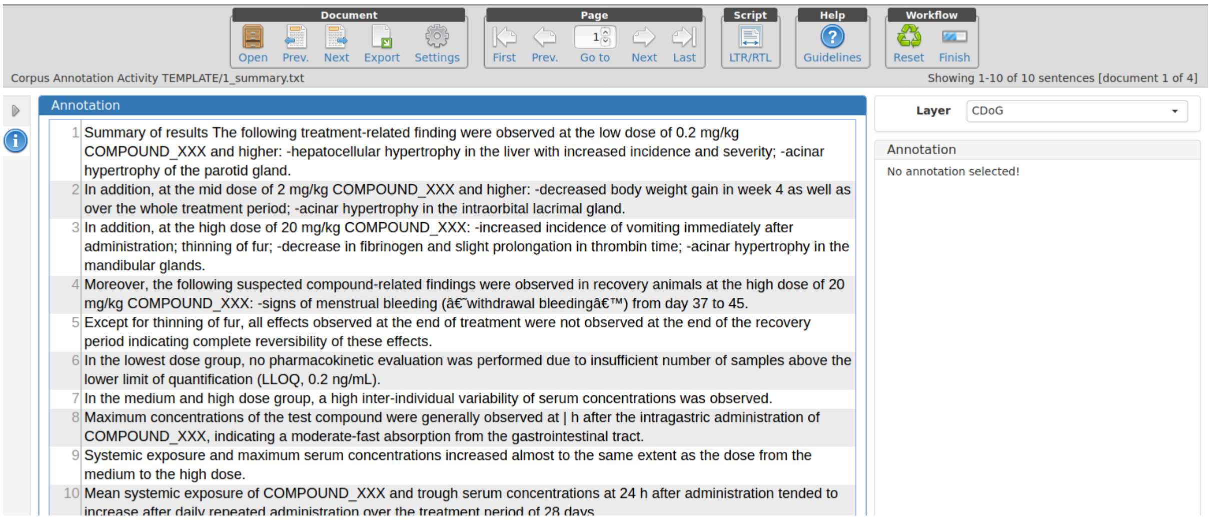

Figure 6. Document content overview.

The first sentence is relevant for our analysis; it contains two FINDINGS expressions separated by a coordinator: “hepatocellular hypertrophy in the liver with increased incidence and severity; -acinar hypertrophy of the parotid gland”. Following the annotation process, we annotate the FINDING expression. In the WebAnno tool, the FINDING and CDoG “Layers” can be appreciated on the right of the screen. To assign a FINDING to a text portion, we select the text and then assign the corresponding FINDING Layer. Figure 7 shows the FINDING expression in the sentence.

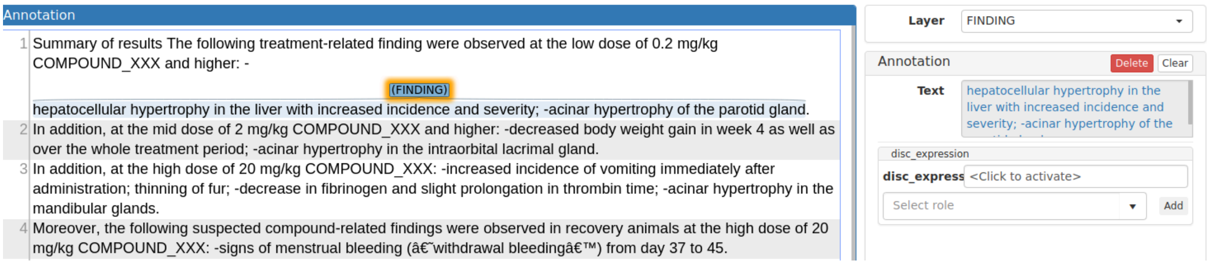

Figure 7. FINDING of the first sentence.

The next step is to identify the Compound Dose or Group (CDoG). In this case we can observe that in the first sentence a discontinued CDoG expression is present, “low dose of 0.2 mg/kg” as the first expression and “and higher” as the second expression.

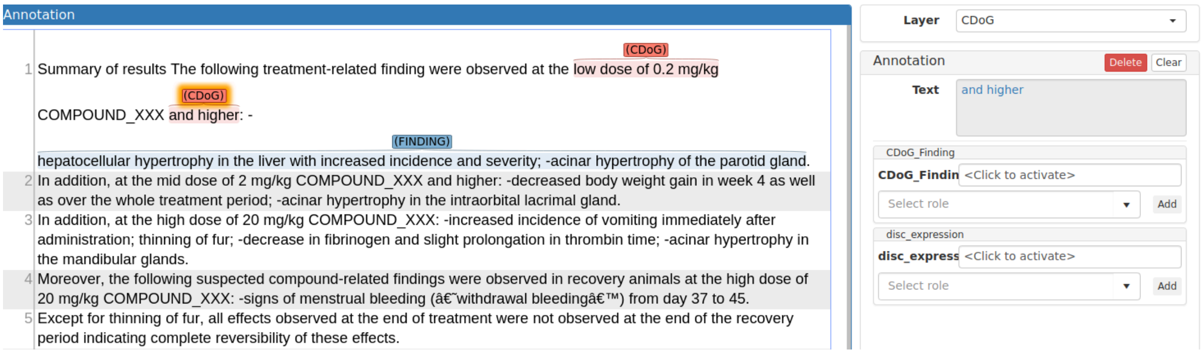

Figure 8. CDoG annotation.

The next step is to create the discontinued CDoG expression relation.

To generate a relation we need to select the first CDoG expression, then we can observe on the right the presence of a **relation** “disc_expression” with “<click to activate>”, on Figure 9, this field can be seen. Once we click to activate it will turn orange.

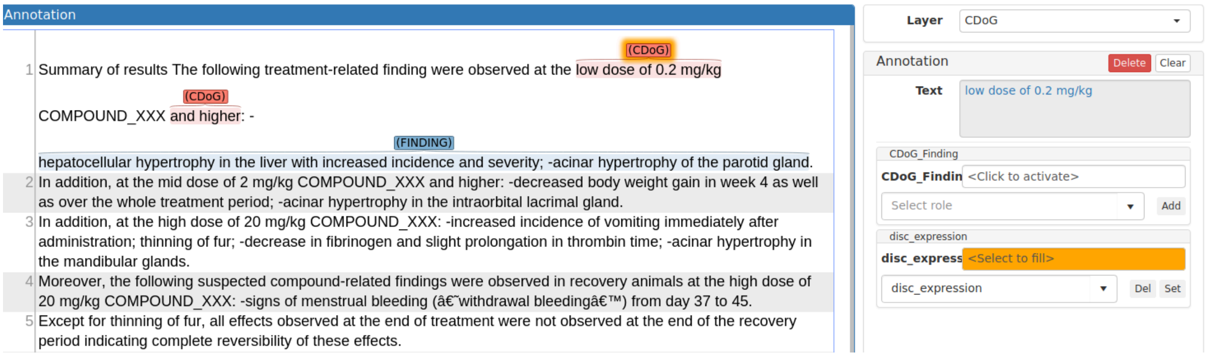

Figure 9. Creation of the discontinued CDoG relation between “low dose of 0.2 mg/kg” and “and higher”.

Now we have to select (double click) the second CDoG expression:“higher” to complete the relation; the link between the two expression will appear as shown in Figure 10.

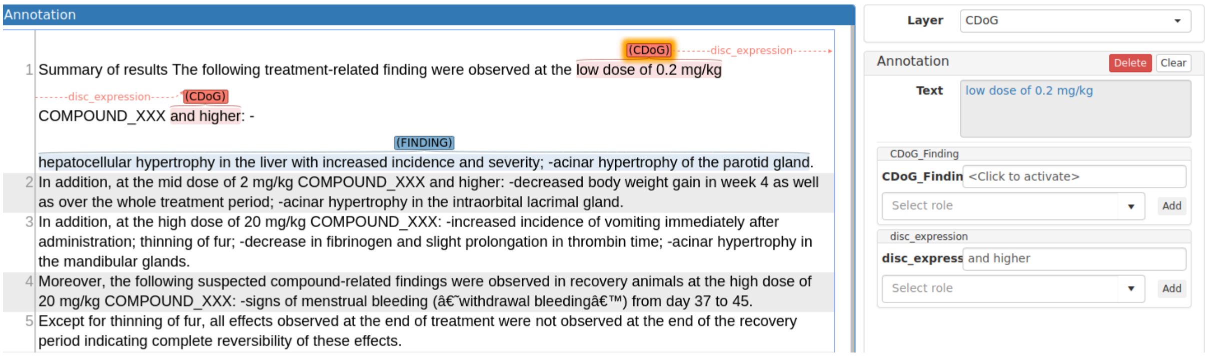

Figure 10. Overview of the discontinued CDoG relation between “low dose of 0.2 mg/kg” and “and higher”.

We proceed generating the relation between the CDoG and the FINDING, named “CDoG_Finding”. Remember that in this case the FINDING has to be linked with only one of the CDoG discontinued expressions; in this case we select the first discontinued CDoG “low dose of 0.2 mg/kg” and create the CDoG_Finding relation with the FINDING.

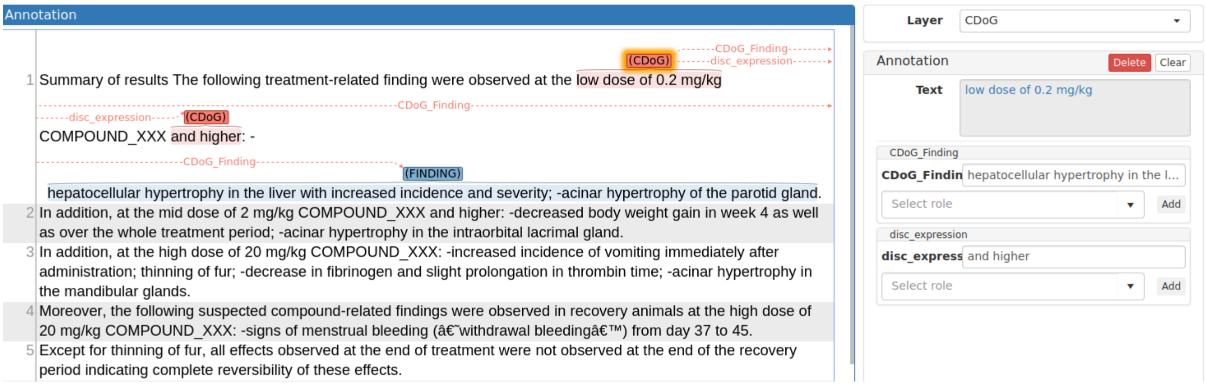

Figure 11. Overview of the relations between the DCoG and the FINDING.

Continue with the second sentence applying the same process. In this case we annotated first the CDoG expression that is also discontinued.

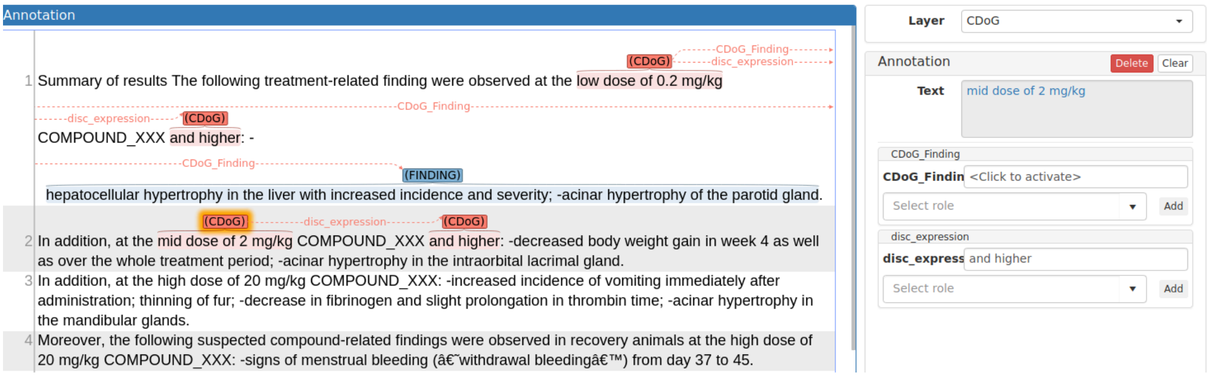

Figure 13.Annotation overview of the second sentence.

Two FINDING expressions are present in the second sentence: “decrease in body weight gain” and “acinar hypertrophy in the intraorbital lacrimal gland”. We annotated these two expressions as follows ...

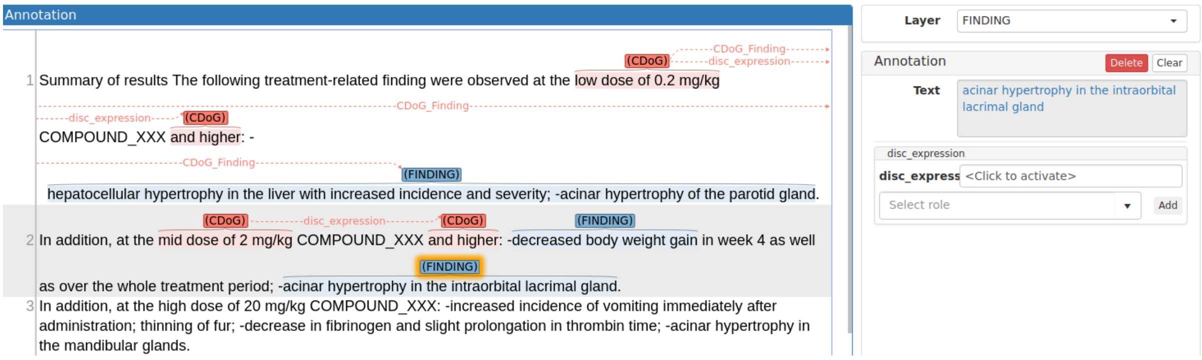

To end-up with the second sentence we generate the CDoG_Finding relations. Note that in this case there are two FINDING expressions that have to be related to the CDoG.

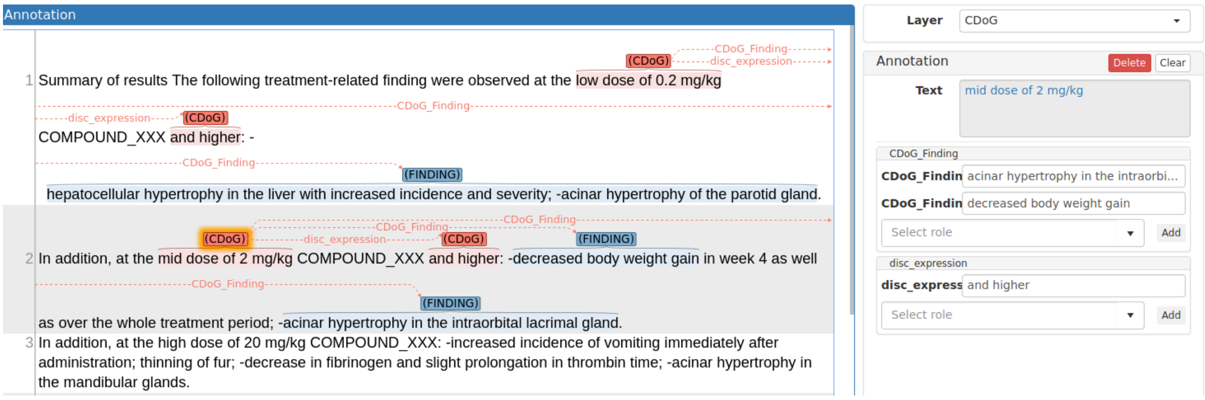

We repeat the annotation process for all the findings detected in the document.

Once the annotation is complete you have to finish the annotation process using the “Finish” button on the menu.

Important:

- Remember to check how many sentences are in the document, do not forget to annotate sentences of other pages.
- Do not forget to press “Finish” once you have completed the annotation process for a document.

Remember that the additional materials contains videos tutorial of the annotation process using WebAnno:
https://etransafe.bsc.es/nextcloud/index.php/s/jW2qdfnSPjNAXXy

5. FREQUENTLY ASKED QUESTIONS

5.1. 1st follow up meeting

When do we have to press the "Finish" button ? When we are done annotating a document or when we want to save our "session" ?

Webanno automatically saves our work every time we do anything; if we annotate/label a portion of text as a FINDING for example, it would automatically be saved.
There is no button to save your progress, this is done automatically.
We only have to click on "Finish" if we are done annotating that document.
You have to be sure because once you press "Finish" you will not be able to annotate the document again.
You need to click Finish for every single document you annotate, this is the way to indicate that you are done with that document.
If you incidentally Finish a document, please contact us and we will send it to the previous state.

Do we only annotate the CDoG and FINDING with relationships ? For some sentences mentioning objective or design, they may have dose info or the phenotypes used for monitoring. Do we need to annotate that info ?

You have to annotate all the CDoG and FINDING no matter if they are in a relation or not. If they are alone you have to annotate also.

For example, an important example:
"Light yellow discoloration of the serum and xiphoid cartilage was detected in one rat." Has to be labeled as a FINDING expression. Even if there is no CDoG to which associate these findings.

Secondary objective, doses Named Entity Recognition:
We extend this rule also to the doses that are alone, in order to be consistent with the rules. The doses annotation will serve to train a doses Named Entity Recognition model.
If you already finished some documents and did not include doses alone, leave it that way. And remember this rule for the following documents.
This is a secondary objective of the activity, realice that the primary is the

detection of FINDING and the DCoG associated (if there is any).

When annotating Finding, do we need to include negation. For example, test articles do not affect or cause specific findings.

No, you only have to annotate "positive" findings.
These should not be annotated:
"Test articles do not affect or cause specific findings."
"No body weight changes were detected".
"No clinical findings were detected after ...".

Why and when do we annotate “incidental” findings or “findings with no toxicological/biological” relevance ?

All the "positive" findings has to be included, even the incidental ones that are not treatment-related:

"Regarding clinical chemistry parameters, the increased GLDH observed only in one 30 mg/kg female was considered incidental."

We have to detect all the findings. Incidental or not incidental, if the finding is incidental. In this particular case you only need to label "increased GLDH observed" as FINDING.

Why do we have to detect all the findings ?, because this information will be included in the SR-domain.

This is different from the "no finding" examples.

Some sections are pure PK report, i.e. no tox findings but only plasma concentrations and AUC values. Is this still in scope of the exercise ?

The difference between a PK study and a Tox study is that PK study just delivers data (plasma concentrations, AUC,...) without the need of any assessment and the values are always treatment-related and never incidental. The tox study requires the respective assessment of the study director. Therefore it is out of scope for detecting "treatment-related".

In the design of the Study Report Domain, the PP domain (Pharmacokinetic Parameters) is included. But for me, I'm not a toxicologist, it is very difficult to

understand this type of domain and when there is a finding that implies Pharmacokinetic Parameters. So personally, for me it is unclear if this kind of information is a finding or not; I suppose that is not a finding.

"In males, AUC were 677 ± 182 h.ng/ml , 2191 ± 672 h.ng/ml, 12532 ± 8255 h.ng/ml for 5, 15 and 70 mg/kg/day respectively.
In females, AUC varied from 814 ± 315 h.ng/ml at the dose 5 mg/kg/day to 1754 ± 204 h.ng/ml at the dose 15 mg/kg/day and to 14010 ± 10843 h.ng/ml at the dose 70 mg/kg/day."

Out of scope.

Shall we annotate findings in the recovery group ?

Yes, all the "positive" findings have to be included.

As with treatment-related or no-treatment-related (incidental), there is a specific field that will be populated automatically in the "Study Phase", that could be DOSING, PRE-MATING, RECOVERY, GESTATION & POSTNATAL.

This part of assigning the study phase of the finding is done in a later step by a text-mining tool, you do not have to include any reference to that in the annotation process.

Do we repeat annotations for findings which are summarized in the final sentences, but have previously already been annotated ?

Yes. You have to annotate all the findings even if they are repeated in the text.

In this part of the activity, we do not have to take into account the repetition factor, that will be addressed in a future component that will detect this issue.

But at this moment, in the annotation of the corpus we have to include all the findings.

How to connect expressions which start with "decrease" and then stretch over various findings and doses?

The way to annotate the 26 sentences is okay. The disconnected FINDINGs with "decrease" are correct. But we found some issues regarding the dose

information. The “doses of” needs to be included, see next slide ….

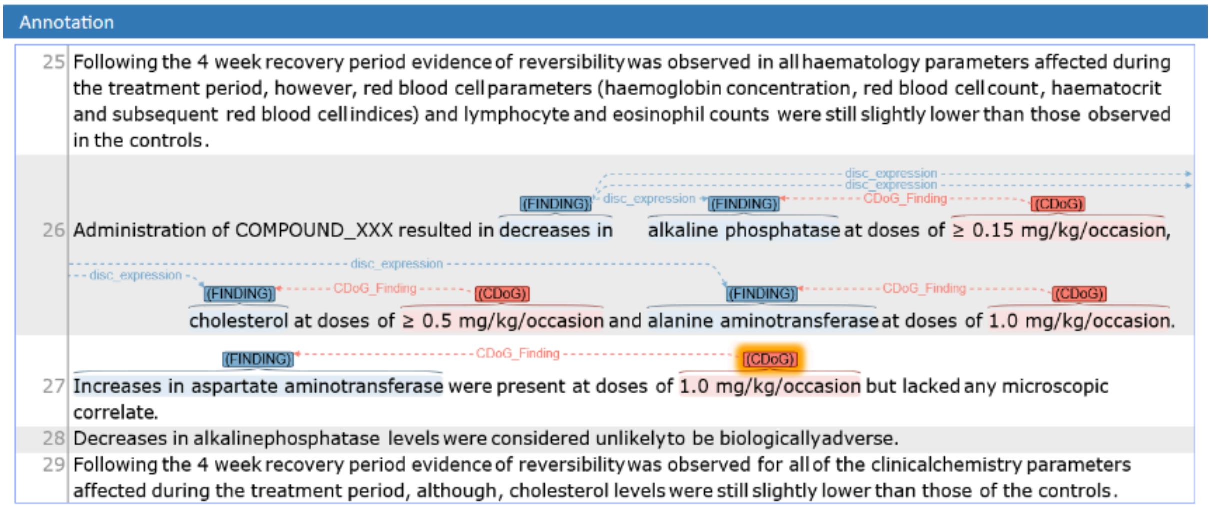

For example, including the “doses fix” in sentence 26 and 27 ⟶ we have to include the expression “dose of”. Also we complete the example labeling the 25 and 29 sentences which contains FINDINGS in the recovery period (another question) . We label the 28 sentence that contain a FINDING that is not adverse, but still a FINDING because all the positive findings need to be labeled (another question).

<u>Right way to annotated this example</u>:

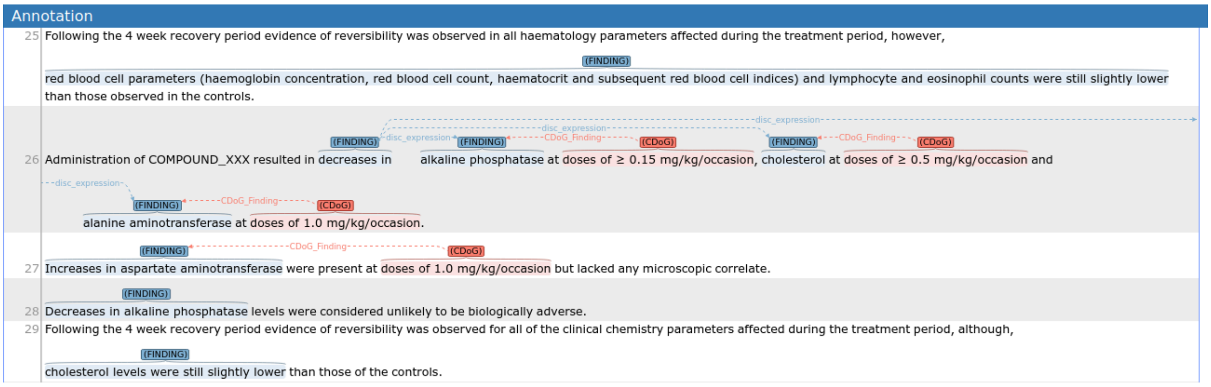

Added Doses rules:

> When the expression “… doses of >= 0.15 mg/kg day …” is present in the sentence not only the “>= 0.15 mg/kg day” needs to be labeled, it must include the expression“doses of >= 0.15 mg/kg day”.

Another example could be: "…. from the doses of 0.10 mg/kg to 0.50 mg/kg …". All this expression needs to be labeled as CDoG if it appears in a sentence.

Similar as above: "statistically significant reductions" but several parameters AND males and females at different doses (10 mg/kg vs. 75 mg/kg)?

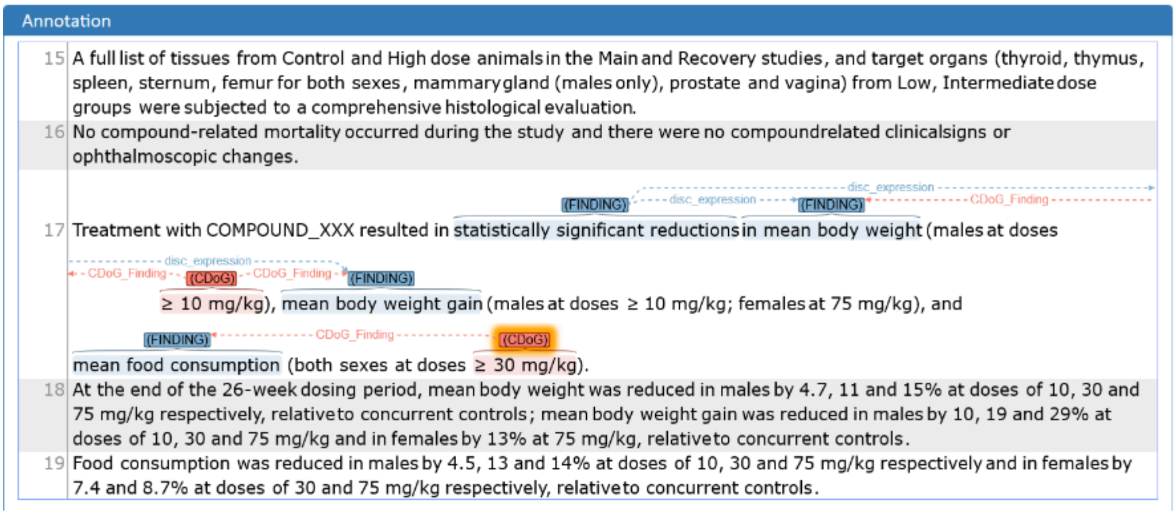

How we annotated different doses for different sex ?

We have to label as two separated CDoG and we do not have to include the sex, and relate the two CDoG with the FINDING. The sex for each doses will be automatically detected by later text-mining tools.

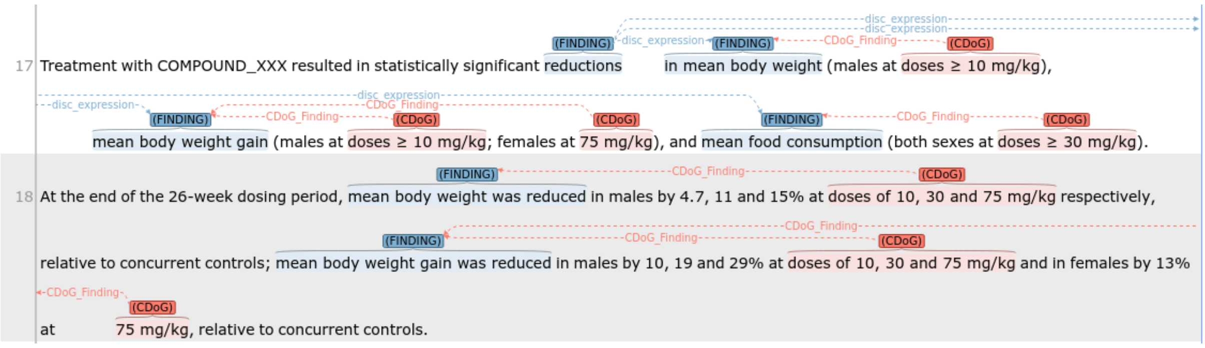

Also we remove statistically significant as part of the first finding expression, only reductions are included.

We have to include the numeric reduced or increase % when a finding is detected ?

No, we do not have to include this information in the labeling

"….. mean body weight gain was reduced in males by 10, 19 and 29% ….. "

Review sentence number 18 and 19 in the next slide to see the complete example of this finding.

All the annotations in this example:

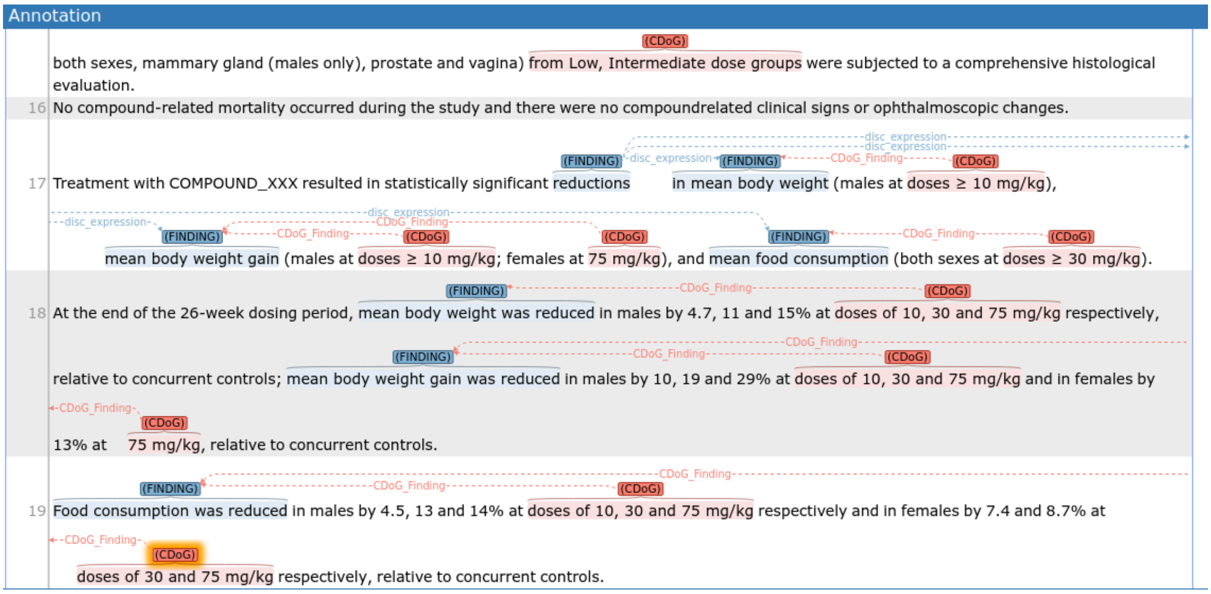
Annotation

both sexes, mammary gland (males only), prostate and vagina) from Low, Intermediate dose groups were subjected to a comprehensive histological evaluation.

16 No compound-related mortality occurred during the study and there were no compoundrelated clinical signs or ophthalmoscopic changes.

17 Treatment with COMPOUND_XXX resulted in statistically significant reductions in mean body weight (males at doses ≥ 10 mg/kg), mean body weight gain (males at doses ≥ 10 mg/kg; females at 75 mg/kg), and mean food consumption (both sexes at doses ≥ 30 mg/kg).

18 At the end of the 26-week dosing period, mean body weight was reduced in males by 4.7, 11 and 15% at doses of 10, 30 and 75 mg/kg respectively, relative to concurrent controls; mean body weight gain was reduced in males by 10, 19 and 29% at doses of 10, 30 and 75 mg/kg and in females by 13% at 75 mg/kg, relative to concurrent controls.

19 Food consumption was reduced in males by 4.5, 13 and 14% at doses of 10, 30 and 75 mg/kg respectively and in females by 7.4 and 8.7% at doses of 30 and 75 mg/kg respectively, relative to concurrent controls.

How should I define as CDoG if the Finding is found in all animals?

"Slight to moderate anemia with decreased reticulocyte counts and a moderate decreases in white blood cell count, mainly due to decreased neutrophils was seen in all animals from day 7."

In these cases, when a finding is detected with no particular doses or group definition you should not annotate a CDoG.

The same scenario if you have the following --- > change "all animals" for "one rat":

"Slight to moderate anemia with decreased reticulocyte counts and a

moderate decreases in white blood cell count, mainly due to decreased neutrophils was seen in one rat from day 7."

You do not have to annotate "one rat" as CDoG.

In this case, are "animals dosed daily" and "animals dosed twice a week" defined as CDoG?

"For animals dosed daily, dermal irritation was evident and included very slight to well-defined erythema in both males and females. Dermal irritation consisting of very slight erythema only was noted in animals dosed twice a week."

This case is nice and more complex, and I think that you are correct. It has to be annotated in that way. Because normally the qualification of the group or dose is "high dose group", "mid dose group" , "group 3" and so on, but in these cases the qualification of the group is the frequency: "animals dose daily" and "animals dosed twice a week".

El feedback para una posible versión 2.2 debe ser a partir de la 3er reunion.
