# Supplementary material for: PretoxTM: a text mining system for extracting treatment-related findings from preclinical toxicology reports
Source: J Cheminform. 2025 Feb 3;17:15. doi: 10.1186/s13321-024-00925-x (PMC11792311; doi:10.1186/s13321-024-00925-x)
Supplement: Supplementary file 2 — Additional file 2. [file 13321_2024_925_MOESM2_ESM.pdf]

Supplementary Materials of the PretoxTM Corpus

Javier Corvi

Abstract

The purpose of this document is to provide supplementary material for the PretoxTM Corpus. It is intended to complement and expand upon the procedures and methodologies presented in the development of the corpus. This document should be read in conjunction with the primary research, as it offers additional insights, detailed explanations, and supporting data that contribute to a deeper understanding of the results and conclusions outlined in the PretoxTM research. It includes data summaries, methodological clarifications, and other relevant information that enhances the overall context of the research.

1 Data gathering, organization and storage

Toxicological studies were provided by pharmaceutical companies affiliated with the European Federation of Pharmaceutical Industries and Associations (EFPIA) as part of the eTRANSAFE project, an Innovative Medicines Initiative (IMI) partnership. A total of 185 reports were collected during the data-gathering process. Of the 185 toxicological reports, text from the Summary, Conclusion, and Discussion sections was extracted using a semi-automated approach, resulting in 146 text sections containing a total of 2,933 sentences. These sections were selected because they contain expressions related to treatment-associated findings.

To centralize report collection and prevent security breaches, a secure eTRANSAFE Nextcloud instance was set up at the Barcelona Supercomputing Center (BSC) to store and safeguard the data.

Ensuring the security of the donated data was a mandatory requirement. The environment hosting the Nextcloud instance and the WebAnno annotation tool (Section 3.1) was designed to meet these security standards. A dedicated Virtual Machine (VM) instance was set up with two volumes: the root volume, containing the operating system (Ubuntu), and the data volume, where the data was stored. The data volume was encrypted using standard Linux mechanisms (LVM + dm-crypt), the same approach used by EGA-CRG [1] to protect sensitive human data. Daily backups were performed to ensure data integrity and accessibility, with the backup files also encrypted using the OpenSSL library.

The donated reports comply with GDPR (General Data Protection Regulation) anonymization and pseudonymization standards [2], as no personal information is present in the reports. Before including the text excerpts in the corpus, a final review was conducted to ensure the absence of any confidential information.

2 Annotation guideline development

An essential aspect of the annotation process is the definition of the annotation schema and the development of guidelines to follow throughout the task. The annotation guidelines were compiled into a document that includes a detailed description of the annotation schema, a tutorial on how to use the annotation environment, and a set of rules and tips to address the most common issues and questions encountered by annotators.

An annotation workshop was held on October 15, 2019, where the first version of the annotation schema was presented to toxicology experts and tested by annotating a subset of the corpus. The initial schema covered the entities listed in Table 1, as well as additional entities such as effect level, date of finding, study domain, and statistical significance of the observed effect. Relationships between the main entities (study test and finding) and the additional entities were also annotated.

Feedback from the experts during the annotation workshop revealed that the annotation schema did not meet expectations. The schema was too detailed, making it challenging to mark the numerous entities in the text and establish all the relationships between them. The process was slow and cumbersome, and the results were of limited value due to low consensus among the experts on the annotations.

A second version of the annotation schema, described in this article, was developed to facilitate the annotation task and simplify the process without compromising the expert knowledge necessary for identifying relevant findings in preclinical studies. This version focused on marking expressions rather than entities and defined two categories for annotation: ADVERSE_OBSERVATION and CDoG (Compose Dose or Group) expressions, along with their relationships. The remainder of this section analyzes the annotated expressions and relationships, as well as provides basic rules and examples to illustrate the annotation schema.

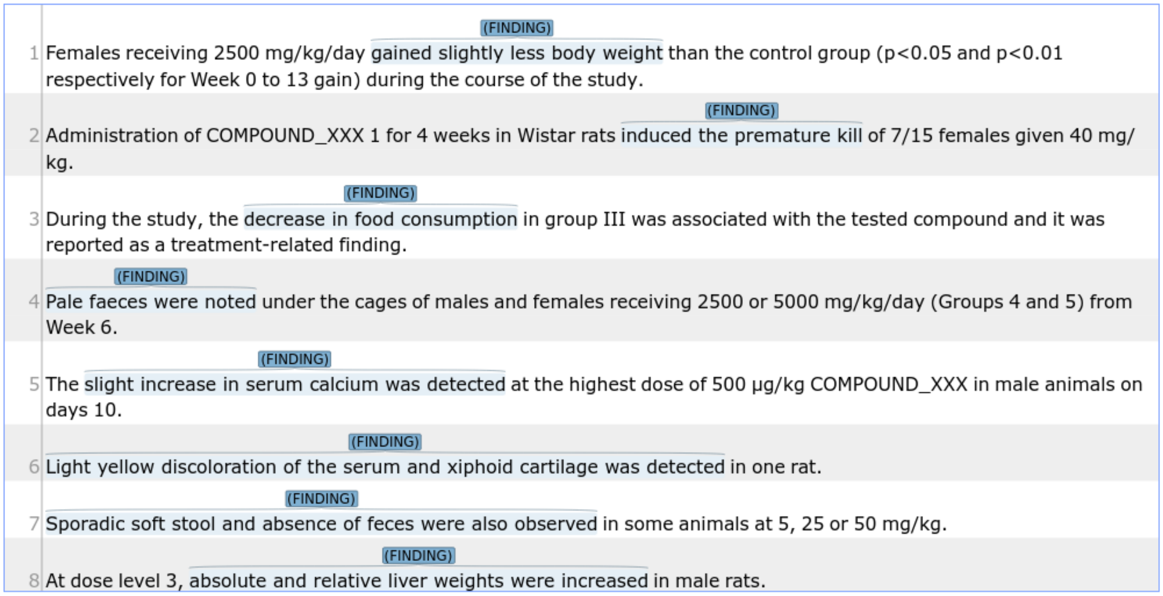

Figure 1: Examples of ADVERSE_OBSERVATION expressions. In the figure, ADVERSE_ OBSERVATION appears as FINDING, the old nomenclature used in the annotation schema.

2.1 ADVERSE_OBSERVATION

The ADVERSE_OBSERVATION category represents a complex linguistic expression that captures the manifestation of an adverse effect of the treatment. It encompasses a broad range of entities described in Table 1, including findings, study tests, manifestations, and specimens. Examples of ADVERSE_OBSERVATION expressions include: “gained slightly less body weight”, “pale feces were noted”, “slight increase in serum calcium” and “minimal to slight mineralization of the kidneys”, among others.

Important Note: The term ”ADVERSE_OBSERVATION” expression has replaced the old term ”FINDING” expression. However, to maintain consistency throughout the document, the old term ”FINDING” expression is used in the Figures.

In Figure 1, the ADVERSE_OBSERVATION annotations in sentences 1, 2, and 3 can be observed in the context of a toxicological study report. ADVERSE_OBSERVATION expressions should also include the verb indicating the action of observation; in sentences 4 and 5, for example, the findings include “were noted” and “was detected” as part of the annotated expressions. To account for the linguistic complexity of expressing findings, coordination of findings was permitted to be marked as a single concept. That is, an expression with several contiguous coordinated findings can be annotated as a single ADVERSE_OBSERVATION entity, provided they are all related to the same treatment. Examples are shown in sentences 6 and 7, where the coordinator “and” is included as part of the annotated expression.

2.2 CDoG (Compose Dose or Group)

In toxicology reports, treatments are typically expressed as the dose or group associated with the findings. For this reason, we refer to treatments as CDoG (Compose Dose or Group). In Figure 2, sentences 1, 2, and 3, examples of CDoG are presented, where treatments appear as dose or group identifications. During the description of preclinical findings, the dose may be implicitly referenced by citing a dosing level (sentence 8). In such cases, the dosing level expression “dose level 3” must be annotated as CDoG. An expression containing different coordinated treatments can be annotated as a single CDoG entity, provided they are all related to the same ADVERSE_OBSERVATION. Examples of this type of CDoG can be seen in sentences 4 and 7. Furthermore, a treatment can be defined as a mixed conjunction of these options, as demonstrated in sentences 4 and 5.

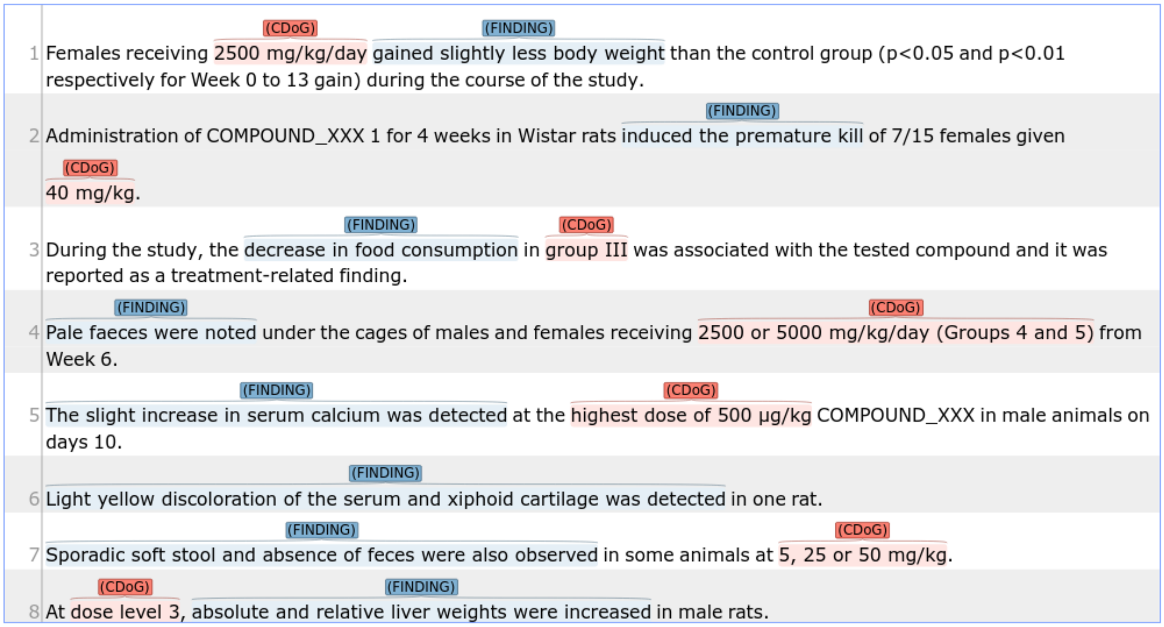

Figure 2: Examples of FINDING and CDoG (Compose Dose or Group) expressions. In the figure, ADVERSE_OBSERVATION appears as FINDING, the old nomenclature used in the annotation schema.

3 Relations

Relations are used to describe the types of associations between entities. In a treatment-related finding annotation schema, the most evident relationship connects a treatment with a finding: CDoG → ADVERSE_OBSERVATION (CDoG_adverse_observation). The CDoG_adverse_observation relation links a CDoG to an ADVERSE_OBSERVATION, indicating that a ADVERSE_OBSERVATION has been observed for a particular CDoG. Figure 3, sentences 1 to 8, presents various examples of this relation.
ADVERSE_OBSERVATION and CDoG are complex linguistic expressions that often encompass non-contiguous words and phrases; that is, there may be another expression with a different meaning interspersed within the ADVERSE_OBSERVATION or CDoG. To address this limitation, the disc_expression relation was created, allowing for the connection of interrupted expressions of the same entity. In Figure 4, sentences 1, 2, and 3 illustrate these types of relations for ADVERSE_OBSERVATION and CDoG.
In sentence 1, two ADVERSE_OBSERVATION expressions are linked, where the first part describes the specimen and the second part describes the adverse effects. Sentence 2 provides an example of a CDoG discontinuous expression and its relation to an ADVERSE_OBSERVATION expression. Sentence 3 shows examples of interrupted expressions; the ADVERSE_OBSERVATION expression "reductions in" is associated with three non-contiguous expressions: "mean body weight," "mean body weight gain," and "mean food consumption," resulting in a total of three ADVERSE_OBSERVATION.
This example, along with the sentences in Figure 5, highlights the linguistic complexity involved in describing findings in preclinical toxicological studies.
The annotation guideline are also available as a separate document in the supplementary materials.

3.1 Annotation tool

To facilitate the annotators' tasks, a tool was needed that would allow the creation of annotations from any device running a web browser. Furthermore, the annotations needed to be stored on a central server for processing. Based on the required functionalities and a prior review of manual annotation tools [3], WebAnno [4] was selected as the annotation software. In that review, WebAnno received the highest global score (0.81) according to a benchmark that considered 26 criteria. An instance of WebAnno was made available on the secure eTRANSAFE server (described in Section 1) for use during the annotation activity and the harmonization process.

Table 1: The PretoxTM entity model. Named entities related to treatment-related findings.

Entity	Description	Examples
Study Test	Experimental method used to assess/measure the effect of a compound in the animal model.	body weight, food consumption, reticulocytes, albumin, weight
Manifestation	Manifestation of Study Test.	increase, decrease, lower, changed, alteration
Finding	Adverse effect produced after the administration of the compound in the animal model.	vomitus, hypertrophy, pale, ataxia, atrophy
Specimen	Specimen in which the effect was observed.	faeces, kidney, liver, heart, salivary gland, thymus, urine
Dose	Dose in which the effect was observed.	20 mg/kg
Sex	Sex of the animals in which the effect was observed.	female, male, both sexes
Group	Group of animals in which the effect was observed.	group 1, group A

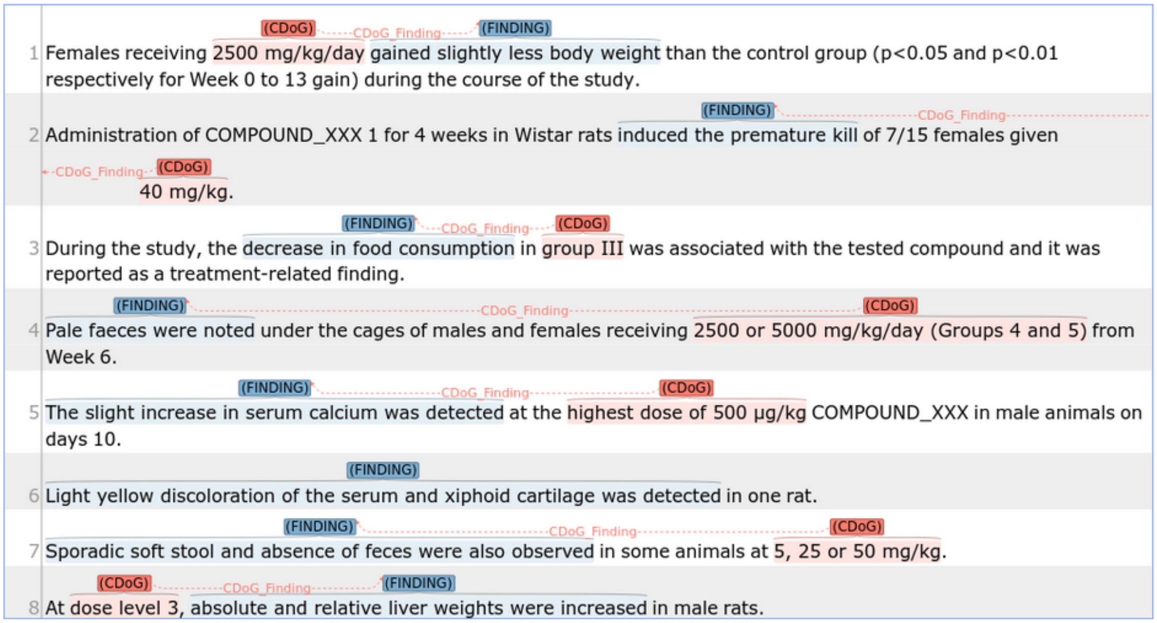

Figure 3: Examples of CDoG and ADVERSE_OBSERVATION relations. In the figure, ADVERSE_ OBSERVATION appears as FINDING, the old nomenclature used in the annotation schema.

4 Annotation activity

Once the relevant sections were extracted and the annotation guideline were consolidated, the process continue with the annotation activity event. For this activity, a team of 14 experts participating in the eTRANSAFE project were recruited.

The 2,933 sentences were evenly distributed across four sets, consisting of approximately 733 sentences per set. Each set was annotated by a different group, including two groups of three annotators and two groups of four annotators. This design aimed to ensure that each relevant section was annotated by at least three experts.

Throughout the annotation activity, several questions and uncertainties arose concerning the annotation task and the annotation schema. These issues were addressed during the weekly follow-up meetings. Furthermore, discussions among the experts regarding adverse effects, treatment options, and other toxicological aspects enriched these meetings. The resolutions of these concerns were compiled into the frequently asked questions (FAQs) section of the annotation guidelines, which contributed to the final version (v2.1) of the document.

The entire corpus was annotated over a period of eight weeks, with 21.8% of the sentences annotated by four experts, 61.9% by three experts, and 16.3% by two experts. As a result, 83.7% of the corpus was annotated by

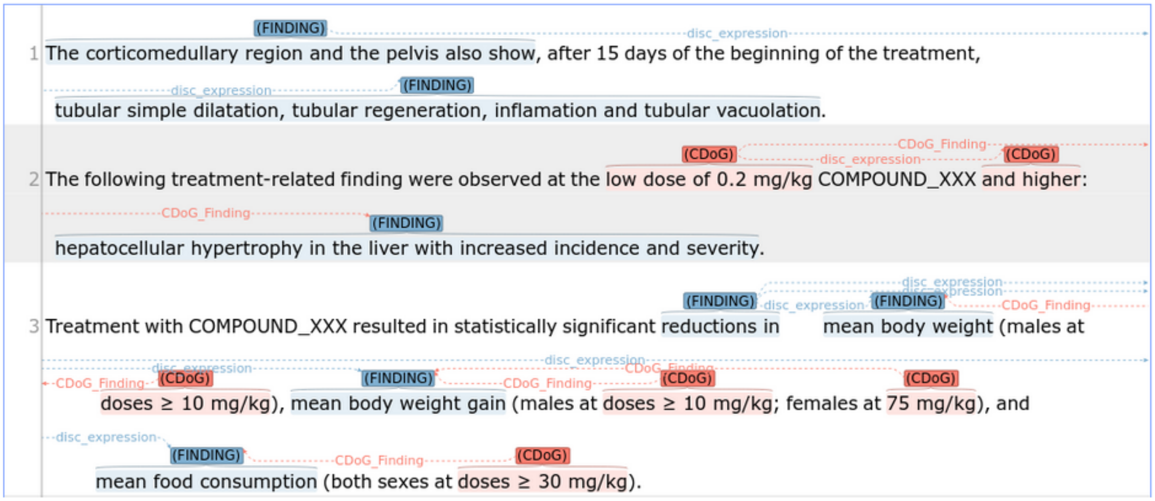

Figure 4: Examples of non-contiguous relations. In the figure, ADVERSE_ OBSERVATION appears as FINDING, the old nomenclature used in the annotation schema.

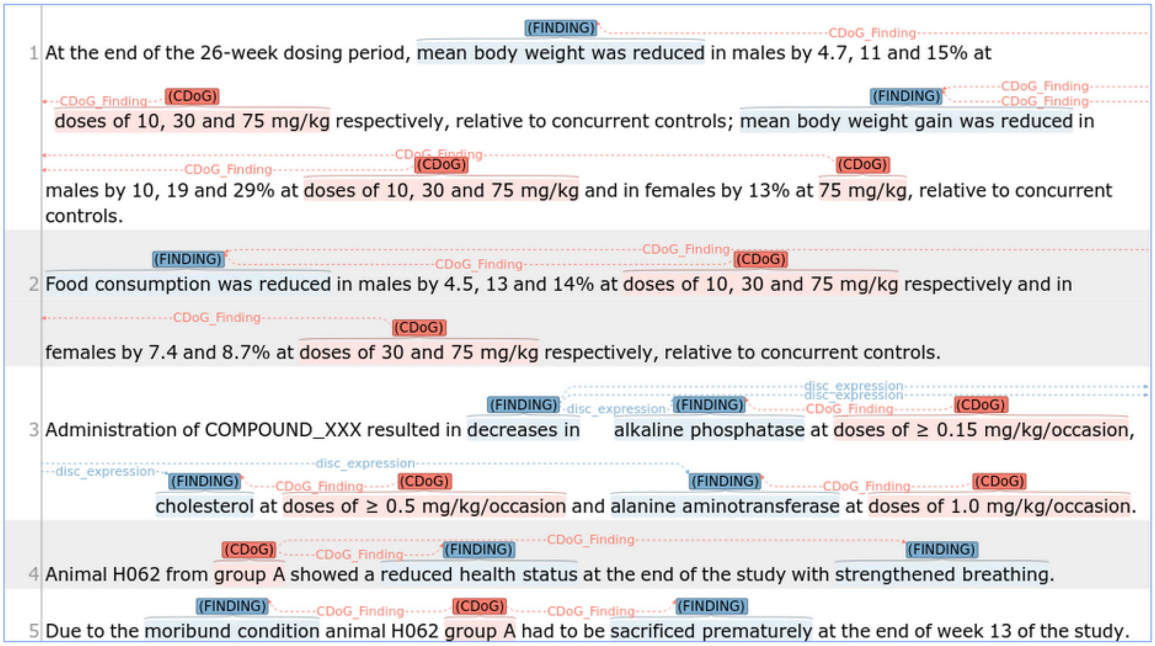

Figure 5: Different complex examples of FINDING and CDoG expressions. In the figure, ADVERSE_ OBSERVATION appears as FINDING, the old nomenclature used in the annotation schema.

at least three experts. In one of the groups that had four annotators, two did not complete the annotation tasks and were subsequently discarded.

5 Inter-annotator agreement

Once the corpus had been annotated by the experts, we calculated the Inter-annotator Agreement (IAA) as a metric of annotation quality. The IAA is a statistical measure that evaluates the degree of consensus among the annotators for a given annotation task.

Calculating and analysing the results of the inter-annotation agreement (IAA) was essential to identify the differences that emerged between the experts during the annotation activity. The IAA was calculated using Krippendorff's Alpha with the nominal/strict method [5, 6]. We first computed the IAA for each of the four groups and then averaged the results, obtaining 0.73 for ADVERSE_OBSERVATION expressions, 0.74 for CDoG expressions and 0.76 in general. Table 2 shows the complete results.
The calculation of IAA was useful for detecting outliers, particularly from one annotator who significantly lowered the average annotation score; this was detected in Group 1. This information was taken into account during the harmonization process and conflict resolution meetings.
To review the IAA in more detail and to reproduce its results, there is a Jupyter notebook available at: https://gitlab.com/pretoxtm/pretoxtm-corpus/-/blob/main/annotated-corpus/development/IAA_krippendorffs_alpha.ipynb

Table 2: Inter Annotator Agreement using Krippendorff's Alpha

	ADVERSE_OBSERVATION	CDoG	General
Group 1	0.67	0.70	0.70
Group 2	0.76	0.75	0.78
Group 3	0.80	0.76	0.80
Group 4	0.70	0.75	0.74
AVG	0.73	0.74	0.76

6 Harmonization and conflict resolution

Following the annotation activity, a harmonization process was conducted to generate a consensus corpus from the various annotations made by the experts. This harmonization process involved reviewing the annotations made by the experts in each section and selecting the final annotation, with priority given to the criteria described in the annotation guidelines. To achieve this, it was also important to analyze the IAA results to identify the differences that emerged between the experts during the annotation activity.
The first step of the process involved developing harmonization guidelines. To achieve this, a random set of annotations was reviewed to extract the most common conflicts and create corresponding resolution rules. This process was iterative; in each iteration, new rules were added and reviewed as a complete set to ensure consistency.
The next step in the harmonization process was the curation of the experts' annotations by applying the rules outlined in the harmonization guidelines. Conflicts not addressed in the guidelines or those requiring decisions on toxicological aspects were marked as 'PENDING' to be resolved later during the conflict resolution meetings. The harmonization guideline are also available as a separate document in the supplementary materials.
At the end of the harmonization process, there were a total of 190 out of 200 conflicts in a PENDING state. To address these conflicts, we convened two toxicologists who had participated in the annotation activity. The conflict resolution approach involved creating seven Typeform (https://www.typeform.com), equally divided, in the form of surveys, where we compiled all relevant information about the conflicts. Each toxicologist was assigned conflicts concerning findings in which they had not previously been involved in the annotations. To provide additional support, we held two final meetings to clarify any remaining questions regarding the resolution of the conflicts. This task was completed without any significant issues.

7 Post-processing step and final validation

After resolving all conflicts, a post-processing step was applied to automatically standardise the ADVERSE_OBSERVATION and CDoG expressions by mapping entities defined in the PretoxTM Data Model (Table 1). The mapping was performed using CDISC SEND, eTOX, UMLS, and finally the DNorm tool [7]. It is important to empathize that only the mapped entities are generated within the ADVERSE_OBSERVATION and CDoG expressions. Lastly, a final review by an expert was conducted to ensure that entities were accurately mapped to the expressions.
Out of 1,800 ADVERSE_OBSERVATION expressions we recorded a list of 508 that did not contain any mapped finding or study test entity. In these cases, the expert analysed and decided on the appropriate setting for these expressions. In such cases, two situations could occur:
1. The expert decides to annotate individual entities and normalize the expression. For example:

- bone stiffness →the expert indicates that ”stiffness” is the *finding*, while ”bone” had already been mapped as *specimen* by the automatic process.
- enlarged livers →the expert notes that ”enlarged” is the *finding*, while ”livers” had already been mapped as *specimen* by automatic process.
- lesions in the stomach →”lesions” is annotated as *finding* and ”stomach” as *specimen*.

2. The expert decides that is not possible to identify individual entities and normalize the expression, so the entire expression is annotated as a finding entity. Some examples are:

- impaired general condition
- ultrastructural findings
- severe clinical signs
- hepatocellular changes
- pronounced alterations

In most of these cases, the *findings* were general and not very descriptive, but they implied an adverse effect. Appendix A show an example list of 50 of these ADVERSE_OBSERVATION expressions.

8 Final Corpus

Finally, a corpus of sentences was generated based on the premise that any sentence containing an abnormal observation is considered relevant, while those without are considered non-relevant. Table 3 provides a detailed overview of the PretoxTM corpus, along with additional relevant information.

Table 3: PretoxTM Gold Standard Corpus statistics.

**Category**	**Count**
Relevant sentences	1,264
Non-relevant sentences	1,669
ADVERSE_OBSERVATIONs	1,800
CDoGs	1,674
Study Test	1,150
Manifestation	1,044
Finding	1,880
Specimen	1,395
Dose	1,322
Sex	748
Group	341
Tokens	86,421
Sentences	2,933

Details about the development of the PretoxTM Corpus are available in the GitLab repository at https://gitlab.com/pretoxtm/pretoxtm-corpus. The PretoxTM Corpus is publicly available and free to access on Zenodo at https://zenodo.org/record/7858116; and on HuggingFace at https://huggingface.co/datasets/javicorvi/pretoxtm-dataset. The PretoxTM Corpus is licensed under a CC BY-SA 4.0 licence.

Appendix A: Examples of ADVERSE_OBSERVATION expressions without entity mapping

- cardiotoxicity
- clear toxic effects
- clinical pathology findings
- clinical signs
- dermal lesions
- disordered bone
- electrolyte imbalances
- exaggerated pharmacological (hormonal) effects
- found dead
- general toxicity
- haematological effects
- hemogram results returned to baseline
- hepatocellular changes
- histopathological findings
- inflammatory changes
- intestines were empty
- intolerable side effects
- marked clinical signs
- marked lesions, both in-life and histopathologically
- microscopic alterations
- microscopic changes
- minimally prolonged activated partial thromboplastin times
- moderately increased M:E ratio
- moribundity
- moribundity occurred
- morphologic cardiac effects
- necrotic changes of mitochondria and cytoplasm
- nutritional state was slightly reduced
- ovulation was not proceeding normally
- pancreatic lesions
- panenteritis
- reduced health status
- rooting in the bedding material
- rosette formation and agglutination of erythrocytes
- several exaggerated pharmacological effects
- severe clinical signs
- showed a reduced health status
- signs of a reaction
- signs of general toxicity
- signs of impaired condition
- spontaneous alterations
- sporadic food left
- strengthened breathing
- subsequent deterioration of the health condition
- testicular changes
- testicular findings
- total WBC counts dropped
- ultrastructural findings
- very few clinical signs
- very sensitive to noise

The complete list of this non mapped adverse observations are available at
https://gitlab.com/pretoxtm/pretoxtm-corpus/-/blob/main/annotated-corpus/development/post-processing/final-visual-inspection/no_mapped_findings_expert_revision.txt

References

1. Ilkka Lappalainen, Jeff Almeida-King, Vasudev Kumanduri, Alexander Senf, John Dylan Spalding, Saif Ur-Rehman, Gary Saunders, Jag Kandasamy, Mario Caccamo, Rasko Leinonen, Brendan Vaughan, Thomas Laurent, Francis Rowland, Pablo Marin-Garcia, Jonathan Barker, Petteri Jokinen, Angel Carreño Torres, Jordi Rambla de Argila, Oscar Martinez Llobet, Ignacio Medina, Marc Sitges Puy, Mario Alberich, Sabela de la Torre, Arcadi Navarro, Justin Paschall, and Paul Flicek. The european genome-phenome archive of human data consented for biomedical research. *Nature genetics*, 47(7):692–695, Jul 2015. 26111507[pmid].
2. Antonia Vlahou, Dara Hallinan, Rolf Apweiler, Angel Argiles, Joachim Beige, Ariela Benigni, Rainer Bischoff, Peter C. Black, Franziska Boehm, Jocelyn Céraline, George P. Chrousos, Christian Delles, Pieter Evenepoel, Ivo Fridolin, Griet Glorieux, Alain J. van Gool, Isabel Heidegger, John P.A. Ioannidis, Joachim Jankowski, Vera Jankowski, Carmen Jeronimo, Ashish M. Kamat, Rosalinde Masereeuw, Gert Mayer, Harald Mischak, Alberto Ortiz, Giuseppe Remuzzi, Peter Rossing, Joost P. Schanstra, Bernd J. Schmitz-Dräger, Goce Spasovski, Jan A. Staessen, Dimitrios Stamatialis, Peter Stenvinkel, Christoph Wanner, Stephen B. Williams, Faiez Zannad, Carmine Zoccali, and Raymond Vanholder. Data sharing under the general data protection regulation. *Hypertension*, 77(4):1029–1035, April 2021.
3. M. Neves and J. Seva. An extensive review of tools for manual annotation of documents. *Briefings in Bioinformatics*, 22:146 – 163, 2021.
4. Seid Muhie Yimam, Iryna Gurevych, Richard Eckart de Castilho, and Chris Biemann. WebAnno: A flexible, web-based and visually supported system for distributed annotations. In *Proceedings of the 51st Annual Meeting of the Association for Computational Linguistics: System Demonstrations*, pages 1–6, Sofia, Bulgaria, August 2013. Association for Computational Linguistics.
5. Ron Artstein and Massimo Poesio. Survey Article: Inter-Coder Agreement for Computational Linguistics. *Computational Linguistics*, 34(4):555–596, 2008.
6. Santiago Castro. Fast Krippendorff: Fast computation of Krippendorff's alpha agreement measure. https://github.com/pln-fing-udelar/fast-krippendorff, 2017.
7. Robert Leaman, Rezarta Islamaj Doğan, and Zhiyong Lu. DNorm: disease name normalization with pairwise learning to rank. *Bioinformatics*, 29(22):2909–2917, 08 2013.
