# Supplementary material for: PretoxTM: a text mining system for extracting treatment-related findings from preclinical toxicology reports
Source: J Cheminform. 2025 Feb 3;17:15. doi: 10.1186/s13321-024-00925-x (PMC11792311; doi:10.1186/s13321-024-00925-x)
Supplement: Supplementary file 3 — Additional file 3. [file 13321_2024_925_MOESM3_ESM.pdf]

777365 – eTRANSAFE

Enhancing TRANslational SAFEty Assessment through Integrative Knowledge Management

Text-Mining Harmonization Guideline: Treatment-related findings

**Authors**	Javier Corvi (BSC), Emilio Centeno (IMIM)
**Contributors**	Laura Furlong (IMIM), Salvador Capella-Gutierrez (BSC), José María Fernández (BSC)

DOCUMENT HISTORY **2**
1. INTRODUCTION **3**
2. HARMONIZATION (CURATION) PROCESS **4**
3. HARMONIZATION RULES comentarios **4**

DOCUMENT HISTORY

Version	Date	Description
0	10/08/2020	Draft version of the harmonization guideline.
1	18/09/2020	First version of the harmonization guideline.

1. INTRODUCTION

The text-mining task requires a corpus conformed by documents with annotations of treatment-related findings (TRFs). A final step in the creation of the corpus consists in generating consensus among the different annotations made by the experts. We call this process "the harmonization process", also known as the “curation process”.

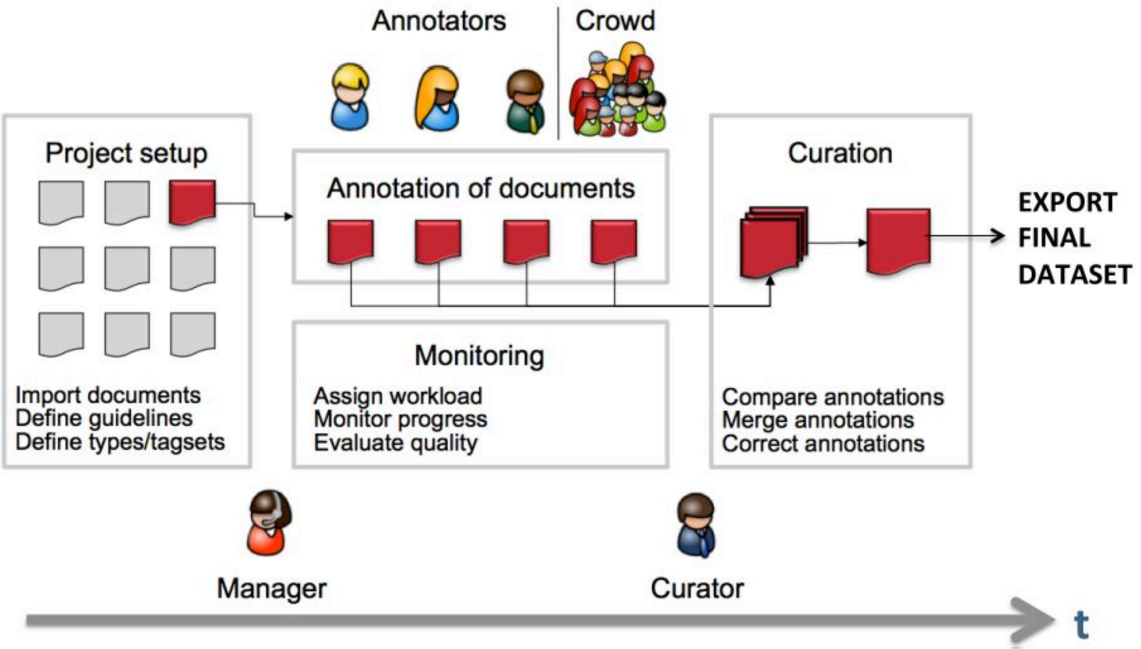

Figure 1 (from the WebAnno web page): A global workflow for the creation of the corpus

This document contains a comprehensive set of rules that resolve most of the discrepancies found between annotators. By applying a fixed set of rules we try to keep traceability in the

harmonization process. I.e: We can explain every final annotation as the result of applying a rule (or combination of rules) to the annotations made by the experts.

2. HARMONIZATION (CURATION) PROCESS

The harmonization workflow starts with a pre-annotated corpus where every document is annotated by several (from 3 to 4 in our case) experts. Since annotators may disagree in some annotations, we need to generate a consensus for each annotation.

In order to avoid subjectivity or arbitrariness in the conflict resolution, the first step in this harmonization process is the definition of a comprehensive set of rules to deterministically resolve any lack of agreement. This document is manually tailored by curating some sample documents and inspecting the resulting rules in an iterative process.
After applying the ruleset, there will be annotations marked as *PENDING* because they need a final decision that should be made by an expert. Once the *PENDING* annotations have been resolved, will have a consensus corpus. Figure 1 illustrates the workflow.

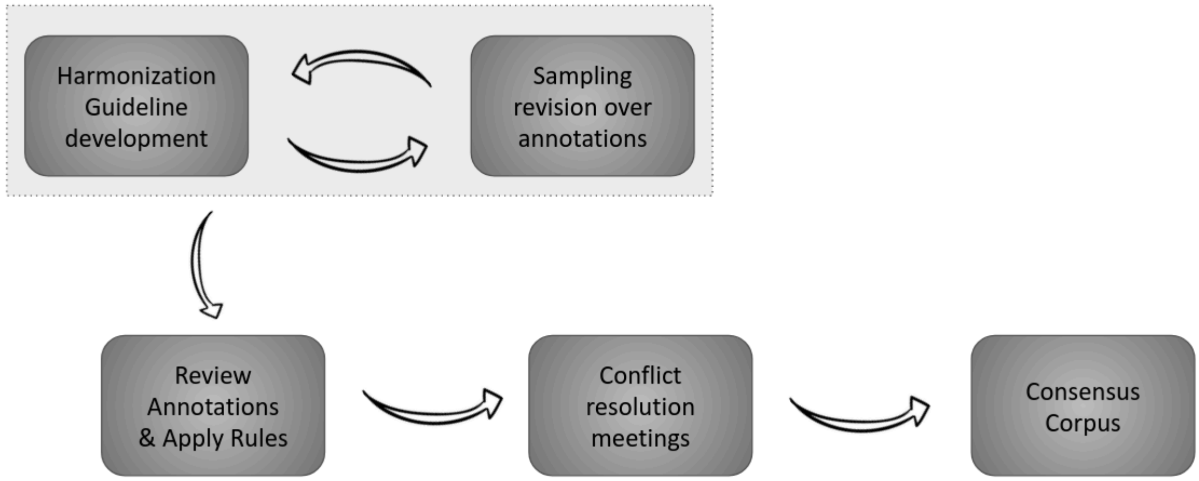

Figure 2. The different stages of the harmonization process.

3. HARMONIZATION RULES   comentarios

Important Note: The term "**ADVERSE_OBSERVATION**" expression has replaced the old term "FINDING" expression. However, to maintain consistency throughout the document, the old term "FINDING" expression is used.

The main objective of the harmonization rules is to deterministically resolve any lack of agreement. Cases not considered in this ruleset will be marked as *PENDING*, that will be

finally resolved by an expert. Under no circumstance will a toxicology disagreement be resolved without the participation of experts. The consensus corpus, will contain just annotations backed by experts

1. Annotations that do not follow the annotation guidelines will be ignored

2. Any valid (according to the annotation guidelines) annotation made by a majority of experts goes to the consensus corpus.

3. In case of tie, the preferred criteria in the annotation guidelines will prevail and will be marked as ACCEPTED. In case that the annotations guidelines can not resolve the conflict, then it will be marked as PENDING.

   E.g: the FINDING expressions; “during the treatment, body weight decrease” (annotator #1) and “body weight decrease” (annotator #2); following the annotation guideline the annotation of the annotator #2 is ACCEPTED and goes to the consensus corpus.

4. If there are different annotations trying to annotate different things (different criteria about a CDoG or a Finding or their relation), it will be marked as PENDING.

5. Regarding the lack of annotations in an entity. I.e: an entity was annotated by an expert/s annotator and other/s did not marked it as an entity

   5.1. In the case of a FINDING entity, the no-annotation is taken into account. In case of tie, the decision will be marked temporarily as PENDING for conflict resolution

   5.2. In the case of CDoG we only take into account the annotations made, but not the not-made. This exception has been added because there were some doubts regarding the annotation of CDoGs during the annotation process and we feel that some annotators may have skipped CDoGs.

6. In case that a relationship was annotated by an expert/s annotator and other/s did not marked it, we will take into account the annotations made, but not the not-made

7. Negated FINDINGS will be ignored (i.e: not annotated), as it was explained in the annotation guidelines. E.g.: “did not induce adverse organ alterations” or “clinically tolerated”.

8. The increase in exposition will not be considered a FINDING, as it was defined during one of the text-mining annotation meetings.

9. Contiguous region over discontinued expressions: If a contiguous region is well annotated (same type of entity with just coordinators), then the single contiguous annotation will be ACCEPTED in order to keep on with the annotation guidelines.

10. Rules about the spanning of an annotation in the text. Basically, in this rule we follow the annotation guidelines, to mention two of the most common spanning conflicts:

    10.1. Included words in the CDoG expressions:

    a) Take the expression with the word "dose" and expressions like "at dose of", etc as the preferred option, as was indicated in the annotation guideline.

    E.g:

- "dose of 60/64 mg/kg/day (base/salt)" → annotator #1
- "at dose of 60/64 mg/kg/day (base/salt)" → annotator #2 (preferred option)
- "60/64 mg/kg/day" → annotator #3

    b) For dose intervals, take the minimum relevant words to express the scope of the interval.

    - Example #1:
    - "at the mid dose and higher" → annotator #1 (preferred option)
    - "mid dose and higher" → annotator #2

    - Example #2:
    - "*doses ≥ 6 mg/kg/day*" → annotator #1 (preferred option)
    - "*6 mg/kg/day*" → annotator #2
    - "*≥ 6 mg/kg/day*" → annotator #3

    - Example #3:
    - "*from the low dose group onwards*" → annotator #1 (preferred option)
    - "*the low dose group onwards*" → annotator #2
    - "*low dose group*" → annotator #3

    c) The treatment (dosing) has priority over the group. Therefore, in case of describing a group by the dose and the sex, we just keep the dose.

    - Example:

- "*30 mg/kg males*" → annotator #1
- "*30 mg/kg*" → annotator #2 (preferred option)

d) The increment of the dosing over time will not be annotated.
   - Example:
   - "high dose following multiple doses" → annotator #1
   - "high dose" → annotator #2 (preferred option)

10.2. Inclusion of verbs in the FINDING expression:

a) Verbs will be included in the FINDING expression as it was indicated in the annotation guidelines.
   - Example #1:
   - "*pale faeces were noted*" → annotator #1 (preferred option)
   - "*pale faeces*" → annotator #2
   - Example #2:
   - "*water consumption*" → annotator #1
   - "*water consumption was decreased*" → annotator #2 (preferred option)

11. Any case not described neither by the harmonization rules or the annotation guidelines will be marked as PENDING.

12. The doubtful annotations when exist conflict among annotators; even if an annotation has been made by a majority of the annotators, will be marked as PENDING.
