# Supplementary material for: PretoxTM: a text mining system for extracting treatment-related findings from preclinical toxicology reports
Source: J Cheminform. 2025 Feb 3;17:15. doi: 10.1186/s13321-024-00925-x (PMC11792311; doi:10.1186/s13321-024-00925-x)
Supplement: Supplementary file 4 — Additional file 4. [file 13321_2024_925_MOESM4_ESM.pdf]

PretoxTM

Preclinical toxicology text mining

User Manual

Summary 3
Access to PretoxTM 3
Access to PretoxTM from the eTRANSAFE ToxHub 3
Home 4
Prompt Execution 5
Upload Reports 5
PretoxTM pipeline 6
- Run Workflow 6
- Report and Workflow status 7
- No sections detected 7
- Run without section extraction 8
- Workflow failed status 8

Open and validate report findings 9
- Findings table 9
- Textual evidence 9
- Validate the extracted findings 10
- Non-controlled terminology 11
- Edit findings 11
- Add new findings 12
- Submit information to the SR-Domain Web Editor 12
- Export findings 14
- Relevant Sentences 15
- Historical changes 15
- Plain Text 16

Remove Reports 16
Open PDF or Txt documents 16

Summary

PretoxTM (Preclinical Toxicology Text Mining) extracts treatment-related findings from toxicology reports using Natural Language Processing (NLP) techniques. The extracted findings are then presented in a well-defined user interface for validation by toxicology experts.

This document is intended for users of PretoxTM to explain the system's functionalities, using examples and images for each feature. PretoxTM was developed as part of the eTRANSAFE project, making the system accessible through the eTRANSAFE ToxHub environment. Later, efforts were made to allow PretoxTM to be installed and used independently of the eTRANSAFE project. The tool's installation is straightforward and can be completed by following the steps outlined at https://gitlab.com/pretoxtm/pretoxtm.

Access to PretoxTM

PretoxTM can be accessed directly after a standard installation using basic login credentials (Figure 1). This distinction is made because PretoxTM is also available within the eTRANSAFE project environment.

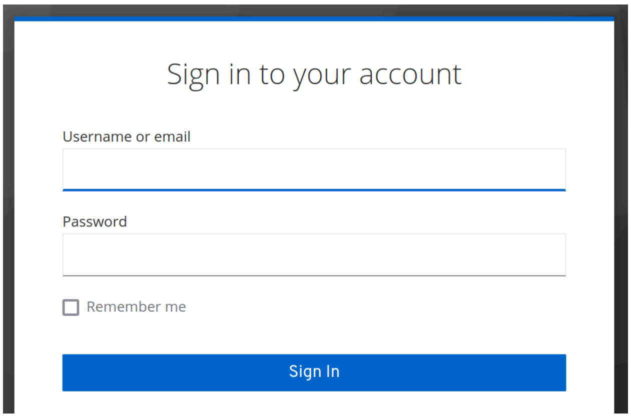

Figure 1. PretoxTM regular login.

Access to PretoxTM from the eTRANSAFE ToxHub

Important: This option is relevant when using PretoxTM in the context of the eTRANSAFE ToxHub.

PretoxTM is accessible from the principal ToxHub dashboard. Figure 2 shows the set of tools available at the ToxHub, highlighting the PretoxTM Web App.

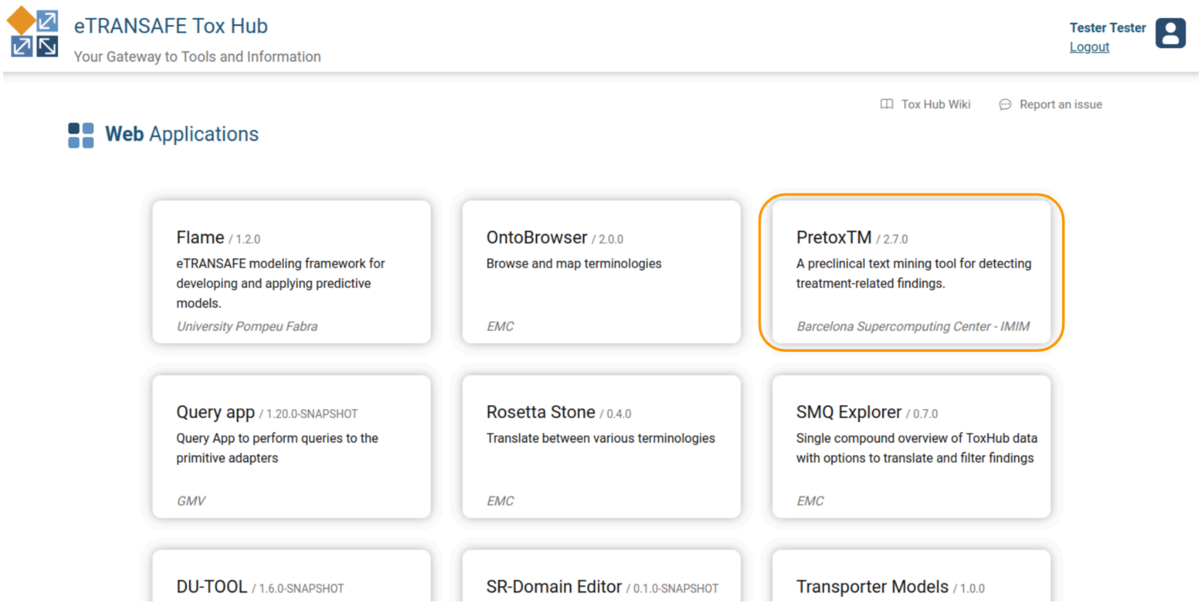

Figure 2. PretoxTM access from the ToxHub dashboard.

Home

The PretoxTM homepage consists of a list of toxicological reports that are currently being processed (Figure 3). The columns of the table indicate some features of each report: the filename (Name), when it was uploaded (Upload Date), who uploaded it (User) and the current status (Status). Additionally, in the last column there is a set of icons that are explained in their corresponding section of the manual.

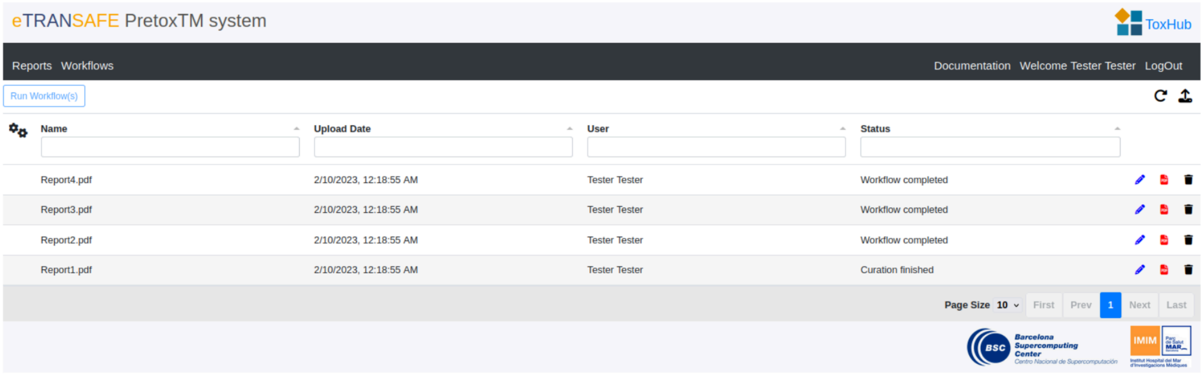

Figure 3. PretoxTM Web App homepage. The list of reports that are being processed is shown in the table.

The report table provides common functionalities such as sorting and filtering by column value at headers. Besides, pagination allows the user to navigate easily through the data and to define the page size at the bottom of the table.

Prompt Execution

Important: This option is available when using PretoxTM outside the eTRANSAFE ToxHub environment.

The user can access the “Prompt Execution” menu to enter text in a designated field and then execute the workflow (Figure 4). This feature provides a convenient way to rapidly test the capabilities of the tool. In this scenario, the system automatically generates a document in TXT format, which is listed as a regular PretoxTM document.

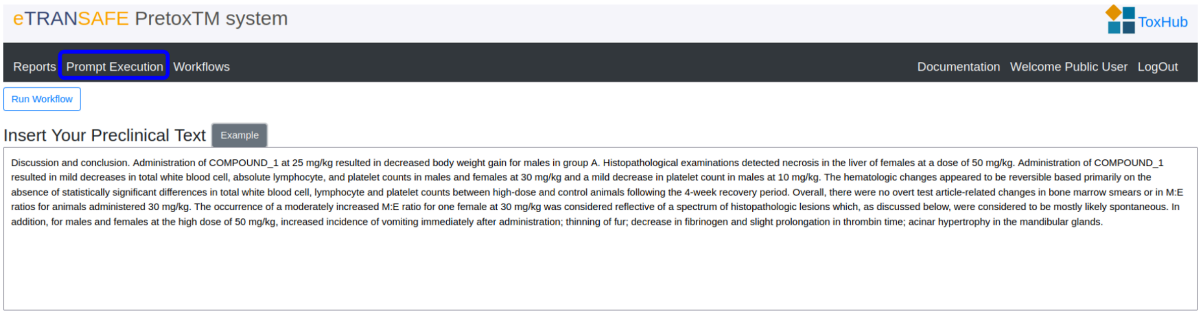

Figure 4. Prompt Execution Example.

Upload Reports

The user can upload one or more study reports in PDF format using the upload icon located at the top right of the page (Figure 5). Once the reports have been uploaded, a dialogue box will appear to select which reports to process immediately afterwards (Figure 6).

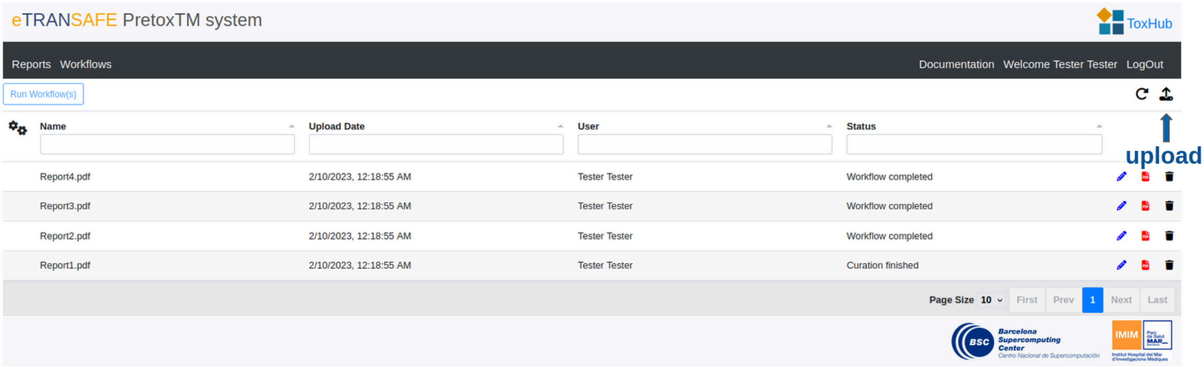

Figure 5. Upload Reports Example.

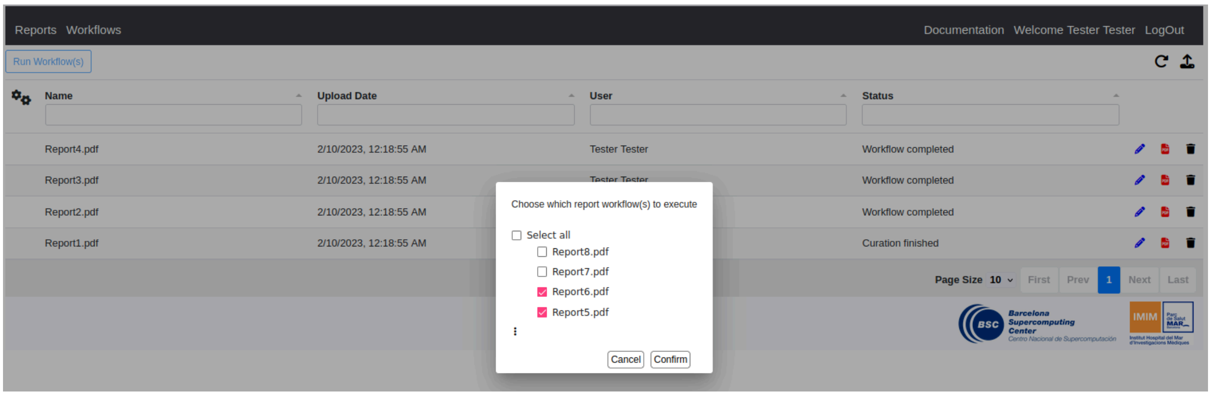

Figure 6. PretoxTM upload dialogue box to process reports with the PretoxTM pipeline.

Only the selected reports will be processed by the PretoxTM pipeline; the rest will only be uploaded to the system and the "Uploaded" status will be assigned to them.

PretoxTM pipeline

Run Workflow

The user can run the PretoxTM pipeline in two manners:

1) After uploading the reports as it was explained in the previous section.
2) Selecting one or more uploaded reports (i.e. "Uploaded" status) at the left of the table and then clicking on the "Run Workflow" button (Figure 7).

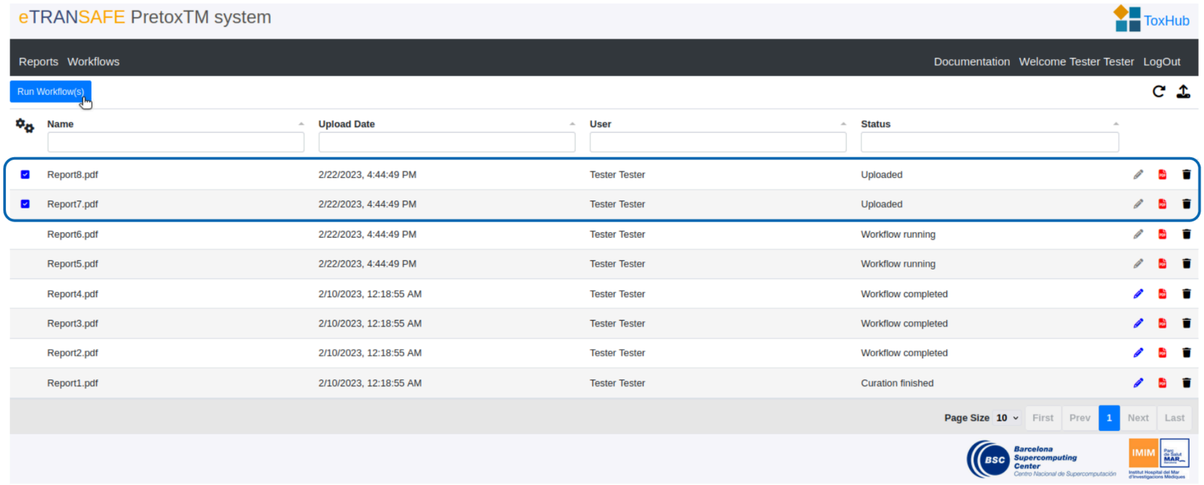

Figure 7. Uploaded reports selected to be processed by the pipeline after clicking on "Run Workflow".

Once the user has launched the pipeline, the status of the report will change to "Workflow running" and the execution information will appear on the Workflows tab (Figure 8).

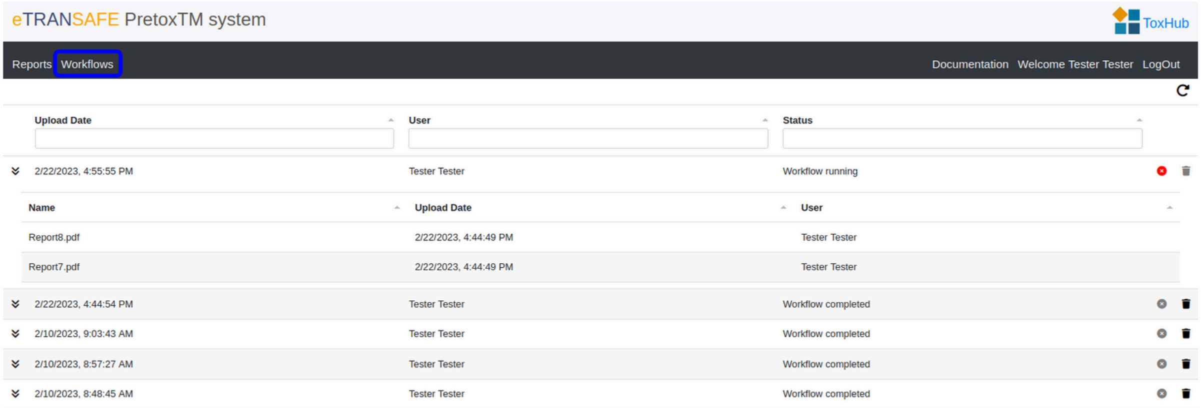

Figure 8. Workflows tab shows information about workflows and their involved reports.

The Workflows table shows when it was started (Upload Date), the user who ran it (User) and the current status of the workflow (Status).

Workflow execution should be limited to 10 reports to avoid problems while running the pipeline. For instance, if the user wants to process 50 study reports, it is recommended to launch 5 workflows with 10 reports each. See section "Workflow failed status" for further details.

Report and Workflow status

The reports have different statuses during the digitisation cycle:

1. When a report is uploaded, the status "Uploaded" is assigned. In this status, the report can be selected for workflow execution.
2. When a workflow is started, the reports that are part of that workflow enter into the "Workflow running" status.
3. When a workflow is launched and there is another workflow already in progress ("Workflow running"), the workflow and the reports involved are assigned with the "Workflow waiting" status. Workflows are therefore queued until the previous one has finished.
4. When the workflow is finished successfully, the reports involved will enter into the "Workflow completed" status.

No sections detected

The workflow attempts to detect relevant sections that contain the following names: abstract, synopsis, summary, discussion, conclusion, results, evaluation and targets for toxicity. Otherwise, the execution is interrupted and the report status is set to "No sections detected". To ensure correct section recognition, reports should follow the same structure as those included in the text mining pipeline development. A workaround in order to process reports with a different structure is shown in the next section.

Run without section extraction

The PretoxTM pipeline offers an option that could be used in cases in which the pipeline is not able to identify relevant sections. Reports with the status “No sections detected” can be executed again with the option “Run with no section extraction” (Figure 9). This should be used with caution as the entire report will be processed looking for findings, resulting in an overhead execution in large reports.

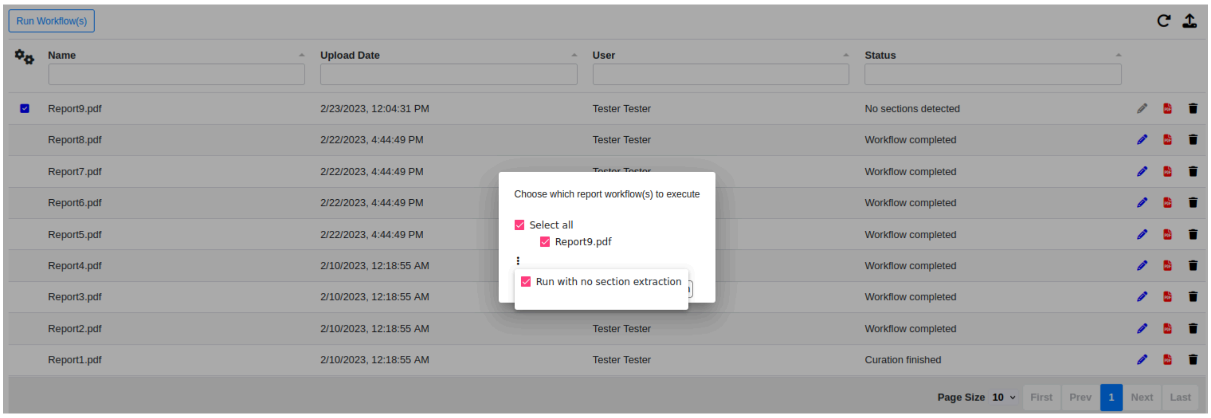

Figure 9. Run with no section extraction. Only should be used for reports with the “No section detected” status assigned.

Workflow failed status

A workflow may fail at some point in its execution. Then, all the reports involved will be assigned the status “Workflow failed”. This abnormal termination of the workflow could occur due to a problem within the text mining pipeline or because the execution reached the time limit (6 hours). This limit aims to control if there is a "problematic" report that is consuming too many resources. Problematic reports could be full scanned reports with many pages. In those cases, a TESSERACT OCR function is executed to convert those images into textual pages. This functionality is time-consuming and depends on the number and quality of pages.

It can take more than 10 minutes for some scanned pages to be converted into text. A time limit of 20 seconds is set to skip this type of pages during the execution of the pipeline. Nevertheless, some old reports contain a lot of these "dangling images” and even with the 20 seconds limit, 6 hours are consumed doing the OCR operation. Thus, this problem arises when the user includes in a workflow execution an old scanned report with a large number of pages (> 200).

It should also be noted that if a workflow fails, the execution will stop and all the reports included in the execution will be assigned the “Workflow failed status”. It is therefore advisable to run the reports described above separately, so as not to impede the correct processing of the other reports.

Open and validate report findings

After the PretoxTM pipeline execution is completed, the status of the report is updated to "Workflow completed". Then, the user can open a report by clicking on the blue pencil icon to visualise and review the detected findings. When any user opens a report the status will automatically be set to "Curation in progress".

Findings table

The first information that we see when we open a report is a table of findings (Figure 10). In this table, the information retrieved by the PretoxTM pipeline to describe an abnormal observation is presented. An abnormal effect encloses several named entities; the most relevant one is the abnormal effect detected, which depending on the **Domain** of the finding can be given by a measurement, test, or examination named **Test Name** and an abnormal **Manifestation** result obtained for that study test; or by an abnormal **Finding** in study domains where there is no associated test or measurement (e.g. clinical, macroscopic and microscopic). Other related named entities that could be present to complete the treatment-related finding are; the **Specimen** of the abnormal observation, the **Sex** of the subject, the **Group** of subjects in which the observation was detected and the **Dose** level administration of the compound. Finally, the table indicates if the abnormal observation is treatment-related or not.

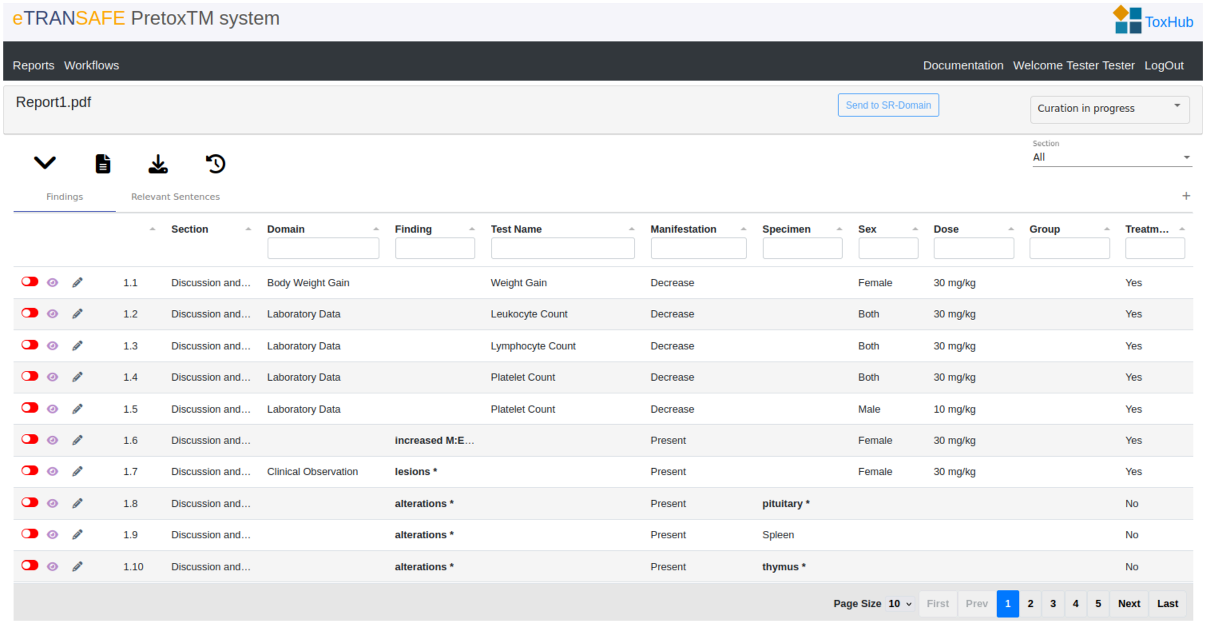

Figure 10. Table of findings in a PretoxTM report.

Textual evidence

In order to visualise PretoxTM textual evidence of the extracted findings, the user must choose a specific section of interest (Figure 11). In this example, "Discussion and conclusions" is selected and its textual evidence is shown. In the findings table only the findings of the selected section appear.

The textual evidence includes the name of the entities and also the relations between them.

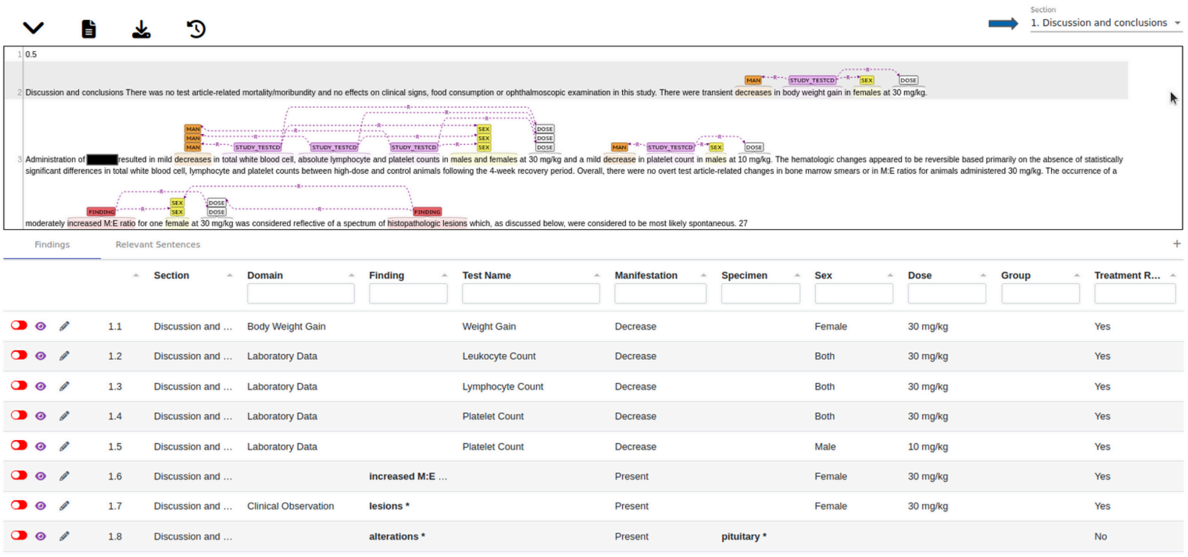

Figure 11. Textual evidence of extracted findings.

The user can visualise the textual evidence of a specific finding of interest. In the first column, each finding has an eye icon that displays the textual evidence only for that finding.

Validate the extracted findings

The user has a mechanism to validate the findings, supported by the textual evidence showing the context of the findings within the report (Figure 12). In the first column, each finding has a slide-check to indicate whether the finding is accepted (green) or not (red). By default, all the findings are marked as not valid. In this example, the first three findings are accepted.

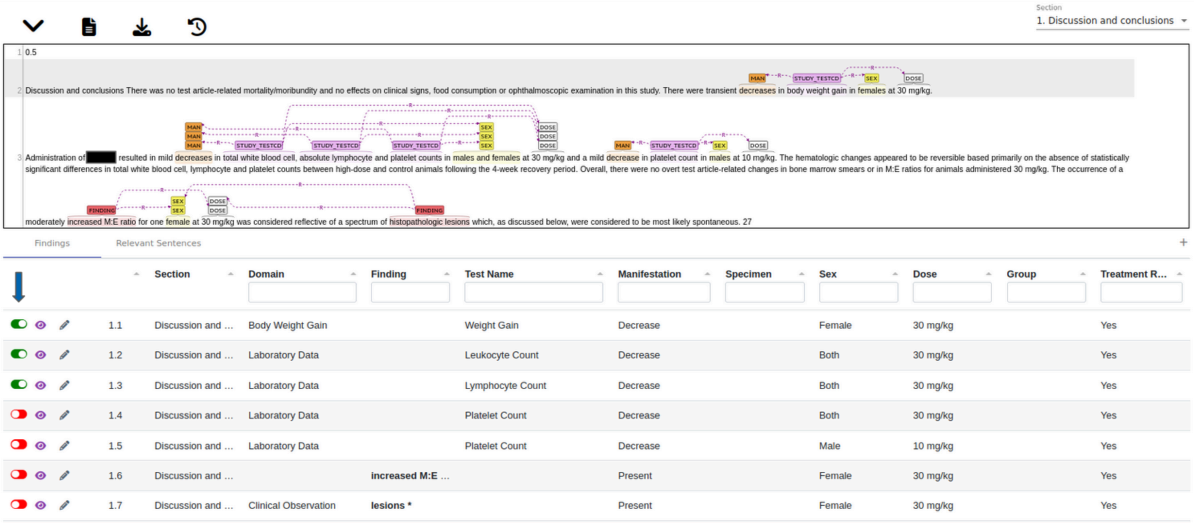

Figure 12. Finding validation functionality.

Non-controlled terminology

PretoxTM pipeline recognised entities are not always part of the CDISC SEND controlled terminology. In those cases text is bold and an asterisk appears next to the term (Figure 13).

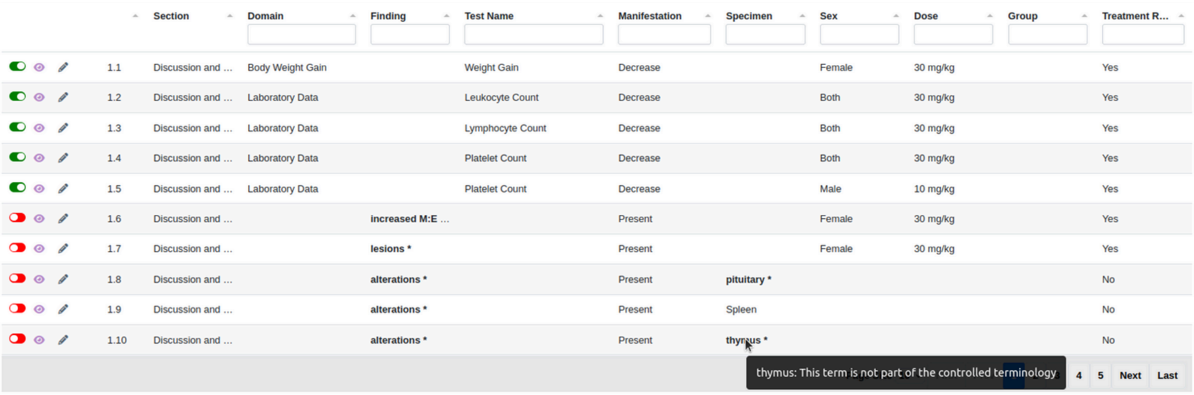

Figure 13. Non-controlled terms in findings table.

Non-controlled terms could appear in the Finding and Specimen entities. To modify this information the user can edit these entities to select a controlled term (see "Edit findings" section).

Edit findings

The user can edit the information of a non-validated finding. In the first column of the findings table, the pencil icon allows editing of the corresponding row. The user can then modify the values of the fields by clicking on them (Figure 14). Afterwards, the information can be saved or cancelled by clicking the appropriate button in the first column.

Modifications made by editing a finding will not affect the textual evidence.

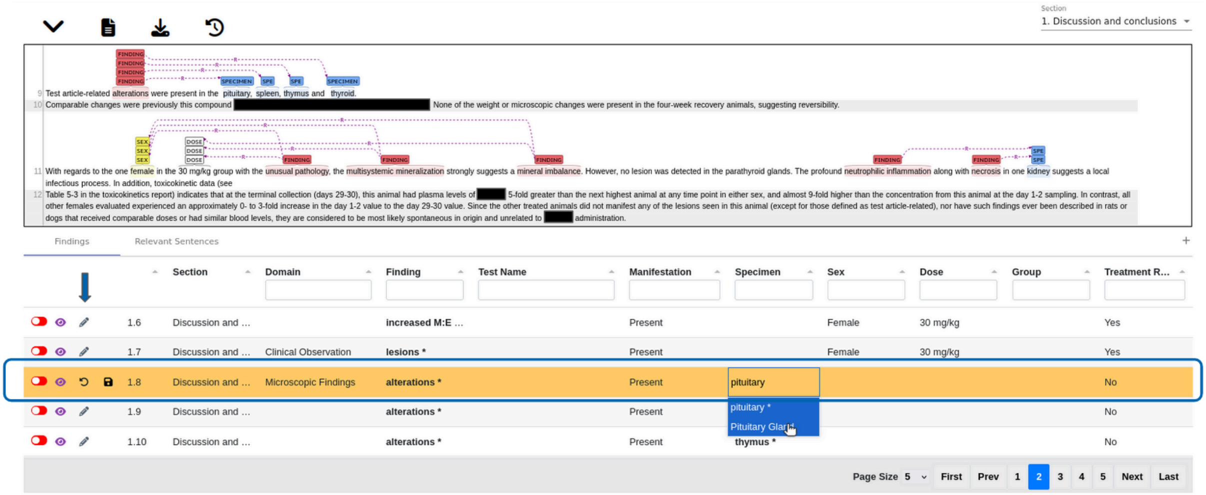

Figure 14. Edit finding functionality.

Add new findings

The user can also add new findings. Above the table, on the right-hand side, there is a plus icon to add a new finding to the table (Figure 15). Previous to the generation of a new finding, the user must select a section. The new finding entry will appear in the first row of the table, with all the fields empty except for the section. Then, the user can complete the rest of the information by editing the finding.

New findings will not have textual evidence since they were not detected by the pipeline.

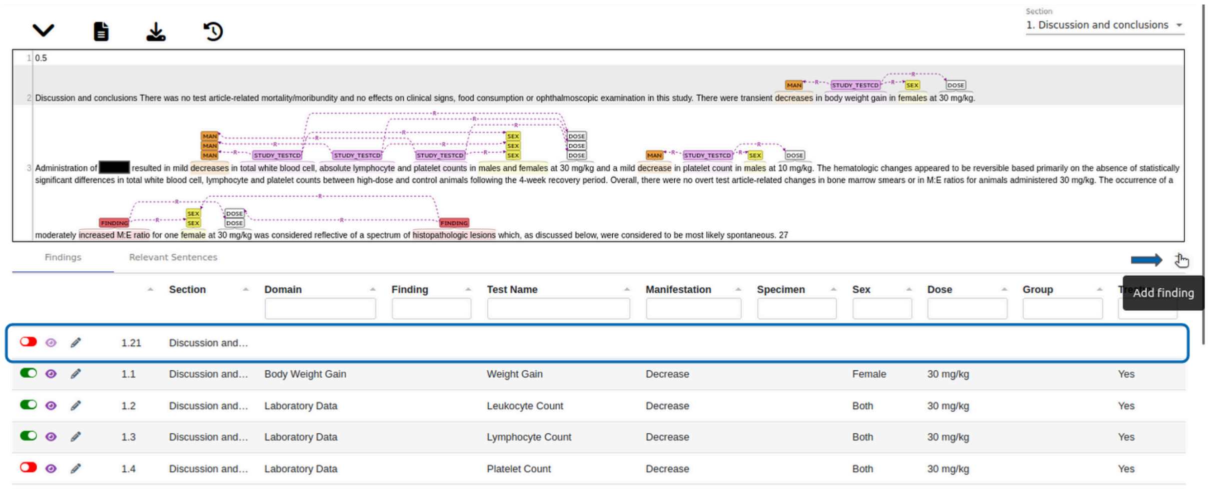

Figure 15. Add finding functionality.

Submit information to the SR-Domain Web Editor

Important: This option is relevant when using PretoxTM **within** the eTRANSAFE ToxHub environment.

During the curation of the findings the status of the report should stay in "Curation in progress". Once the user finalises the curation of the findings, the status of the report can be set to "Curation finished". In this status only validated findings are shown and they cannot be edited nor rejected. Then, the "Send to SR-Domain" button will be enabled to commit the accepted findings to the SR-Domain Web Editor (Figure 16). Once the report is submitted, it will automatically be assigned the "Curation closed" status, in which findings can only be visualised.

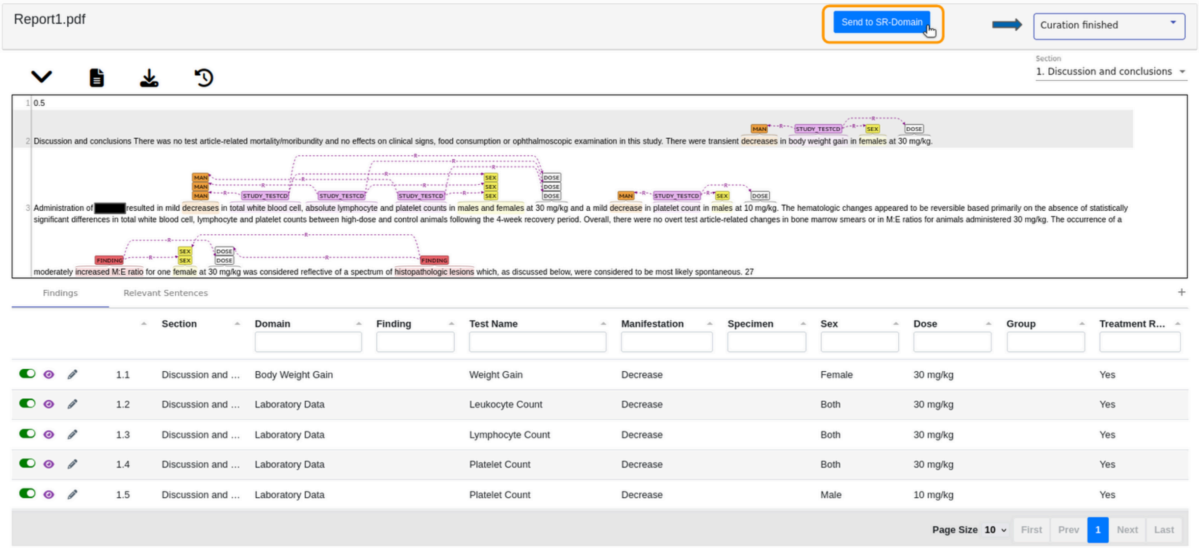

Figure 16. Send finding to the SR-Domain Web Editor application.

Before sending the information to the SR-Domain Web Editor tool, there are two validations:

- The findings must have a domain.
- The findings specimen field must have a value that is part of CDISC SEND controlled terminology.

It is also recommended to fill in the FINDING, STUDY TEST and MANIFESTATION with controlled terminology values if possible, in case the PretoxTM pipeline was not able to retrieve some of these fields.

If there is a validation problem, feedback is returned to the user to correct and complete the findings affected (Figure 17).

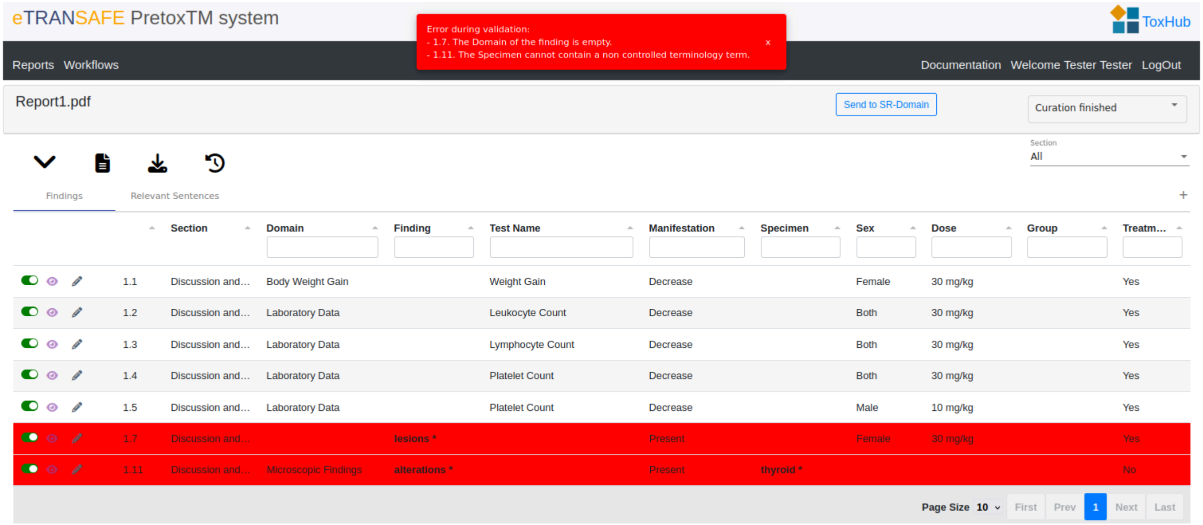

Figure 17. Validation feedback example. Information regarding missing and incorrect information is presented to the user.

In the example, finding 1.7 does not contain a domain value and finding 1.11 has a specimen (*thyroid **) which is a non-controlled terminology term. This information should be edited to correct the inconsistencies.

Export findings

Users can export validated findings in CSV format via the download button in the report menu (Figure 18). This action will generate two files: one CSV file containing the abnormal observations (Figure 19) and another CSV file with the SR-Domain template (Figure 20). The first file provides a user-friendly, readable format, while the second is more structured, displaying the codes from the SEND controlled terminology.

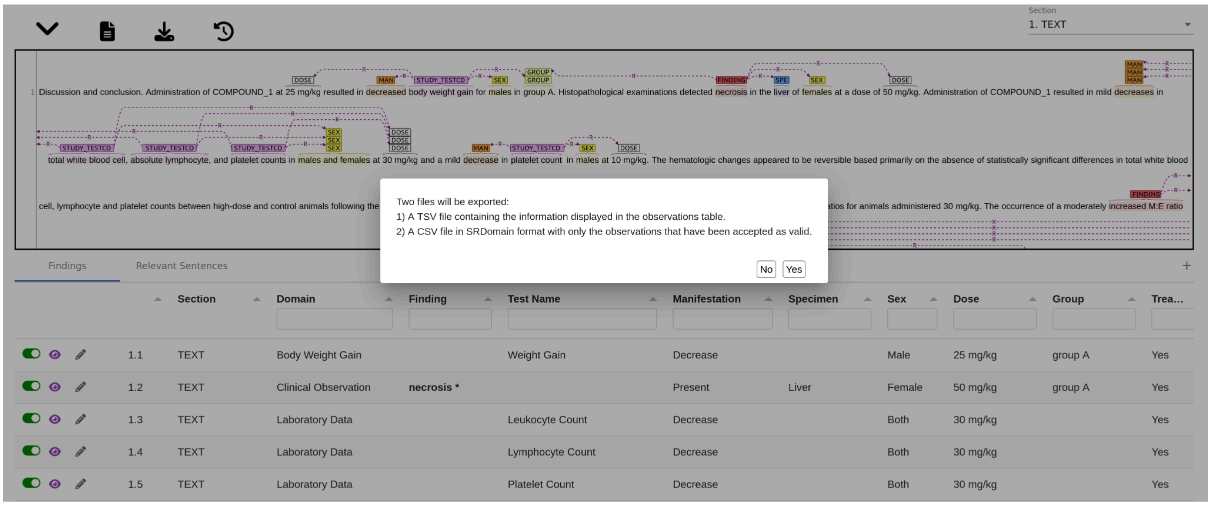

Figure 18. Export Findings Option.

Id	Section	Domain	Finding	Study_Test	Manifestation	Specimen	Sex	Dose	Group	Treatment_Related
1.1	TEXT	Body Weight Gain		Weight Gain	Decrease		Male	25 mg/kg	group A	Yes
1.2	TEXT	Clinical Observation	necrosis*		Present	Liver	Female	50 mg/kg	group A	Yes
1.3	TEXT	Laboratory Data		Leukocyte Count	Decrease		Both	30 mg/kg		Yes
1.4	TEXT	Laboratory Data		Lymphocyte Count	Decrease		Both	30 mg/kg		Yes
1.5	TEXT	Laboratory Data		Platelet Count	Decrease		Both	30 mg/kg		Yes
1.6	TEXT	Laboratory Data		Platelet Count	Decrease		Male	10 mg/kg		Yes

Figure 19. An example of exporting a list of abnormal observations into a basic standardised format.

DOMAIN	SRSEQ	SRRISK	SPGRPCD	GRPLBL	SRGRPDOS	SRSEX	SRDOMAIN	SRSPEC	SRTSTCD	SRFNDG	SRORES	SROBSV	SRTRTEF	SRCOMNT
SR	1		group A		25 mg/kg	M	BG		BWGAIN			D	Y	
SR	2		group A		50 mg/kg	F	CL	LIVER			necrosis	P	Y	
SR	3				30 mg/kg	B	LB		WBC			D	Y	
SR	4				30 mg/kg	B	LB		LYM			D	Y	
SR	5				30 mg/kg	B	LB		PLAT			D	Y	
SR	6				10 mg/kg	M	LB		PLAT			D	Y	

Figure 20. An example of exporting a list of abnormal observations into the SR-Domain format.

Relevant Sentences

There is another option to analyse the PretoxTM findings extraction: by relevant toxicological phrases. The user can click on the relevant sentences tab and a table of sentences containing the findings will appear. If a section is selected, the textual evidence of the sentences will be displayed at the top (Figure 22).

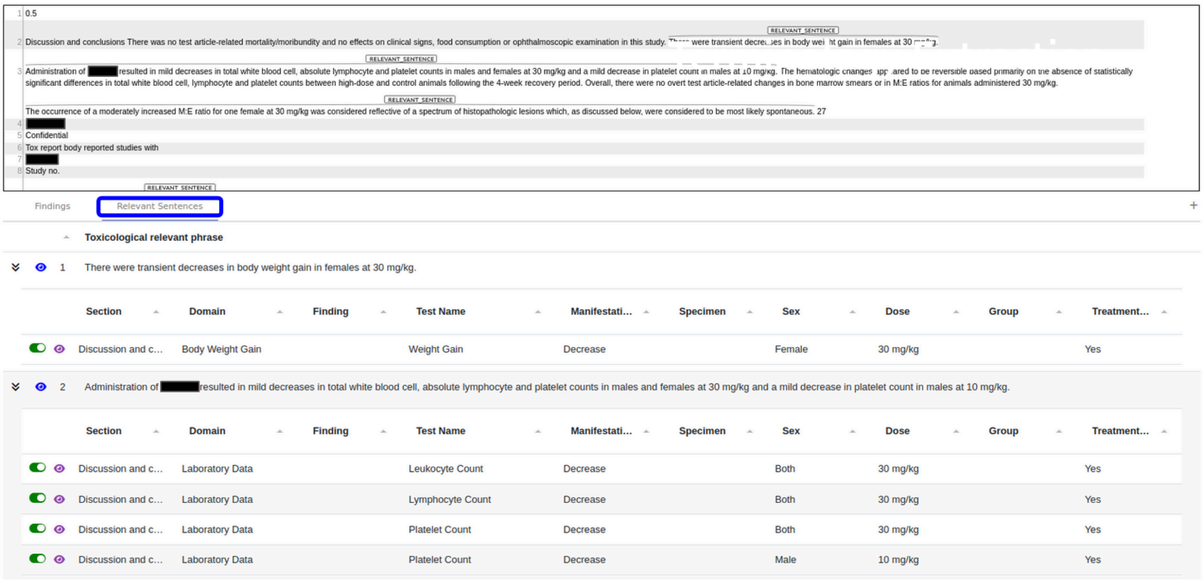

Figure 22. Relevant sentences table and textual evidence.

Historical changes

The system keeps a log of the operations performed on the report. This information can be accessed using the history changes button in the report menu. A table displays information on the date, user and action that was performed (Figure 23).

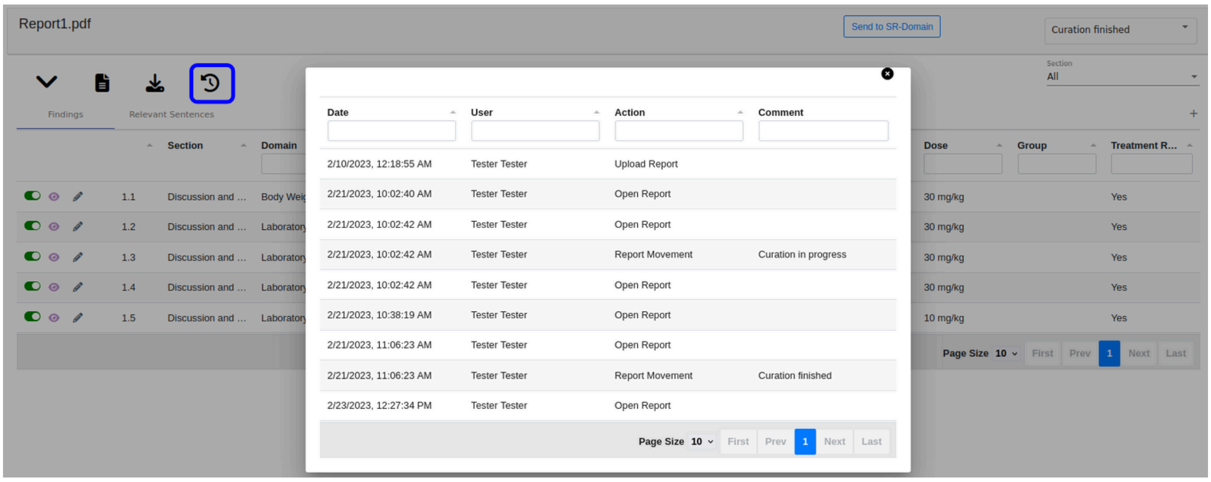

Figure 23. Historical changes table summary.

Plain Text

There is an option in the report menu that permits the user to retrieve the plain text with no annotations. A section must be selected to load the textual information.

Remove Reports

Users can remove a report using the delete button on the right of the table of reports. This operation cannot be undone as deleted reports are permanently removed.

Open PDF or Txt documents

Users can open an entire PretoxTM report on a new tab by clicking on the pdf or txt icon on the right of the table of reports.
